# Fine-Tuning of Nonlinear Optical Contrasts of Hexaphyrin-Based Molecular Switches Using Inverse Design

**DOI:** 10.3389/fchem.2021.786036

**Published:** 2021-12-03

**Authors:** Eline Desmedt, Tatiana Woller, Jos L. Teunissen, Freija De Vleeschouwer, Mercedes Alonso

**Affiliations:** General Chemistry - Eenheid Algemene Chemie (ALGC), Department of Chemistry, Vrije Universiteit Brussel, Brussels, Belgium

**Keywords:** (time-dependent) density functional theory, nonlinear optical properties, molecular switches, expanded porphyrins, inverse design, best-first search algorithm

## Abstract

In the search for new nonlinear optical (NLO) switching devices, expanded porphyrins have emerged as ideal candidates thanks to their tunable chemical and photophysical properties. Introducing *meso*-substituents to these macrocycles is a successful strategy to enhance the NLO contrasts. Despite its potential, the influence of *meso*-substitution on their structural and geometrical properties has been scarcely investigated. In this work, we pursue to grasp the underlying pivotal concepts for the fine-tuning of the NLO contrasts of hexaphyrin-based molecular switches, with a particular focus on the first hyperpolarizability related to the hyper-Rayleigh scattering (*β*_*HRS*_). Building further on these concepts, we also aim to develop a rational design protocol. Starting from the (un)substituted hexaphyrins with various *π*-conjugation topologies and redox states, structure-property relationships are established linking aromaticity, photophysical properties and *β*_*HRS*_ responses. Ultimately, inverse molecular design using the best-first search algorithm is applied on the most favorable switches with the aim to further explore the combinatorial chemical compound space of *meso*-substituted hexaphyrins in search of high-contrast NLO switches. Two definitions of the figure-of-merit of the switch performance were used as target objectives in the optimization problem. Several *meso*-substitution patterns and their underlying characteristics are identified, uncovering molecular symmetry and the electronic nature of the substituents as the key players for fine-tuning the *β*_*HRS*_ values and NLO contrasts of hexaphyrin-based switches.

## 1 Introduction

Scientists’ searching for new and innovative materials with promising properties often finds their way back to the roots and fundamentals of nature. Porphyrins, for example, are engaged in many essential and complex processes of nature, such as photosynthesis or oxygen fixation in blood cells. Hence, porphyrins are excellent candidates as natural products to design novel compounds with a high adaptability to their environment (Kadish et al., 2000; Dolphin, 2012; Auwärter et al., 2015). Based on these tetrapyrrolic macrocycles, expanded porphyrins have been synthesized with an array of interesting properties, including a high conformational flexibility, novel coordination behaviours, versatile aromaticity and exceptional optical properties (Tanaka and Osuka, 2016). Expanded porphyrins are macrocycles containing more than four pyrrole rings (or analogous heterocyclic subunits), which are connected directly or by methine groups, keeping an internal pathway of at least 17 atoms (Sessler and Seidel, 2003). Different modifications to the core structure of expanded porphyrins can be introduced, either by 1) replacing one or more pyrrole rings with other five-membered heterocycles such as furan, thiophene or tellurophene (Chandrashekar and Venkatraman, 2003; Rath et al., 2005; Kumar et al., 2007; Chatterjee et al., 2017), or by 2) introducing different substituents at the *meso*-positions of the macrocycle (Marcos et al., 2014; Tanaka and Osuka, 2016; Woller et al., 2016; Torrent-Sucarrat et al., 2017b). These variations in the core structures have recently drawn a lot of attention as the modified structures exhibit outstanding properties for multidisciplinary applications such as bio-sensors, photodynamic therapy, photochemistry, catalysis and molecular electronics (Saito and Osuka, 2011; Sung et al., 2017; Cárdenas-Jirón et al., 2019).

The field of molecular electronics involves the use of individual molecules as active elements in the electronic circuit, such as diodes, rectifiers, transistors, or memory devices (Sun et al., 2014; Su et al., 2016; Xiang et al., 2016). Besides their potential to meet the ever-increasing demand for the miniaturization of electronics, more importantly, molecular electronics opens up the possibility of devices with novel functionalities beyond the complementary silicon-based technologies, such as molecular switches (Zhang et al., 2015). A considerable advantage over silicon-based electronics is that molecular electronics can be tailor-made using the concepts from self-assembly and thus becomes highly compatible with a large number of substrates (Su et al., 2016). As a consequence, the chemical and physical properties are easily tunable resulting in applications ranging from photopharmacology and drug delivery to imaging, spectroscopy and optoelectronics (Feringa and Browne, 2011; Feringa, 2017).

The switching devices consist of a molecular building block that can be reversibly shifted between two or more stable states upon application of an external stimulus, including light, temperature, bias voltage, chemical reactions and even mechanical forces (Zhang et al., 2015; Ladenthin et al., 2016). The prerequisite of a molecular switch is that the two or more states are separable and show a distinct difference in the property of interest (Zhang et al., 2015). Regarding optical switches, organic materials with commutable nonlinear optical responses are sought for optoelectronic applications, such as molecular-scale memory devices with multiple storage and non-destructive reading capacity (Kawata and Kawata, 2000). Interesting nonlinear (NLO) optical properties are second harmonic generation (SHG), two-photon absorption (TPA), and third harmonic generation (THG), among others. At the molecular level, the first hyperpolarizability (*β*) is a noteworthy quantity as this property is intimately connected to a variety of optical phenomena like SHG or the Hyper-Rayleigh Scattering (HRS). The potential of single molecules as nonlinear optical switches is mainly determined by the amplitude of the NLO contrast and thus large differences in first hyperpolarizability between the different states are required for high-performance optical switches (Castet et al., 2013; Kariduraganavar et al., 2021).

Expanded porphyrins are highly desirable for near-infrared dyes and nonlinear optical applications (Rath et al., 2005; Pawlicki et al., 2009; Mori et al., 2013; Shimomura et al., 2020; Wang et al., 2020). Thanks to their extended *π*-system, expanded porphyrins experience a red-shift of absorption bands and exhibit a much larger TPA cross section compared to regular porphyrins. As expanded porphyrins can easily switch between different redox states and *π*-conjugation topologies induced by diverse external stimuli, they are an ideal test bed for molecular optical switches (Tanaka and Osuka, 2016). Indeed, recent computational studies have shown that the interconversion between different *π*-conjugation topologies, more precisely, Hückel and Möbius structures, induces dramatic changes in first and second hyperpolarizabilities, acting therefore as a novel type of efficient nonlinear optical switches (Torrent-Sucarrat et al., 2017b; Woller et al., 2019). Importantly, the performance of these optical switches can be improved by introducing different types of functionalizations (Torrent-Sucarrat et al., 2017b; Pino-Rios and Cárdenas-Jirón, 2019; Woller et al., 2019). For example, it has been demonstrated that the NLO contrast in [28]hexaphyrin topological switches can be enhanced by introducing push-pull substitution patterns, which incorporate electron-withdrawing and electron-donating substituents on *meso*-positions located on the opposite sides of the macrocycles (Torrent-Sucarrat et al., 2017b).

However, exploring the entire region of synthetically accessible expanded porphyrins in the chemical compound space (CCS) remains an immense challenge. Hence, a rational approach is needed to help in the design of new high-potential molecular switches. In traditional molecular design procedures, one directly introduces the desired functionalizations into the chemical structure and subsequently evaluates for the altered structure the NLO quantities (Torrent-Sucarrat et al., 2012; Torrent-Sucarrat et al., 2017b; Tonnelé et al., 2018; Woller et al., 2019). One thus mainly relies on the chemist’s intuition to design derivatives with favorable NLO properties, which can become a cumbersome task when multiple chemical modifications are demanded. Alternatively, inverse molecular design approaches target the optimization of the property of interest as a function of the chemical structure (Wang et al., 2006; Balamurugan et al., 2008; De Vleeschouwer et al., 2012; De Vleeschouwer et al., 2015; De Vleeschouwer et al., 2016; Teunissen et al., 2017; Freeze et al., 2019). Only modifications are made that effectuate property enhancement. A big advantage of these inverse design approaches is that only a small subset of all possible expanded porphyrin derivatives needs to be evaluated to devise high-performing chemical structures for a particular application. Recent advances in computational inverse design approaches have already impacted the pool of structures available to nanophotonics (Molesky et al., 2018; Su et al., 2020).

**SCHEME 1:**
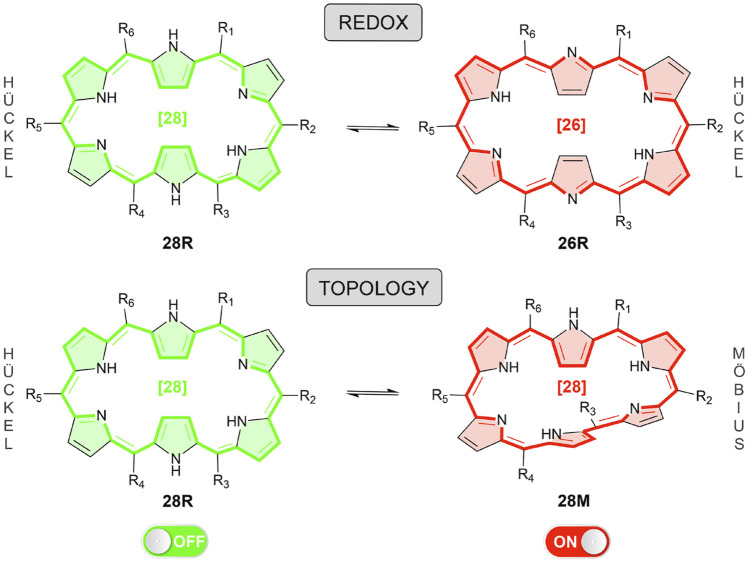
Overview of the redox and topology switches based on hexaphyrins. Inverse design is applied to optimize the functionalization of both molecular switches by maximizing the NLO contrasts. Red five-membered rings are linked to aromatic structures, whereas green ones to antiaromatic configurations.

In this paper, we take up the challenge to design efficient molecular NLO switches by means of an inverse design algorithm. In this way, the discovery of new functionalized expanded porphyrins with improved NLO contrasts can be accelerated. In particular, we focus on hexaphyrins, which are very effective platforms to realize versatile electronic states including Möbius and Hückel aromatic and antiaromatic species (Sankar et al., 2008; Ahn et al., 2005; Ishida et al., 2011; Kim et al., 2009; Sun et al., 2016). Two types of hexaphyrin-based molecular switches are considered here, namely redox (**28R** → **26R**) and topology (**28R** → **28M**) switches, the latter involving Hückel-to-Möbius transformations while keeping the number of *π*-electrons constant (Scheme 1). Among the different porphyrinoids investigated so far in our research group, the redox-triggered aromaticity switches based on hexapyrrolic macrocycles exhibit the largest ON/OFF transmission ratios in conductance switching applications (Alonso et al., 2012, 2014; Alonso et al., 2013a; Alonso et al., 2013b; Woller et al., 2016; Woller et al., 2018; Stuyver et al., 2018). In addition, topology switches based on [28]hexaphyrin exhibit dramatic variations in the second- and third-order NLO properties, as shown by Torrent-Sucarrat et al. (2017b).

Our objectives are threefold. First, we aim to establish the structure-property relationships between molecular topology, aromaticity, and optoelectronic properties for different unsubstituted hexaphyrins, labelled as the parent structures. Second, we investigate the effect of selected electron-withdrawing and electron-donating *meso*-substituents on the macrocyclic aromaticity and nonlinear optical properties. Lastly, we perform the inverse molecular design procedure with the Best-First Search (BFS) algorithm (Wang et al., 2006; Balamurugan et al., 2008; De Vleeschouwer et al., 2012, 2015; De Vleeschouwer et al., 2016; Teunissen et al., 2017) to find *meso*-substitution functionalizations of hexaphyrins inducing a maximum NLO contrast. By applying the inverse design protocol, only a fraction of all possible structures is calculated. Different substitution patterns as previously described in literature are considered, involving multiple sites to be modified, from a library of possible substituents.

## 2 Theoretical Background: Inverse Molecular Design

**FIGURE 1:**
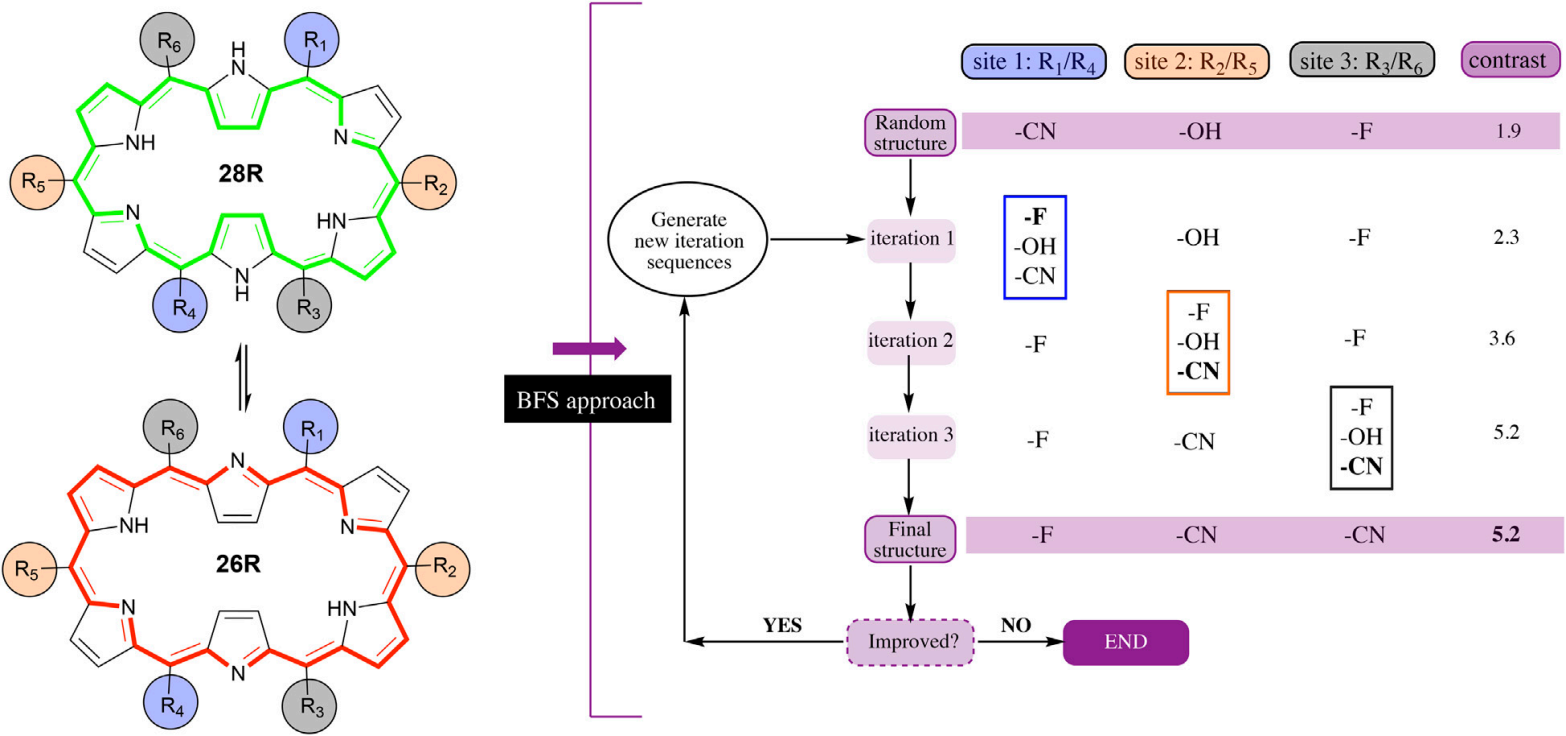
Example of the best-first search algorithm applied on the hexaphyrin-based redox switch (**26R** → **28R**) with six modifiable sites, considered pairwise, and three substituents. Note that the desired target property, *i.e.* the NLO contrast, adds to the complexity of the inverse design problem. The contrast optimization requires the evaluation of the first hyperpolarizability of the two states involved in the switching. Thus, for each functionalization, two structure optimizations are needed before evaluating the target property, increasing the computational time. Therefore, the application of an inverse design approach to the discovery of high-performance optical switches remains a challenging task.

The Best-First Search (BFS) algorithm consists of a site-by-site optimization during which chemical modifications from a fragment library are introduced into a predefined molecular scaffold (Wang et al., 2006; Balamurugan et al., 2008; De Vleeschouwer et al., 2012; De Vleeschouwer et al., 2015; Teunissen et al., 2017; De Vleeschouwer et al., 2016). For each site, the modification’s impact on the target property of interest is evaluated and the most favourable substituent is selected based on the largest improvement in property value. The algorithm relies on the independent site approximation (ISA), which assumes that the sites can be optimized one by one, yielding the optimum after a single iteration over all sites and regardless of the site sequence used. Nevertheless, for most properties, the ISA is not fully valid as sites are affected by the type of functionalization on other positions in the structure. Therefore, several site iterations are carried out to ensure that intersite influences are evaluated as well. Figure 1 shows an example of the BFS algorithm on the **28R** ⇆ **26R** redox switch with 3 pairs of sites (R_1,4_, R_2,5_ and R_3,6_) and 3 substituents (-F, -CN and -OH) available for functionalization. The three pairs of sites consist of combinations of 2 *meso*-positions that face each other on the macrocycle. First, the algorithm generates a random initial structure within the hexaphyrin CCS, with the CCS magnitude defined by the number of sites and the size of the substituent library. To this end, the algorithm randomly selects for each site a substituent from the fragment library. In the example of Figure 1, the randomly chosen substituents are a cyano group for the first (R_1,4_), a hydroxyl group for the second (R_2,5_), and a fluoro group for the third site (R_3,6_). Next, the algorithm starts to optimize site 1 (R_1,4_), while keeping all other sites (R_2,5_ and R_3,6_) fixed, *i.e.* the substituents on all sites, but site 1, remain unaltered. Optimizing site 1 involves placing each of the substituents from its fragment library on that position and evaluating the property for each generated structure. The substituent with the largest improvement in *β*_*HRS*_ contrast (here: -F) is then introduced into the initial structure. This altered structure is subsequently used as the starting point for the next step in the algorithm, corresponding to the optimization of site 2 while keeping the other two positions fixed. The same procedure is repeated until all sites have been visited. Convergence is reached when a single site modification does not result anymore in an improved property value. Even though there exist more recently introduced inverse design methods that may outperform the BFS method based on efficacy or larger CCS exploration (Elward and Rinderspacher, 2015; Strasser et al., 2016; Häse et al., 2018), BFS has the key advantage of systematically delivering essential information on how the type and location of each possible substituent influences our property of interest, since in every global iteration all substituents are tested on all available sites (Teunissen et al., 2017).

The figure-of-merit of the performance of an NLO switch is the NLO contrast as defined by Eq. 1. This contrast considers the ratio of the first HRS hyperpolarizabilities of both the ON and OFF state structures having the same *meso*-substitution pattern and has a high value for a well-performing NLO switch.

$$contrast=\frac{\beta_{HRS}\left(\text{O}\text{N}\right)}{\beta_{HRS}\left(\text{O}\text{F}\text{F}\right)}$$ (1)

In the literature, this contrast expression has been widely used for the evaluation of the performance of molecular switches (Plaquet et al., 2008; Castet et al., 2013; Tonnelé et al., 2018; Woller et al., 2019). The contrast is maximized during the BFS procedures, but the maximization of the ratio entails one problem. If the denominator becomes zero, the entire contrast reaches a value of infinity making it impossible to compare other structures facing the same issue. This widely used contrast definition may therefore not be an ideal target objective in the inverse design maximizations. To circumvent this complication, we implemented the following condition within the BFS procedure. If the *β*_*HRS*_ of the OFF state becomes lower than 10 a.u., the denominator is set to a value of 0.001 a.u.

Another measure of NLO contrast has been presented, among others, in the work of Torrent-Sucarrat et al. (2017b), taking the difference of the NLO responses between the ON and OFF state (Eq. 2). A high difference is connected to a high and low *β*_*HRS*_ response for the ON and OFF state, respectively, and reflects similar behaviour as our previous contrast definition. Note that a value of zero for the OFF state does not pose any optimization issues. The main difference between both contrast definitions is that high *β*_*HRS*_ differences can also appear from molecular switches in which the OFF state’s *β*_*HRS*_ response is far away from zero a.u. In this work, we carried out additional BFS searches in which the *β*_*HRS*_ difference is maximized. The best performing switches are then compared with the optimal structures connected to the ratio in *β*_*HRS*_.

$$contrast=\beta_{HRS}\left(\text{O}\text{N}\right)-\beta_{HRS}\left(\text{O}\text{F}\text{F}\right)$$ (2)

## 3 Methodology

All quantum-chemical calculations were executed with the Gaussian16 software package (Frisch et al., 2016). The geometry optimizations and frequency calculations were performed using the Minnesota hybrid functional M06-2X (Zhao and Truhlar, 2008) with the cc-pVDZ basis set (Dunning, 1989). In recent benchmark studies (Marcos et al., 2012; Torrent-Sucarrat et al., 2017a; Sylvetsky et al., 2020; Woller et al., 2020), the M06-2X functional emerges among the best contemporary density functionals to describe the relative energies of Hückel and Möbius expanded porphyrins. Although singly-twisted Möbius topologies are a challenging test for electronic structure methods, M06-2X provides errors close to chemical accuracy relative to the golden-standard canonical CCSD(T) calculations. Harmonic vibrational analyses show that all optimized structures are exclusively characterized by positive eigenvalues and thus represent minima on the potential energy surface. To obtain more accurate electronic energies, subsequent single-point energy calculations on the optimized geometries were performed at the M06-2X/cc-pVTZ level of theory.

**FIGURE 2:**
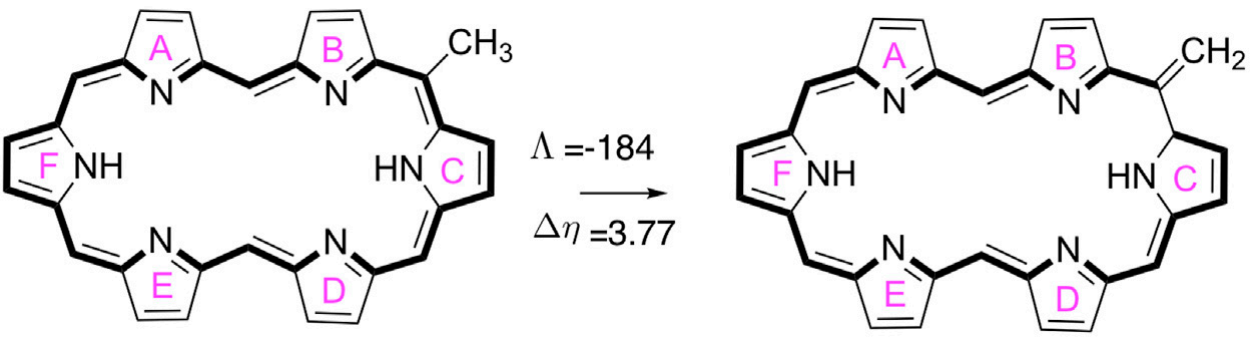
A schematic overview of the isomerization reaction used to evaluate the magnetic susceptibility exaltation (Λ in ppm cgs) and relative hardness (Δ*η* in kcal mol^−1^) for the **26D** macrocycle.

For all unsubstituted hexaphyrins, several aromaticity descriptors were computed, including the magnetic susceptibility exaltation (Λ) (Dauben et al., 1968), the nucleus-independent chemical shifts (NICS) (Lazzeretti, 2004; Chen et al., 2005; Fallah-Bagher-Shaidaei et al., 2006), the relative chemical hardness (Δ*η*) (De Proft and Geerlings, 2004), and the recently introduced AV1245 and AV_min_ (Casademont-Reig et al., 2018; Matito, 2016). This set of aromaticity descriptors are rooted in magnetic, reactivity and electronic criteria in order to take the multidimensional character of the aromaticity into account (Solà, 2017; Woller et al., 2016; Wu et al., 2013). For the evaluation of the aromaticity descriptors, we employed the long-range corrected functional CAM-B3LYP (Yanai et al., 2004), in combination with 6–311+G(d,p) (Hehre et al., 1986), due to the relevance of the delocalization error in these measures. It has been recently found that the amount of Hartree-Fock exchange in DFT functionals largely influences the value of the aromaticity descriptors in expanded porphyrin systems, although aromaticity trends remain (Casademont-Reig et al., 2018, 2020; Szczepanik et al., 2017). The global aromaticity indices, Λ and Δ*η*, were computed on the basis of the isomerization scheme (Figure 2), which involves the comparison of the magnetic susceptibilities and hardness values of a methyl and methylene isomer of each hexaphyrin structure (Schleyer and Pühlhofer, 2002; Alonso et al., 2012).

Magnetic susceptibilities were evaluated with the Continuous Set of Gauge Transformations (CGST) method (Keith and Bader, 1993), whereas the hardness of the methyl and methylene isomers was computed as the HOMO and LUMO energy difference. The Gauge-Independent Atomic Orbital (GIAO) method (Wolinski et al., 1990) was applied to obtain the NICS values at three different positions: the geometrical center of the macrocycle’s heavy atoms and at 1Å above and below the ring center, together with their respective out-of-plane tensor components. The anisotropy of the induced current density (AICD) method (Herges and Geuenich, 2001; Geuenich et al., 2005) was applied to visualize the induced delocalization of *π*-electrons. In both the NICS and AICD methods, an external magnetic field is applied perpendicular to the molecular plane for expanded porphyrins with a Hückel or Möbius topology. Recently, extensive studies have been performed on the aromaticity of both unsubstituted and substituted expanded porphyrins by means of the ring current criterion and current density analysis (Fliegl et al., 2010, 2011; Fliegl and Sundholm, 2012; Valiev et al., 2013; Valiev et al., 2017; Valiev et al., 2018; Foroutan-Nejad et al., 2018; Foroutan-Nejad and Ghosh, 2018; Ghosh et al., 2018; Conradie et al., 2019). The electronic delocalization indices AV1245 and AV_min_ indices were computed with the ESI-3D program (Matito, 2006; Casademont-Reig et al., 2018) at the CAM-B3LYP/6-311+G(d,p) level of theory. The calculation of these electronic indices relies on Quantum Theory of Atoms in Molecules (QTAIM) atomic partition performed with the AIMAll software (Keith, 2017). AV1245 consists of the average of the 4-center multicenter index (MCI) values along the ring that keeps a positional relationship of 1, 2, 4, 5, whereas AV_min_ corresponds to the minimal absolute value of the aforementioned 4-center MCI values along the conjugation pathway (Matito, 2016; Casademont-Reig et al., 2018).

Next to the aromaticity descriptors, two geometrical descriptors, *i.e.,* the torsional ring strain (Φ_*p*_) (Alonso et al., 2012) and the *π*-conjugation index (Π) (Stępień et al., 2011; Alonso et al., 2012), were computed to gain insight into the factors governing the relative energies of Hückel and Möbius conformations. Φ_*p*_ corresponds to the average dihedral angle between neighboring pyrrole rings, whereas Π measures the effectiveness of the overlap of neighboring *p*-orbitals. Π is positive for Hückel conformations and negative for Möbius structures. Macrocyclic aromaticity in porphyrinoids corresponds to values of Π above a threshold of 0.30 (Stępień et al., 2011).

Hyperpolarizabilities related to the nonlinear optical responses in the static and dynamic regimes were obtained at the coupled-perturbed Kohn-Sham and TD-DFT levels of theory, respectively, with the CAM-B3LYP functional and a Pople basis set 6-311G+(d,p). Different benchmark studies highlight the dependency of extended organic dyes’ NLO properties on the exchange-correlation functional and basis set. However, recent research has shown that range-separated hybrid functionals, such as CAM-B3LYP, are the best choice for the calculation of first order hyperpolarizabilities (Garza et al., 2015; Lescos et al., 2020). For the particular case of Hückel and Möbius *π*-conjugation topologies, Torrent-Sucarrat *et al.* showed that the hybrid M05-2X and the long-range corrected CAM-B3LYP provide a semi-quantitative description of the NLO properties at a reasonable computational cost (Torrent-Sucarrat et al., 2011; Torrent-Sucarrat et al., 2012; Torrent-Sucarrat et al., 2017b). In Torrent-Sucarrat’s work on designing topological switches based on *meso*-substituted [28]hexaphyrins with high NLO properties, both functionals together with the 6-31G basis set were employed (Torrent-Sucarrat et al., 2017b). While the good performance of M05-2X on the NLO properties can be ascribed to the large percentage of exact HF exchange (56%), the success of CAM-B3LYP is based on the introduction of a growing fraction of exact HF exchange at larger inter-electronic distances. Regarding the basis set dependency, split-valence double- or triple-*ζ* basis sets including one set of diffuse and polarization functions were shown to be generally sufficient to describe the dominant *β* tensor components and the depolarization ratios (Plaquet et al., 2008; de Wergifosse and Champagne, 2011; Castet et al., 2013). In particular, for the evaluation of the nonlinear optical properties of a Hückel-Möbius aromaticity switch of a closely related porphyrinoid A,D-di-*para*-benzi[28]hexaphyrin(1.1.1.1.1.1), Torrent-Sucarrat *et al.* showed that the increment of basis set from 6-31G to 6-311G(d) does not provoke a large variation of *α* and *γ* values (Torrent-Sucarrat et al., 2012). As such, the 6-311+G(d,p) basis set employed in our study is adequate to compute the *β*_*HRS*_ values of the Hückel and Möbius conformers of hexaphyrins. Indeed, in a very recent benchmark study on the influence of the amount of exact Hartree–Fock exchange included in the DFT functional on the magnitude of the static HRS responses, a similar basis set 6-311+G(d) was employed (Lescos et al., 2020).

In this study, we mainly focus on the first hyperpolarizability associated to the Hyper-Rayleigh scattering phenomenon, termed as *β*_*HRS*_ (Clays and Persoons, 1991; Hendrickx et al., 1998; Verbiest et al., 2009). The incoherent scattered light is at twice the optical frequency (2*ω*) of the incident laser pulse (*ω*) and its intensity is related to the first hyperpolarizability. The entire HRS intensity can be written as Eq. 3, when the incoherent scattered light is observed perpendicular to the laser’s propagation plane.

$$\beta_{HRS}\left(-2\omega ;\omega ,\omega \right)=\sqrt{\langle \beta_{ZZZ}^{2}\rangle +\langle \beta_{ZXX}^{2}\rangle }$$ (3)

$\left\langle \beta_{ZZZ}^{2}\right\rangle$ and $\left\langle \beta_{ZXX}^{2}\right\rangle$ represent the orientational averages of *β* and describe the isotropic distribution of molecular orientations. The full descriptions of these tensor components are written in Eqs. 4, 5.

$$\begin{array}{ll} \left\langle \beta_{ZZZ}^{2}\right\rangle = & \frac{1}{7}\overset{x,y,z}{\underset{i}{\sum }}\beta_{iii}^{2}+\frac{4}{35}\overset{x,y,z}{\underset{i\neq j}{\sum }}\beta_{iij}^{2}+\frac{2}{35}\overset{x,y,z}{\underset{i\neq j}{\sum }}\beta_{iii}\beta_{ijj}+\frac{4}{35}\overset{x,y,z}{\underset{i\neq j}{\sum }}\beta_{jii}\beta_{iij}\\ + & \frac{4}{35}\overset{x,y,z}{\underset{i\neq j}{\sum }}\beta_{iii}\beta_{jji}+\frac{1}{35}\overset{x,y,z}{\underset{i}{\sum }}\beta_{jii}^{2}+\frac{4}{105}\overset{x,y,z}{\underset{i\neq j\neq k}{\sum }}\beta_{iij}\beta_{jkk}+\frac{1}{105}\overset{x,y,z}{\underset{i\neq j\neq k}{\sum }}\beta_{jii}\beta_{jkk}\\ + & \frac{4}{105}\overset{x,y,z}{\underset{i\neq j\neq k}{\sum }}\beta_{iij}\beta_{kkj}+\frac{2}{105}\overset{x,y,z}{\underset{i\neq j\neq k}{\sum }}\beta_{ijk}^{2}+\frac{4}{105}\overset{x,y,z}{\underset{i\neq j\neq k}{\sum }}\beta_{ijk}\beta_{jik} \end{array}$$ (4)

$$\begin{array}{ll} \left\langle \beta_{ZXX}^{2}\right\rangle = & \frac{1}{35}\overset{x,y,z}{\underset{i}{\sum }}\beta_{iii}^{2}+\frac{4}{105}\overset{x,y,z}{\underset{i\neq j}{\sum }}\beta_{iii}\beta_{ijj}-\frac{2}{35}\overset{x,y,z}{\underset{i\neq j}{\sum }}\beta_{iii}\beta_{jji}+\frac{8}{105}\overset{x,y,z}{\underset{i\neq j}{\sum }}\beta_{iij}^{2}\\ + & \frac{3}{35}\overset{x,y,z}{\underset{i\neq j}{\sum }}\beta_{ijj}^{2}-\frac{2}{35}\overset{x,y,z}{\underset{i}{\sum }}\beta_{ijj}\beta_{jii}+\frac{1}{35}\overset{x,y,z}{\underset{i\neq j\neq k}{\sum }}\beta_{ijj}\beta_{jkk}-\frac{2}{105}\overset{x,y,z}{\underset{i\neq j\neq k}{\sum }}\beta_{iik}\beta_{jjk}\\ - & \frac{2}{105}\overset{x,y,z}{\underset{i\neq j\neq k}{\sum }}\beta_{iij}\beta_{jkk}+\frac{2}{35}\overset{x,y,z}{\underset{i\neq j\neq k}{\sum }}\beta_{ijk}^{2}-\frac{2}{105}\overset{x,y,z}{\underset{i\neq j\neq k}{\sum }}\beta_{ijk}\beta_{jik} \end{array}$$ (5)

Lastly, an inverse design procedure was executed using the best-first search (BFS) algorithm (Pearl, 1984) as implemented in our in-house program CINDES (Teunissen, 2019; Teunissen, 2016). Both definitions for the contrast are considered for each substitution pattern. The fragment library consists of -NO_2_, -CN, -F, -H, -CH_3_, -OH and -NH_2_. For all BFS optimizations, the computational approach and level of theory for the geometry optimization, frequency and NLO calculations are kept the same as aforementioned.

## 4 Results and Discussion

### 4.1 Structure-Property Relationships in the Parent Macrocycles

**SCHEME 2:**
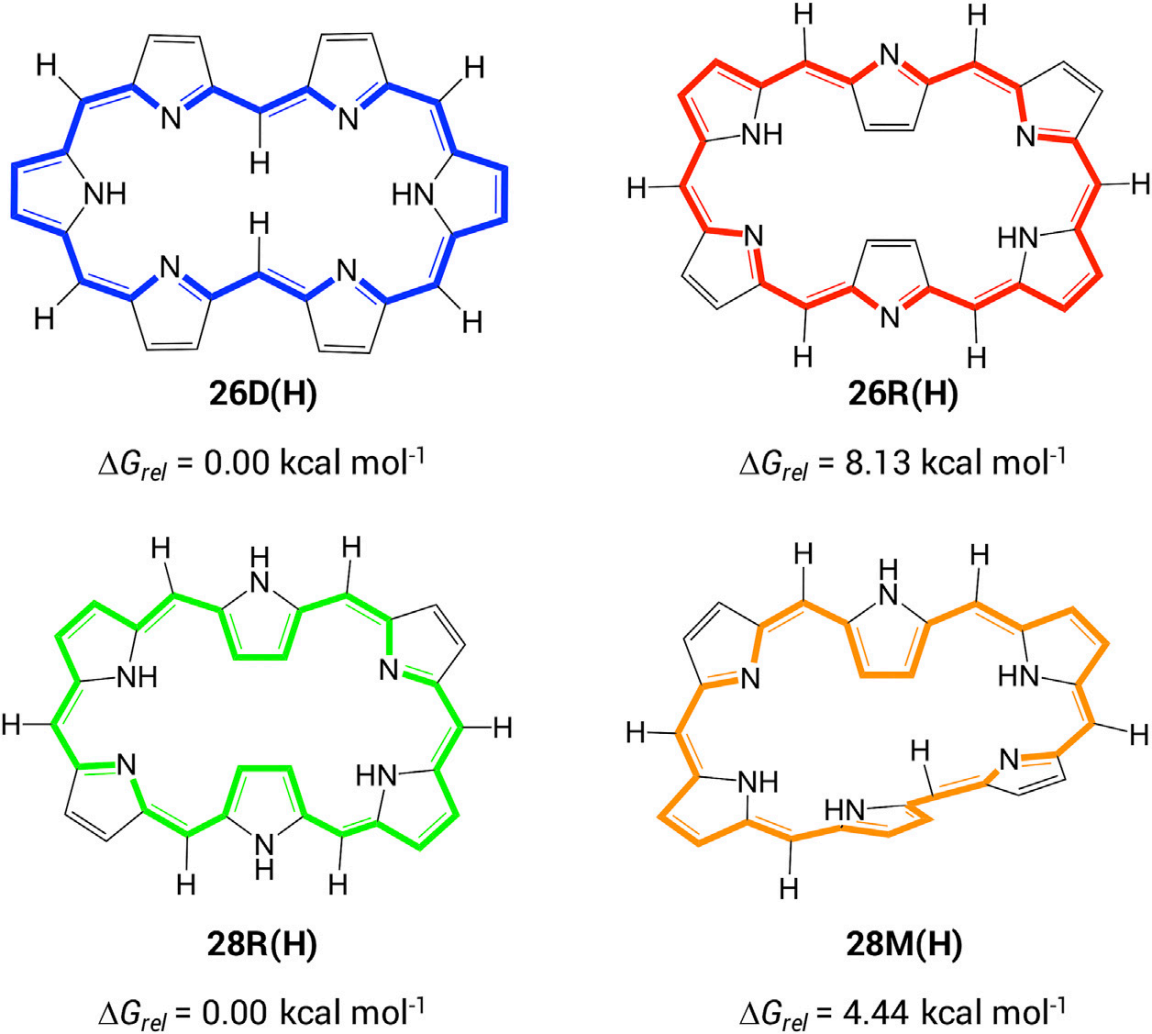
Selected conformations of [26] and [28]hexaphyrins with the corresponding relative Gibbs free energies. The classical annulene-type conjugation pathway is depicted with bold bonds.

In order to establish a relationship between molecular topology, aromaticity and NLO properties in hexaphyrin-based macrocycles, we first investigate the optoelectronic properties of unsubstituted [26] and [28]hexaphyrins. The most stable configurations for each redox state were selected based on our extensive conformational study of hexaphyrin macrocycles (Scheme 2) (Alonso et al., 2012). Each structure is labelled according to its redox state (*i.e.* the number of *π*-electrons along the annulene-type conjugation pathway) and conformation (**D**: dumbbell, **R**: rectangular and **M**: Möbius). For example, the **26D(H)** has 26 *π*-electrons and a dumbbell conformation with H-atoms on the *meso*-positions. In the case of unsubstituted macrocycles, the lowest-energy conformations for the [26] and [28]hexaphyrins correspond to **26D(H)** and **28R(H)**, respectively, which are characterized by the lowest torsional ring strain and a high degree of *π*-conjugation (Table 1). Hence, the more planar conformations are the most stable for each redox state. It is interesting to note that the **26R(H)** and **28R(H)** structures exhibit similar structural parameters despite the difference in number of *π*-electrons. Appealingly, Hückel and Möbius conformations coexist in dynamic equilibrium for the [28]hexaphyrin (Sankar et al., 2008; Kim et al., 2009) as can be inferred from the small Gibbs free energy difference between **28R(H)** and **28M(H)** (Scheme 2).

**TABLE 1:** Structural parameters, aromaticity descriptors and static and dynamic HRS first hyperpolarizability of the unsubstituted hexaphyrins^a^.

System	*ϕ*_*p*_	Π	Λ	NICS_*zz*_(1)	AV1245^b^	${AV}_{\min}$ ^b^	Δ*η*	Δ_*HL*_	$\beta_{HRS}$ ^c^		
									0	0.653	1.165
**26D(H)**	6.41	0.99	−184	−22.4	1.80	0.77	3.77	3.71	1,828	3,512	4,069
**26R(H)**	11.66	0.88	−155	−15.8	1.68	0.66	1.79	3.66	2,347	5,432	6,570
**28R(H)**	10.87	0.90	239	41.2	1.33	0.42	−11.54	3.00	∼0^d^	∼0^d^	∼0^d^
**28M(H)**	31.54	−0.45	−92	−10.7	1.63	0.79	1.68	3.82	1,470	2,311	5,679

a Ring strain (*ϕ*_*p*_ in °), torsional *π*-conjugation index (Π), relative hardness (*Δ*η in kcal mol^−1^), diamagnetic susceptibility exaltation (Λ in ppm cgs), NICS_*zz*_(1) index (in ppm), HOMO-LUMO gap (*Δ*_HL_ in eV).

b The electronic aromaticity indices AV1245 and AV_min_ were computed along the annulene conjugation pathway.

c Hyper-Rayleigh scattering first hyperpolarizability (β_*HRS*_ in a.u.) computed at different frequencies (in eV).

d *β*_*HRS*_ of the centrosymmetric **28R(H)** is not exactly zero due to round-off errors in the coordinates.

**FIGURE 3:**
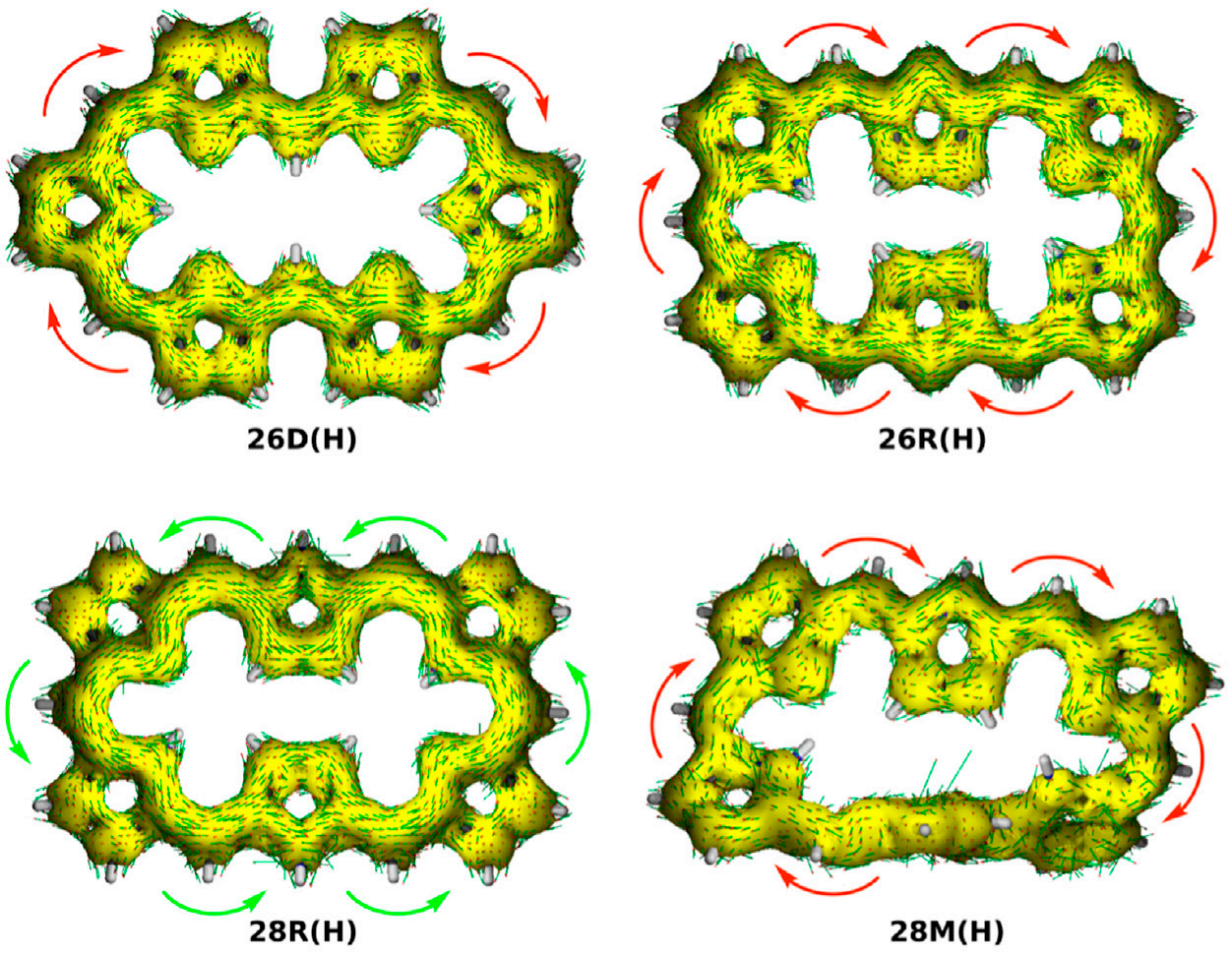
AICD isosurface plots (isocontour value 0.05) of unsubstituted [26] and [28]hexaphyrins with different conformations computed at the CAM-B3LYP/6-311+G(d,p) level of theory. The arrows denote the direction of the current density vectors.

**FIGURE 4:**
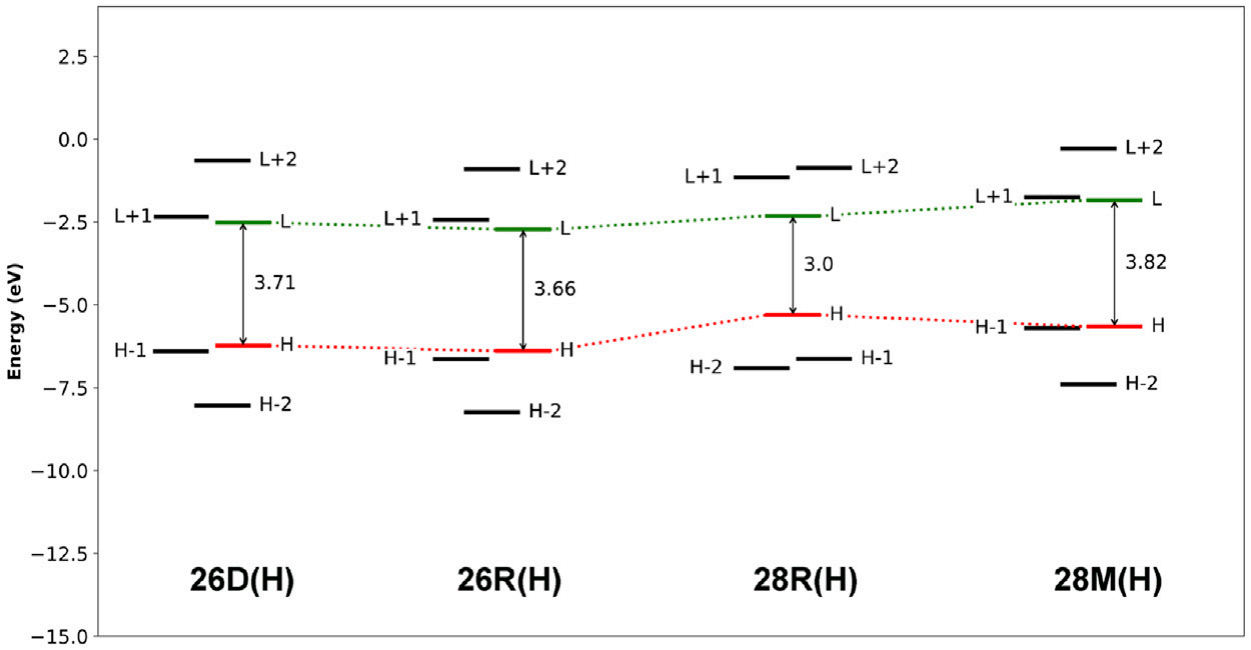
Molecular orbital diagrams computed at the CAM-B3LYP/6-311+G(d,p) level of theory.

The aromaticity of the unsubstituted conformations is quantified by means of several aromaticity indices rooted in reactivity, magnetic and energetic criteria (Table 1). Aromatic systems, such as **26D(H)**, **26R(H)** and **28M(H)**, are characterized by a positive relative hardness, a negative diamagnetic susceptibility exaltation and negative NICS indices and larger values for AV1245 and AV_min_. On the other hand, the **28R(H)** conformation shows a clear antiaromatic character with a negative relative hardness, positive magnetic descriptors and reduced values for the electronic indices. The Anisotropy of the Induced Current Density (AICD) plots (Herges and Geuenich, 2001; Geuenich et al., 2005) confirm the (anti)aromatic character of the parent structures (Figure 3). While the direction of the induced current through the aromatic structures is clockwise, the antiaromatic system **28R(H)** shows a counterclockwise current. Nicely, all the indices confirm the reversal of macrocyclic aromaticity upon redox reactions (*i.e.,* **28R(H)** → **26R(H)**) and topology interconversions (*i.e.,* **28R(H)** → **28M(H)**). The arrangement of the molecular frontier orbitals and the magnitude of the HOMO-LUMO gap (Δ_*HL*_) clearly depend on the aromaticity of the system (Figure 4). Aromatic systems (**26D(H)**, **26R(H)** and **28M(H)**) show quasi-degenerate H/H-1 and L/L+1 energy levels with larger Δ_*HL*_ gaps. On the other hand, the H-1/H-2 and L+1/L+2 energy levels are almost degenerate in the antiaromatic **28R(H)**, whereas the Δ_*HL*_ value is significantly reduced.

Regarding the nonlinear optical (NLO) properties, we focus on the first hyperpolarizability related to the Hyper-Rayleigh Scattering (*β*_*HRS*_). The *β*_*HRS*_ is evaluated in both the static and dynamic regime at two different wavelengths, 1,064 nm and 1,907 nm, corresponding to the Nd:YAG and Ho:YAG lasers, respectively. The *β*_*HRS*_ values for the unsubstituted hexaphyrins in static and dynamic regime at the two laser frequencies are tabulated in Table 1. It should be noted that the *β*_*HRS*_ is highly dependent on the centrosymmetry of the system (Woller et al., 2019). If the structure is centrosymmetric, the *β*_*HRS*_ value is zero. This is the case for the **28R(H)** conformation, which contains an inversion point and belongs to the *C*_*i*_ point group. All other conformations are within the *C*_1_ point group. On top of the centrosymmetry, the torsional strain of the molecular topology is also an underlying factor regulating the *β*_*HRS*_ response. When the topology shifts from Hückel (**28R(H)**) to Möbius (**28M(H)**) topology, a large increase of about 1,500 a.u. in the *β*_*HRS*_ value is observed. By changing the oxidation state while keeping constant the topology, and thus the ring strain, an increase of 2,347 a.u. in *β*_*HRS*_ is observed (**28R(H)** → **26R(H)**). The large changes in *β*_*HRS*_ demonstrate the potential of redox and topology interconversions in hexaphyrins as a novel type of optical switches. Overall, molecular symmetry, particularly the presence or absence of an inversion point, remains the most influencing property determining the *β*_*HRS*_, followed by the macrocyclic planarity and aromaticity.

The first hyperpolarizability contrast, defined as the ratio (definition 1, see Eq. 1) or the difference (definition 2, see Eq. 2) between the *β*_*HRS*_ of the ON state (the highest value of *β*_*HRS*_) and the OFF state (the lowest value of *β*_*HRS*_) involved in the switching, is the figure of merit for identifying efficient NLO switches. The larger is the *β*_*HRS*_ contrast, the more efficient becomes the switch. The values of the NLO contrasts for all possible interconversions can be found in Supplementary Tables S1, S2 and Supplementary Figure S3. As expected, when the OFF state is assigned to the centrosymmetric **28R(H)** structure, the highest NLO contrasts are found. Focusing on static *β*_*HRS*_ ratio-based contrast values, the redox switch **28R(H)** → **26R(H)** exhibits the largest contrast, followed by **28R(H)** → **26D(H)** and then the topological switch **28R(H)** → **28M(H)**, although all the contrasts are of the same order of magnitude (10^6^, since the *β*_*HRS*_ value of **28R** was set to 0.001 a.u.). The higher the variation of the symmetry and aromaticity between the OFF and ON states, the larger the NLO contrasts. The redox interconversion **28R(H)** → **26R(H)** yields higher NLO contrasts than the topological switch **28R(H)** → **28M(H)**, presumably due to larger variation in aromaticity involved in the former switch. Note that the Hückel configuration (**26R(H)**) is slightly more aromatic than the Möbius one (**28M(H)**) according to the majority of aromaticity descriptors (Table 1). When we have a look at the second contrast definition, the *β*_*HRS*_ difference, the most efficient NLO switches coincide with the ones found using the *β*_*HRS*_ ratio, with **28R(H)** as the OFF state. However, additional interconversions can be identified for which the contrast differs by less than an order of magnitude from the best performing switches. The ratio definition of the contrast motivates the selection of the centrosymmetric **28R** as the ultimate OFF state for NLO-type molecular switching applications, whereas for the difference definition more than one potential OFF-state candidate is put forward.

Finally, it is observed that the *β*_*HRS*_ values and the *β*_*HRS*_ contrasts are generally enhanced in the dynamic regime, indicating that the switch performance can be further fine-tuned with the wavelength of the incident light (see also Supplementary Figure S2). Remarkably, the values in Table 1 denote that the ON and OFF states of the **26D(H)**/**28M(H)** interconversion interchange when a frequency of 1.165 eV is applied for the first hyper-Rayleigh scattering in the dynamic regime, most clearly demonstrated by the opposite sign of the contrast value in Supplementary Table S2. A plausible explanation is that the **28M(H)** system is close to resonance. Generally, systems close to resonance are characterized by exalted magnitudes of *β*_*HRS*_ in the dynamic regime at certain wavelengths. Half of the Q-band’s excitation energy and half of the B-band’s excitation energy correspond to the first and the second resonance energy, respectively. In Supplementary Figure S1, the *β*_*HRS*_ is plotted against the incident energy of the photon for the different unsubstituted hexaphyrins. It is observed that, besides **28M(H)**, none of the systems are close to resonance in the dynamic regime.

### 4.2 Effect of the *Meso*-Substituent in the Hexaphyrin-Based Switches

**SCHEME 3:**
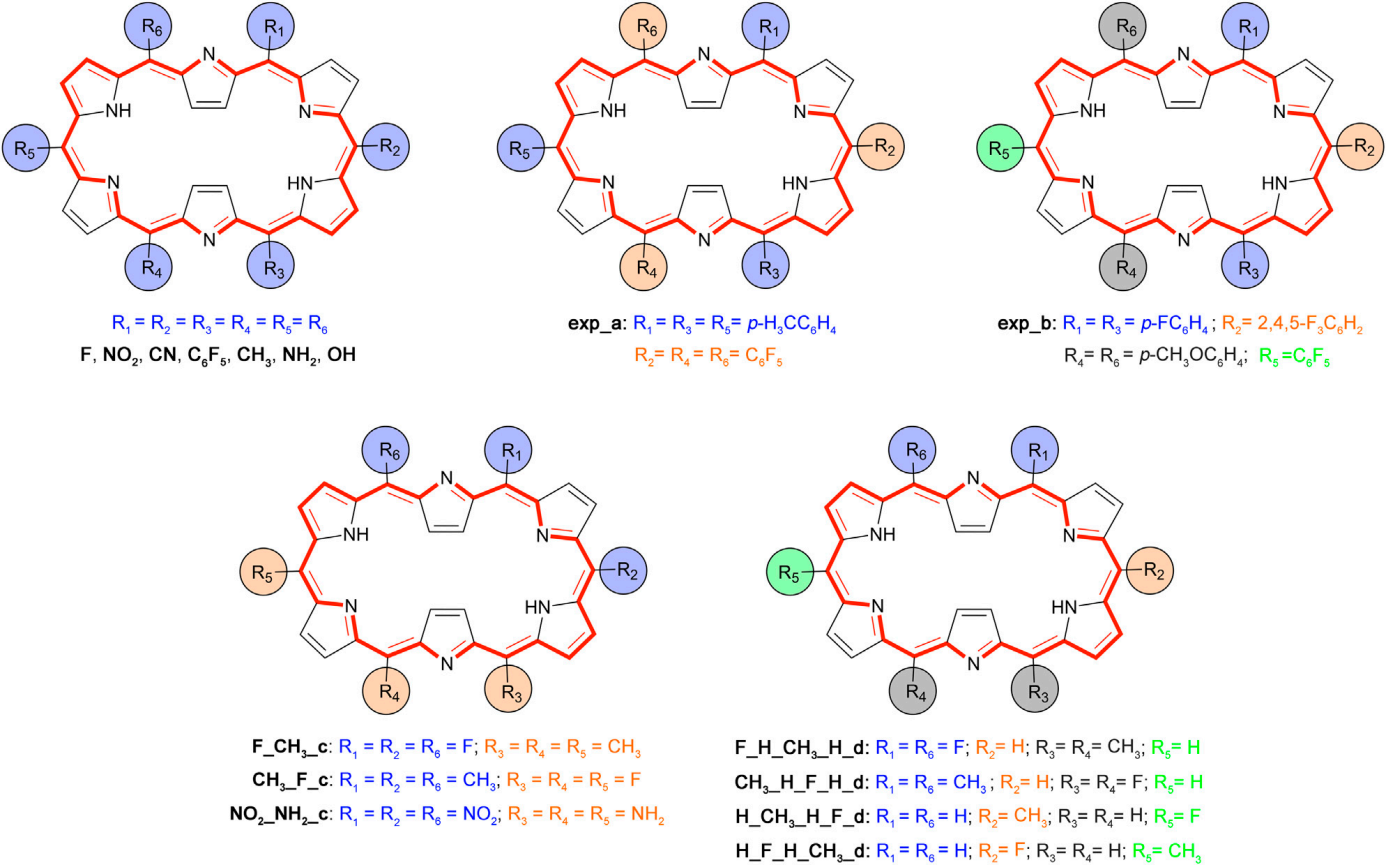
Overview and labelling of the *meso*-substituted structures based on the **26R** topology.

As a proof of concept, we concentrate on the effect of *meso*-substitution on the NLO responses of hexaphyrins, in particular, the *β*_*HRS*_. Since the **28R(H)** → **26R(H)** redox-based switch and the **28R(H)** → **28M(H)** topological interconversion showed high NLO contrasts, these two switches are selected for further investigation. We consider five distinct substitution patterns (Scheme 3), the first three based on currently synthesizable [26] and [28] hexaphyrins (Suzuki and Osuka, 2003; Plamont et al., 2017) and the final two taken from a previous computational study on efficient optical topological switches of [28]hexaphyrin (Torrent-Sucarrat et al., 2017b).

The experimental substitution patterns are based on synthetically available routes towards *meso*-substituted hexaphyrins (Tanaka and Osuka, 2016). At present, the A_3_B_3_-type [26]hexaphyrins (Scheme 3, **exp_a**) with a range of *meso*-aryl substituents can be synthesized via acid-catalysed condensation of *meso*-substituted dipyrromethene and aryl aldehydes (Suzuki and Osuka, 2003). More recently, a two-step synthesis of *meso*-substituted [28]hexaphyrins was discovered for the preparation of unprecedented A_2_BC_2_D-hexaphyrins (Scheme 3, **exp_b**) containing up to four different kinds of *meso*-appended aryl groups (Plamont et al., 2017). A variety of *meso*-substituents including electron-donating groups (EDG) and electron-withdrawing groups (EWG), inducing different electronic and steric effects, are considered. For each state of the molecular switch, the most relevant geometric, electronic, and optical properties are summarized in Table 2.

**TABLE 2:** Most relevant structural, energetic and optical properties of the *meso*-substituted hexaphyrins.

Substituents	26R				28R				28M				contrast (ratio)		contrast (difference)	
	***ϕ***_***p***_ ^a^	**Π** ^b^	**Δ**_***HL***_ ^c^	***β***_***HRS***_ ^d^	***ϕ***_***p***_ ^a^	**Π** ^a^	**Δ**_***HL***_ ^c^	***β***_***HRS***_ ^d^	***ϕ***_***p***_ ^a^	**Π** ^a^	**Δ**_***HL***_ ^c^	***β***_***HRS***_ ^d^	**26R**/**28R**	**28M**/**28R**	**26R**–**28R**	**28M**-**28R**
**-H**	11.66	0.88	3.66	2.35×10^3^	10.87	0.90	3.00	∼0^e^	31.54	−0.45	3.82	1.47×10^3^	2.35×10^6^	1.47×10^6^	2.35×10^3^	1.47×10^3^
**-F**	8.83	0.90	3.23	8.62×10^3^	11.01	0.90	3.11	∼0^e^	32.55	−0.46	3.42	2.09×10^3^	8.62×10^6^	2.09×10^6^	8.62×10^3^	2.09×10^3^
**-CN**	12.69	0.88	3.63	1.64×10^3^	27.98	0.54	3.05	1.17×10^3^	32.38	−0.46	3.63	1.54×10^3^	1.40×10^0^	1.31×10^0^	4.65×10^2^	3.68×10^2^
**-** **NO**_**2**_	15.61	0.79	3.83	1.46×10^3^	40.58	0.18	3.66	1.02×10^3^	34.20	−0.43	3.64	2.28×10^3^	1.44×10^0^	2.23×10^0^	4.44×10^2^	1.26×10^3^
**-** **PhF**_**5**_	16.31	0.80	3.64	3.18×10^3^	47.95	0.47	3.28	1.86×10^3^	32.91	−0.38	3.68	1.75×10^3^	1.71×10^0^	9.41×10^−1^	1.32×10^3^	-1.09×10^2^
**-** **CH**_**3**_	18.11	0.66	1.57	2.96×10^4^	21.91	0.68	3.18	∼0^e^	32.59	−0.46	3.68	1.16×10^3^	2.96×10^7^	1.16×10^6^	2.96×10^4^	1.16×10^3^
**-** **NH**_**2**_	29.26	0.53	2.52	3.37×10^4^	30.71	0.43	3.33	2.70×10^3^	33.23	−0.45	3.62	2.21×10^3^	1.25×10^1^	8.19×10^−1^	3.10×10^4^	-4.90×10^2^
**-OH**	14.28	0.86	2.56	2.57×10^4^	14.26	0.80	3.08	∼0^e^	32.52	−0.45	3.29	2.89×10^3^	2.57×10^7^	2.89×10^6^	2.57×10^4^	2.89×10^3^

**Patterns**	**26R**				**28R**				**28M**				**contrast (ratio)**		**contrast (difference)**	
	***ϕ***_***p***_ ^a^	**Π** ^b^	**Δ**_***HL***_ ^c^	***β***_***HRS***_ ^d^	***ϕ***_***p***_ ^a^	**Π** ^a^	**Δ**_***HL***_^*c*^	***β***_***HRS***_ ^d^	***ϕ***_***p***_ ^a^	**Π** ^a^	**Δ**_***HL***_ ^c^	***β***_***HRS***_ ^d^	**26R**/**28R**	**28M**/**28R**	**26R**–**28R**	**28M**-**28R**
**exp_a**	15.13	0.84	3.44	5.92×10^3^	19.07	0.68	2.93	3.16×10^3^	32.14	−0.43	3.42	3.59×10^3^	1.88×10^0^	1.14×10^0^	2.76×10^3^	4.37×10^2^
**exp_b**	20.76	0.70	3.39	8.36×10^3^	20.22	0.68	2.89	3.19×10^3^	31.90	−0.44	3.70	2.02×10^3^	2.62×10^0^	6.32×10^−1^	5.17×10^3^	−1.17×10^3^
**F_** **CH**_**3**_ **_** **c**	16.54	0.82	3.36	7.18×10^3^	24.30	0.55	3.32	1.75×10^3^	31.90	−0.47	3.58	2.07×10^3^	4.09×10^0^	1.18×10^0^	5.42×10^3^	3.15×10^2^
**CH**_**3**_ **_** **F** **_** **c**	17.69	0.76	3.46	6.23×10^3^	24.31	0.55	3.32	1.75×10^3^	32.41	−0.46	3.69	1.24×10^3^	3.55×10^0^	7.05×10^−1^	4.47×10^3^	-5.18×10^2^
**NO**_**2**_ **_NH**_**2**_ **_c**	30.76	0.48	3.62	6.37×10^3^	38.69	0.15	2.92	9.21×10^3^	34.64	−0.39	3.20	4.51×10^3^	6.92×10^−1^	4.90×10^−1^	-2.84×10^3^	-4.69×10^3^
**F_H_CH**_**3**_ **_H_d**	14.32	0.86	3.41	5.71×10^3^	20.37	0.65	3.14	1.51×10^3^	31.58	−0.47	3.84	1.62×10^3^	3.78×10^0^	1.07×10^0^	4.20×10^3^	1.08×10^2^
**CH**_**3**_ **_H_F_H_d**	15.46	0.82	3.49	5.55×10^3^	20.37	0.66	3.14	1.51×10^3^	32.77	−0.45	3.86	6.24×10^2^	3.68×10^0^	4.15×10^−1^	4.04×10^3^	-8.82×10^2^
**H_CH**_**3**_ **_H_F_d**	12.08	0.89	3.61	3.07×10^3^	11.37	0.89	3.11	2.20×10^2^	31.84	−0.43	3.48	1.45×10^3^	1.40×10^1^	6.61×10^0^	2.85×10^3^	1.23×10^3^
**H_F_H_CH**_**3**_ **_d**	11.88	0.90	3.60	3.68×10^3^	11.37	0.89	3.11	2.20×10^2^	32.23	−0.44	3.79	1.89×10^3^	1.67×10^1^	8.58×10^0^	3.46×10^3^	1.67×10^0^

a Ring strain (ϕ_p_ in °).

b Torsional *π*-conjugation index (*Π*).

c HOMO-LUMO gap (*Δ*_HL_ in eV) computed at the CAM-B3LYP/6-311+G(d,p) level of theory.

d Static β_HRS_ (in a.u.) in gas phase computed at CAM-B3LYP/6-311+G(d,p) level of theory. The contrast (ratio) of the substitution patterns that show an OFF state (**28R**) with a *β*_*HRS*_ of ∼0 a.u. are computed considering a value of 0.001 a.u. for their OFF state.

e *β*_*HRS*_ of the centrosymmetric **28R(H)** is not zero due to round-off errors in the coordinates.

Depending on the bulkiness of the substituent and the topology of the *π*-system, the introduction of *meso*-substituents distorts the macrocyclic planarity in different degrees. In the **26R** structures bearing the same substituent, all substituents except F reduce the planarity of the macrocycle, thus decreasing the effective *π*-overlap, as shown by the reduced values of the Π descriptor. This effect is particularly large for the CH_3_ and NH_2_ substituents, in which *ϕ*_*p*_ increases by 6.5° and 17.6° and Π decreases by 0.22 and 0.35, respectively. The planarity distortion increases considerably the *β*_*HRS*_ response of the **26R** state, up to an order of magnitude. In addition, the electronic nature of the *meso*-substituent plays an important role with EDGs leading to larger *β*_*HRS*_ responses than EWGs. It is interesting to further note that the presence of EDGs significantly reduces the Δ_*HL*_ of the **26R** structures, while this effect is less pronounced for EWGs. Accordingly, lowering the Δ_*HL*_ results in higher first hyperpolarizabilities and other NLO properties in line with previous studies (Suslick et al., 1992; Yoon et al., 2008).

In the case of the **28R** structures, the symmetry of the conformation is a key factor in tuning the NLO response. Either a centrosymmetric *meso*-substituted **28R** is found, or a more stable *C*_2_ or *C*_1_-symmetric structure. Recall that centrosymmetric structures produce null *β*_*HRS*_ responses. The introduction of several *meso*-substituents such as NO_2_, CN and PhF_5_ breaks the centrosymmetry to either a *C*_1_ or *C*_2_ symmetry by disturbing the planarity of the system. The more pronounced becomes the geometrical distortion (higher *ϕ*_*p*_, lower Π), the higher becomes the NLO response of the **28R** state. For example, the NO_2_ substituent significantly distorts the macrocyclic planarity making the *π*-conjugation rather ineffective with a concomitant huge increase in the *β*_*HRS*_ response of **28R(** **NO**_**2**_ **)**.

A change in topology from Hückel to Möbius also affects the *β*_*HRS*_ response of [28]hexaphyrins. The singly-twisted **28M** conformation allows for a better arrangement of the *meso*-substituents and, consequently, *meso*-substitution does not induce a significant change in the torsional or structural descriptors. Compared to their Hückel **28R** counterparts, most *meso*-substituted Möbius structures show a (much) larger *β*_*HRS*_ response of the order of 10^3^ and this response can be tuned by the inclusion of electron-withdrawing and electron-donating groups, however without any clear trend.

Several push-pull substitution patterns were also investigated to assess the influence of the number and position of the substituents. Similar conclusions can be drawn regarding the structural parameters, *i.e.,* slight to moderate increase and decrease for their *ϕ*_*p*_ and Π values, respectively. The tabulated *meso*-substituted structures taken from literature lend themselves to several interesting observations. First, the two synthetically available substitution patterns **exp** **_** **a** (A_3_B_3_) and **exp** **_** **b** (A_2_BC_2_D), have significantly enhanced *β*_*HRS*_ responses with respect to the unsubstituted structures, especially the **28R** conformation. We also point out that the **26R(exp** **_** **a)** and **26R(exp** **_** **b)** are outperformed by several of the previously discussed *meso*-substituted structures leaving room for improvement in terms of the NLO response. Second, we have computed some of the push-pull structures suggested by Torrent-Sucarrat et al. (2017b), which can be compartmentalized into two distinct substitution patterns, indicated in their name by **_c** and **_d**. Structures **F_CH**_**3**_ **_c** and **CH**_**3**_ **_F_c** patterns are very similar, but the substituents are differently positioned on the macrocycle. Nonetheless the **F_CH**_**3**_ **_c** shows a higher NLO response for **26R** and **28M** than its **CH**_**3**_ **_F_c** counterpart. Intensifying the electronic character of this pattern by replacing the CH_3_ and F by NH_2_ and NO_2_, respectively, results in more distorted structures for all hexaphyrins. For **28R(** **NO**_**2**_ **_NH**_**2**_ **_c)** and **28M(** **NO**_**2**_ **_NH**_**2**_ **_c)**, this leads to significantly larger *β*_*HRS*_ values compared to the **F_CH**_**3**_ **_c** equivalents. However, this geometrical distortion does not necessarily result in a higher NLO response as observed for **26R(** **NO**_**2**_ **_NH**_**2**_ **_c)**. Lastly, the number and orientation of the substituents also influence the NLO response. For substitution pattern **d**, we compare disubstituted with quadruply substituted hexaphyrins, again introducing push-pull functionalization via fluoro and methyl groups. Putting the CH_3_ or F at the R_1_/R_6_ and R_3_/R_4_ results in higher NLO responses for the **26R** and **28R** conformation with a lower planarity and higher ring strain with respect to the substitution of the R_2_/R_5_. This demonstrates that the number of *meso*-substitutions can be a determining factor in magnifying the *β*_*HRS*_ response. In addition, the orientation of the push-pull components has a clear effect, though to a lesser degree. Conversely, the **28M** conformation shows more similar NLO responses for the different positions, with the exception of **28M(** **CH**_**3**_ **_H_F_H_d)**.

From this table, it becomes clear that EDGs (CH_3_, NH_2_, OH) give the highest *β*_*HRS*_ values for the **26R** state and that push-pull substitution is not essential for this configuration. Conversely, the *β*_*HRS*_ response of the **28M** conformations varies to a much smaller extent upon *meso*-substitution as compared to the Hückel topologies. The most pronounced changes in the NLO response, however, are observed for the **28R** state, where the structural symmetry, more precisely the absence of presence of an inversion point, dominates the effect of the chemical modifications on the *β*_*HRS*_ response. As the lowest *β*_*HRS*_ responses usually correspond to the **28R** structures, they are selected as the OFF state in the evaluation of the switch contrast. Both the ratio and the difference based definitions are considered. We observe that the presence of a centrosymmetric OFF state influences the ratio based contrast immensely yielding contrasts up to 10^6^-10^7^. In absence of a centrosymmetric OFF state, push-pull patterns compete with fully EDG substituted **28R** → **26R** switches. In line with the parent structures, the redox switch outperforms the topology switch upon *meso*-substitution. Moreover, for several topology switches a reversal of the ON and OFF state has occurred, as witnessed from the ratio values below 1 and the negative contrast differences. Inclusion of push-pull functional groups can play a major role in improving the switching efficiency of **28R** → **28M** but the positioning of these groups is of vital importance.

**FIGURE 5:**
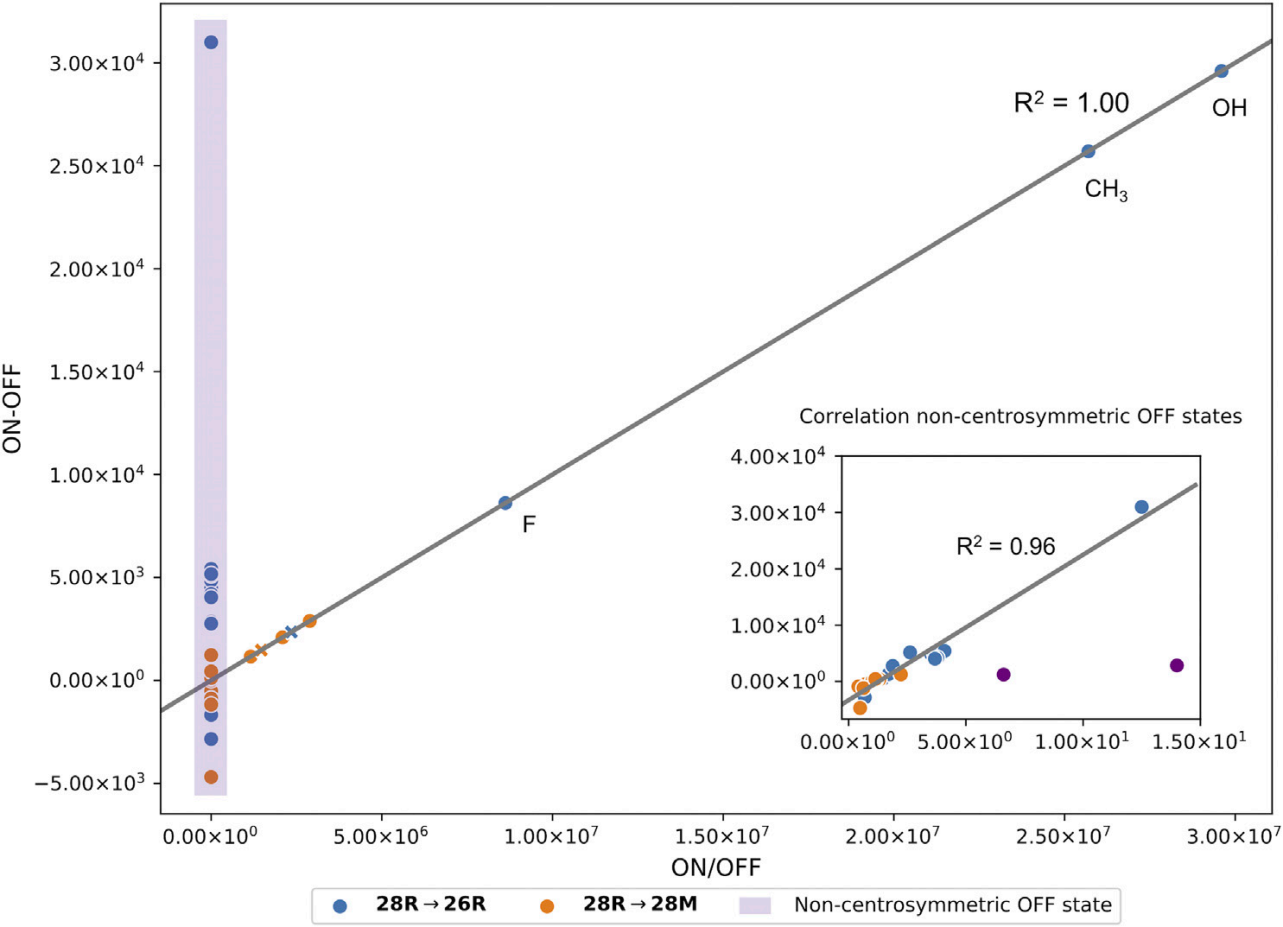
Correlation plot between the two contrast definitions based on the ratio and the difference of the *β*_*HRS*_ responses of all meso-substituted structures with a centrosymmetric OFF state (*R* ^2^ of 1.00). The datapoints marked with an X are the unsubstituted hexaphyrins. All switches with a non-centrosymmetric OFF state are highlighted in the purple box. A separate plot in the right corner shows the correlation of these systems (*R* ^2^ of 0.96).

We finally would like to note that overall both contrast definitions correlate well. For centrosymmetric OFF states, the ratio and the difference are linearly dependent (Figure 5). As mentioned before, these coincide with a high and low NLO response for the ON and OFF state, respectively. Most switches with a non-centrosymmetric OFF state display a relatively high NLO response and are therefore characterized by a low ratio-based contrast between 0.69 and 14, but a high difference-based contrast (see purple box in Figure 5). Upon removal of 2 outliers (**28R(** **H_CH**_**3**_ **_H_F_d)** → **26R(** **H_CH**_**3**_ **_H_F_d)** and **28R(** **H_CH**_**3**_ **_H_F_d)** → **28M(** **H_CH**_**3**_ **_H_F_d)**, a strong linear correlation between the two contrast definitions is found with a correlation coefficient (*R* ^2^) of 0.96. We therefore expect similar results when both definitions are optimized for substitution patterns for which centrosymmetric OFF states are not accessible.

### 4.3 Inverse Design of Hexaphyrin-Based Optical Switches

#### 4.3.1 Prestudy

Before we start our inverse design optimizations, a small prestudy is conducted in which the most favorable functional groups are determined when substituting each pair of *meso*-positions facing each other diagonally on the macrocycle (R_1_/R_4_, R_2_/R_5_ and R_3_/R_6_). For each site combination, the *β*_*HRS*_ responses of the **26R**, **28R** and **28M** conformations and the resulting contrasts of both switches are summarized in Table 3. This prestudy is of particular interest since we observed that the most stable^1^ **28R** conformation of some of the fully substituted hexaphyrins has *C*_*i*_ symmetry, largely impacting the *β*_*HRS*_ contrasts of both the topological and redox switches.

**TABLE 3:** The *β*_*HRS*_ response^a^ of *meso*-substituted hexaphyrins’ individual sites (R_1_/R_4_, R_2_/R_5_ and R_3_/R_6_) and their corresponding contrasts using both the ratio and difference definition.

Substituent	26R			28R			28M		
	**R**_**1**_ **/** **R**_**4**_	**R**_**2**_ **/** **R**_**5**_	**R**_**3**_ **/** **R**_**6**_	**R**_**1**_ **/** **R**_**4**_	**R**_**2**_ **/** **R**_**5**_	**R**_**3**_ **/** **R**_**6**_	**R**_**1**_ **/** **R**_**4**_	**R**_**2**_ **/** **R**_**5**_	**R**_**3**_ **/** **R**_**6**_
F	5.06×10^3^	2.95×10^3^	4.63×10^3^	∼0^b^	∼0^b^	∼0^b^	3.54×10^2^	1.70×10^3^	1.17×10^3^
CN	1.20×10^3^	6.38×10^3^	2.45×10^3^	∼0	∼0^b^	∼0^b^	1.96×10^3^	1.53×10^3^	5.54×10^2^
NO_2_	1.14×10^3^	4.14×10^3^	1.52×10^3^	1.34×10^3^	∼0^b^	9.23×10^2^	2.90×10^3^	2.55×10^3^	2.01×10^3^
CH_3_	3.21×10^3^	3.87×10^3^	3.46×10^3^	∼0^b^	∼0^b^	∼0^b^	4.58×10^2^	4.22×10^2^	9.00×10^2^
OH	9.99×10^3^	3.61×10^3^	8.92×10^3^	∼0^b^	∼0^b^	∼0^b^	8.95×10^2^	1.61×10^3^	2.13×10^3^
NH_2_	1.56×10^4^	2.74×10^3^	1.14×10^4^	∼0^b^	∼0^b^	1.28×10^3^ Â	9.63×10^2^	2.05×10^3^	1.36×10^3^

**Substituent**	**26R**/**28R**						**28M**/**28R**		
	**R**_**1**_ **/** **R**_**4**_	**R**_**2**_ **/** **R**_**5**_	**R**_**3**_ **/** **R**_**6**_				**R**_**1**_ **/** **R**_**4**_	**R**_**2**_ **/** **R**_**5**_	**R**_**3**_ **/** **R**_**6**_
F	5.06×10^6^	2.95×10^6^	4.63×10^6^				3.54×10^5^	1.70×10^6^	1.17×10^6^
CN	1.20×10^6^	6.38×10^6^	2.45×10^6^				1.96×10^6^	1.53×10^6^	5.54×10^6^
NO_2_	8.49×10^−1^	4.14×10^6^	1.64×10^1^				2.16×10^0^	2.55×10^6^	2.18×10^0^
CH_3_	3.21×10^6^	3.87×10^6^	3.46×10^6^				4.58×10^5^	4.22×10^5^	9.00×10^5^
OH	9.99×10^6^	3.61×10^6^	8.92×10^6^				8.95×10^5^	1.61×10^6^	2.13×10^6^
NH_2_	1.56×10^7^	2.74×10^6^	8.09				9.63×10^5^	2.05×10^6^	1.47×10^0^

**Substituent**	**26R**–**28R**						**28M** -**28R**		
	**R**_**1**_ **/** **R**_**4**_	**R**_**2**_ **/** **R**_**5**_	**R**_**3**_ **/** **R**_**6**_				**R**_**1**_ **/** **R**_**4**_	**R**_**2**_ **/** **R**_**5**_	**R**_**3**_ **/** **R**_**6**_
F	5.06×10^3^	2.95×10^3^	4.63×10^3^				3.54×10^2^	1.70×10^3^	1.17×10^3^
CN	1.20×10^3^	6.38×10^3^	2.45×10^3^				1.96×10^3^	1.53×10^3^	5.54×10^2^
NO_2_	-2.03×10^2^	4.14×10^3^	1.64×10^0^				1.56×10^3^	2.55×10^3^	1.64×10^0^
CH_3_	3.21×10^3^	3.87×10^3^	3.46×10^3^				4.57×10^2^	4.22×10^2^	8.97×10^2^
OH	9.99×10^3^	3.61×10^3^	8.92×10^3^				8.95×10^2^	1.61×10^3^	2.13×10^3^
NH_2_	1.56×10^4^	2.74×10^3^	8.91×10^0^				9.63×10^2^	2.05×10^3^	8.91×10^0^

a Static β_HRS_ (in a.u.) in gas phase computed at CAM-B3LYP/6-311+G(d,p) level of theory. The contrast (ratio) of the substitution patterns that show an OFF state (**28R**) with a β_HRS_ of ∼0 a.u. are computed considering a value of 0.001 a.u. for their OFF state.

b β_HRS_ of the centrosymmetric **28R** is not zero due to round-off errors in the coordinates.

For the **26R** conformation, the highest responses are retrieved for EDGs (NH_2_ and OH) substituted on the R_1_/R_4_ and R_3_/R_6_ site combinations and EWGs (CN and NO_2_) on the R_2_/R_5_ positions. Substituents with an opposite electronic character on the aforementioned site combinations exhibit two to even thirteen times lower *β*_*HRS*_ responses. The NH_2_ functional group outperforms other substituents from 30% to around an order of magnitude, except for the R_2_/R_5_ positions where it shows the lowest HRS response of all. A different trend is obtained for the **28M** conformation, where only EWGs (CN and NO_2_) on R_1_/R_4_ reveal a high *β*_*HRS*_ response with respect to the other substituents, while on other positions both EWGs and EDGs (NO_2_, NH_2_ and OH) perform about equally well. Note that for both the **26R** and **28M** conformations, the greatest impact can be achieved by substituting sites R_1_/R_4_, however, where for **26R** an almost sevenfold enhancement in *β*_*HRS*_ can be gained, functionalization of **28M** induces only a twofold improvement compared to the parent conformation. Finally, **28R**, typically the OFF state, shows for all substituents a nearly zero *β*_*HRS*_ response due to its centrosymmetry. The only exceptions are the NO_2_ substituents positioned on the R_1_/R_4_ and R_3_/R_6_ site combinations and the NH_2_ substituted R_3_/R_6_ conformation, which all have *C*_2_ symmetry. Because most functionalized **28R** structures have a very low NLO response, the discussed trends apply to both the ratio-based and difference-based contrasts of the associated redox (**28R** → **26R**) and topological switches (**28R** → **28M**).

**SCHEME 4:**
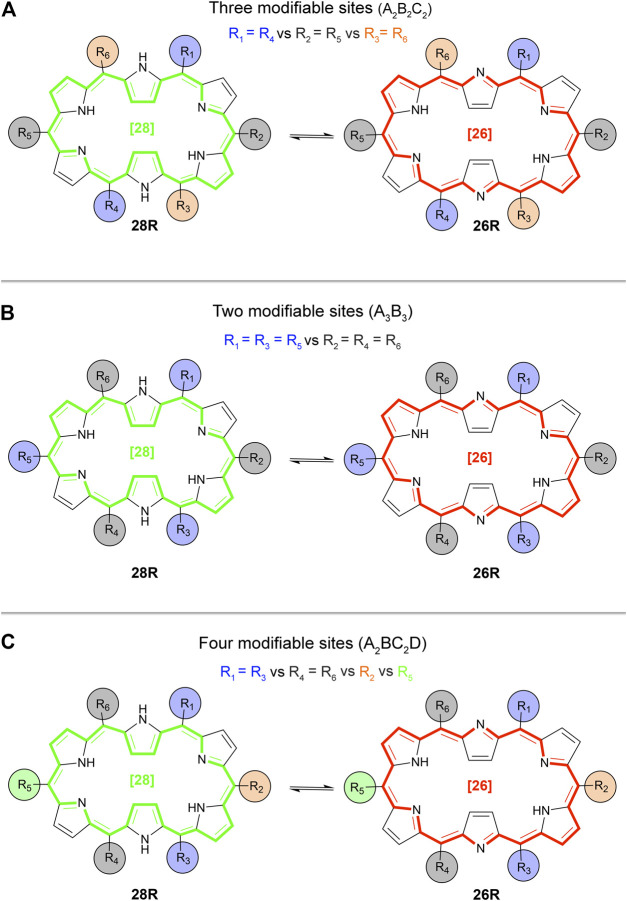
Overview of the studied substitution patterns: **(A)** A_2_B_2_C_2_ type hexaphyrins, **(B)** A_3_B_3_ type hexaphyrins and **(C)** A_2_BC_2_D type hexaphyrins. Example given for the redox switch **28R** → **26R**.

Building on our previous proof of concept and the additional disubstitutions, we expand our search for well-performing functionalized redox and topological switches, *i.e.* connected to a high NLO contrast, by means of the BFS algorithm. Three sets of BFS runs are executed considering three substitution patterns and a fragment library containing seven substituents (-NH_2_ (amino-), -OH (hydroxyl-), -CH_3_ (methyl-), -H, -F (fluoro-), -CN (cyano-) and -NO_2_ (nitro-)). We introduce a new substitution pattern, called A_2_B_2_C_2_ (Scheme 4A). It considers all *meso*-positions, but fixes the same substituent on the diagonal sites (R_1_ = R_4_ versus R_2_ = R_5_ versus R_3_ = R_6_). The other examined substitution patterns are the two synthesizable ones as described in the proof of concept section, indicated as A_3_B_3_ (Scheme 4B) and A_2_BC_2_D (Scheme 4C) containing up to two and four, respectively, different kinds of functional groups (Plamont et al., 2017). In the next part, we discuss a series of *β*_*HRS*_ contrast maximizations to identify the most optimal functionalizations of the three substitution patterns showing enhanced NLO contrasts.

#### 4.3.2 Redox Switches

**FIGURE 6:**
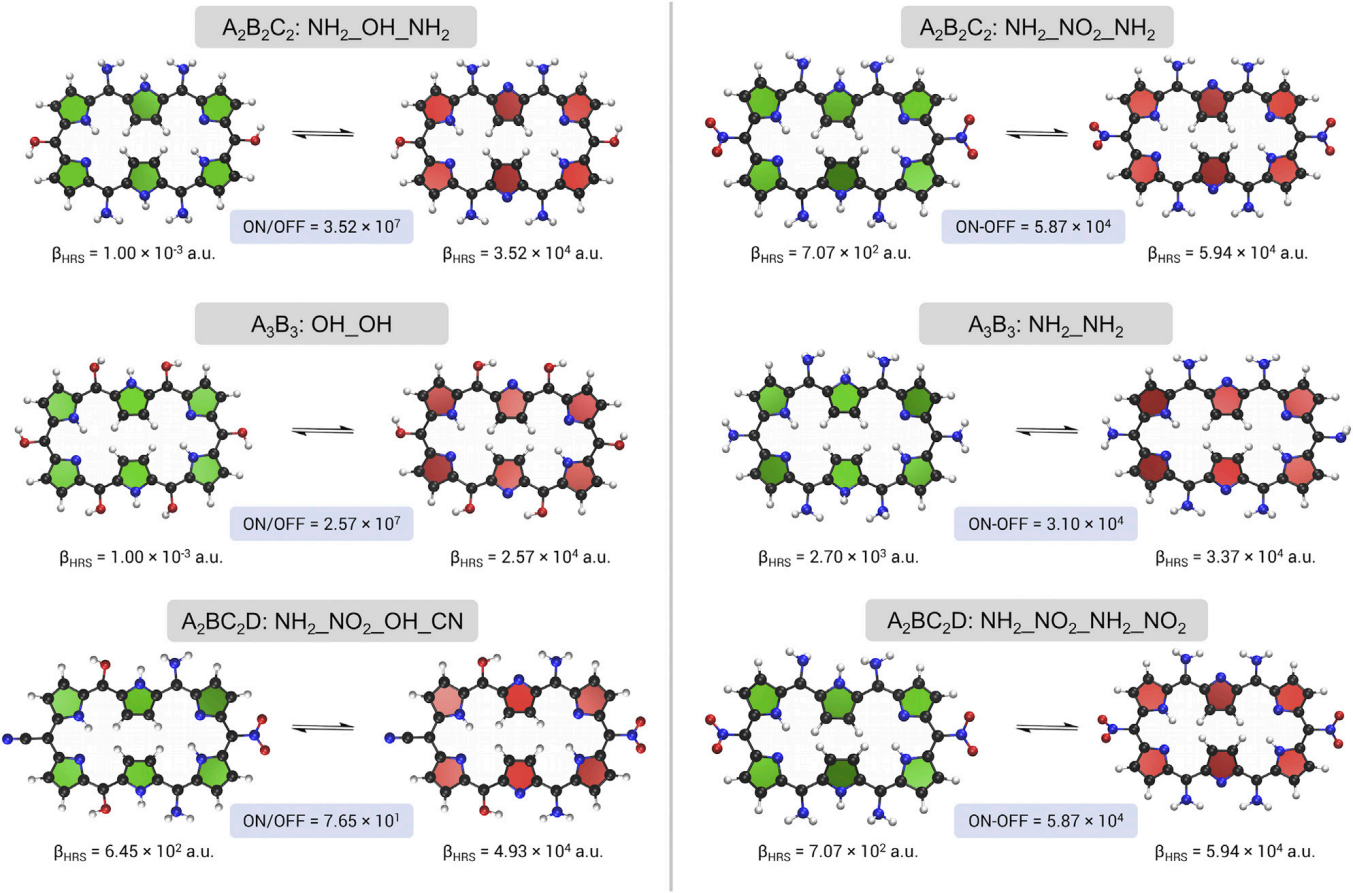
A schematic overview of the redox switches with the highest NLO contrast, *β*_*HRS*_ ratio on the left and *β*_*HRS*_ difference on the right, upon *meso*-substitution for the three substitution patterns.

The *meso*-substituted redox switches with the highest *β*_*HRS*_ ratio and difference obtained through the BFS algorithm are summarized for all three substitution patterns in Figure 6. In short, the best performing macrocycles either involve only EDGs (NH_2_ and OH) or a combination of 4 EDGs (NH_2_ and OH) and 2 EWGs (NO_2_ and CN), with the EWGs placed on sites R_2_/R_5_. In the following paragraphs, a more detailed analysis of the BFS results is provided.

The optimum molecular switches for the first substitution pattern A_2_B_2_C_2_ contain 4 amino groups, located at sites R_1_/R_4_ and R_3_/R_6_, and have either an EDG (OH) or EWG (NO_2_) positioned on R_2_/R_5_, with the former obtained for the ratio and the latter for the difference contrast maximization. Both switches are encountered during both BFS optimizations. The choice of one or the other is directly related to the contrast definitions. On the one hand, we have the ON state of the most optimal switch retrieved from the *β*_*HRS*_ difference maximization, which leads to a more enhanced NLO response in comparison to that of the *β*_*HRS*_ ratio optimization (*β*_*HRS*_(**26R(** **NH**_**2**_ **_NO**_**2**_ **_NH**_**2**_ **)**) = 5.94 ×10^4^ a.u. versus *β*_*HRS*_(**26R(** **NH**_**2**_ **_OH_NH**_**2**_ **)**) = 3.52 ×10^4^ a.u.), for which a push-pull configuration is responsible. On the other hand, there is the magnitude of the OFF state’s NLO response, which becomes more dominant when optimizing the ratio compared to the difference, especially when the OFF state has a near-zero *β*_*HRS*_ value due to centrosymmetry (*β*_*HRS*_(**28R(** **NH**_**2**_ **_OH_NH**_**2**_ **)**) = ∼0 a.u. versus *β*_*HRS*_(**28R(** **NH**_**2**_ **_NO**_**2**_ **_NH**_**2**_ **)**) = 7.07 ×10^2^ a.u.) inherently amplifying the ratio contrast. Surprisingly, the push-pull configuration (**NH**_**2**_ **_CN_NH**_**2**_), conjectured based on our pairwise prestudy, was not selected as the optimum, despite having a centrosymmetric OFF state. The cyano groups on sites R_2_/R_5_ underperform, with a *β*_*HRS*_ value even lower than for the unsubstituted R_2_ and R_5_ positions . This low value can probably be traced back to the structural parameters for this macrocycle. The **26R(** **NH**_**2**_ **_OH_NH**_**2**_ **)** conformation has a higher ring strain and lower planarity (*ϕ*_*p*_: 13.60° and Π: 0.77) than the predicted **26R(** **NH**_**2**_ **_CN_NH**_**2**_) conformation (*ϕ*_*p*_: 12.22° and Π: 0.80). Compared to our parent switch, the switching efficiency increases by a factor of 15–25 (depending on which contrast definition is used), with site combination R_1_/R_4_ being the most and R_2_/R_5_ the least influential *meso*-positions, in line with the findings of the pairwise disubstitution study.

Optimization of the second substitution pattern A_3_B_3_ results for both definitions in a very similar structure. All positions are substituted with the same EDG (OH for the contrast and NH_2_ for the difference). Whereas for the ratio optimum a centrosymmetric OFF state is acquired, **28R(** **NH**_**2**_ **_** **NH**_**2**_ **)** has *C*_2_ symmetry with a *β*_*HRS*_ value of the order of 10^3^. These *meso*-substituted structures were already designated in our proof of concept as high-potential switches, although it was shown that the sixfold methyl-substituted hexaphyrin performs even better than the OH-substituted one. Nonetheless, the A_3_B_3_ pattern does not achieve the same efficiency as the A_2_B_2_C_2_ pattern with a 27% lower ratio for **OH_OH** and a 47% lower difference for **NH**_**2**_ **_NH**_**2**_ compared to the A_2_B_2_C_2_ optima. The best performing push-pull switch in the A_3_B_3_ setting, according to the difference-based contrast, is **28R(** **F_NH**_**2**_ **)** → **26R(** **F_NH**_**2**_ **)**, having a NLO contrast of approximately two third of the BFS optimum and 7 times larger than the currently synthesizable **exp_a** (*β*_*HRS*_ difference = 2.76 × 10^3^ a.u.).

Finally, the A_2_BC_2_D pattern optimizations using both contrast definitions are discussed. Two different optima are obtained, but both contain a combination of EWGs and EDGs. The best redox switch of the ratio optimization is **28R(** **NH**_**2**_ **_NO**_**2**_ **_OH_CN)** → **26R(** **NH**_**2**_ **_NO**_**2**_ **_OH_CN)** and contains 4 distinct functional groups. The switch combines a *C*_1_-symmetric OFF state with a relatively low response of the order of 10^2^ with a significantly enhanced response of the ON state. On the R_2_ and R_5_ sites two different EWGs (CN and NO_2_) are positioned while for the other sites (R_1_, R_3_, R_4_ and R_6_) EDGs (OH and NH_2_) are preferred. The BFS optimum for the difference maximization is macrocycle **NH**_**2**_ **_NO**_**2**_ **_NH**_**2**_ **_NO**_**2**_, which is the same optimum as found for the A_2_B_2_C_2_ pattern. The difference based contrast is more than 10 times larger than **exp_b** (5.17 × 10^3^). In fact, its **26R** structure has the highest NLO response of all visited redox switches. Hence, combining strongly EDGs with strongly EWGs, with a preference for more EDGs, is a good recipe to magnify the switching efficiency of hexaphyrin-based redox switches, which is in agreement with the findings in our proof of concept. Nonetheless, the OFF state’s symmetry remains a key player for improving the contrast when the original contrast definition is employed.

#### 4.3.3 Topological Switch

**FIGURE 7:**
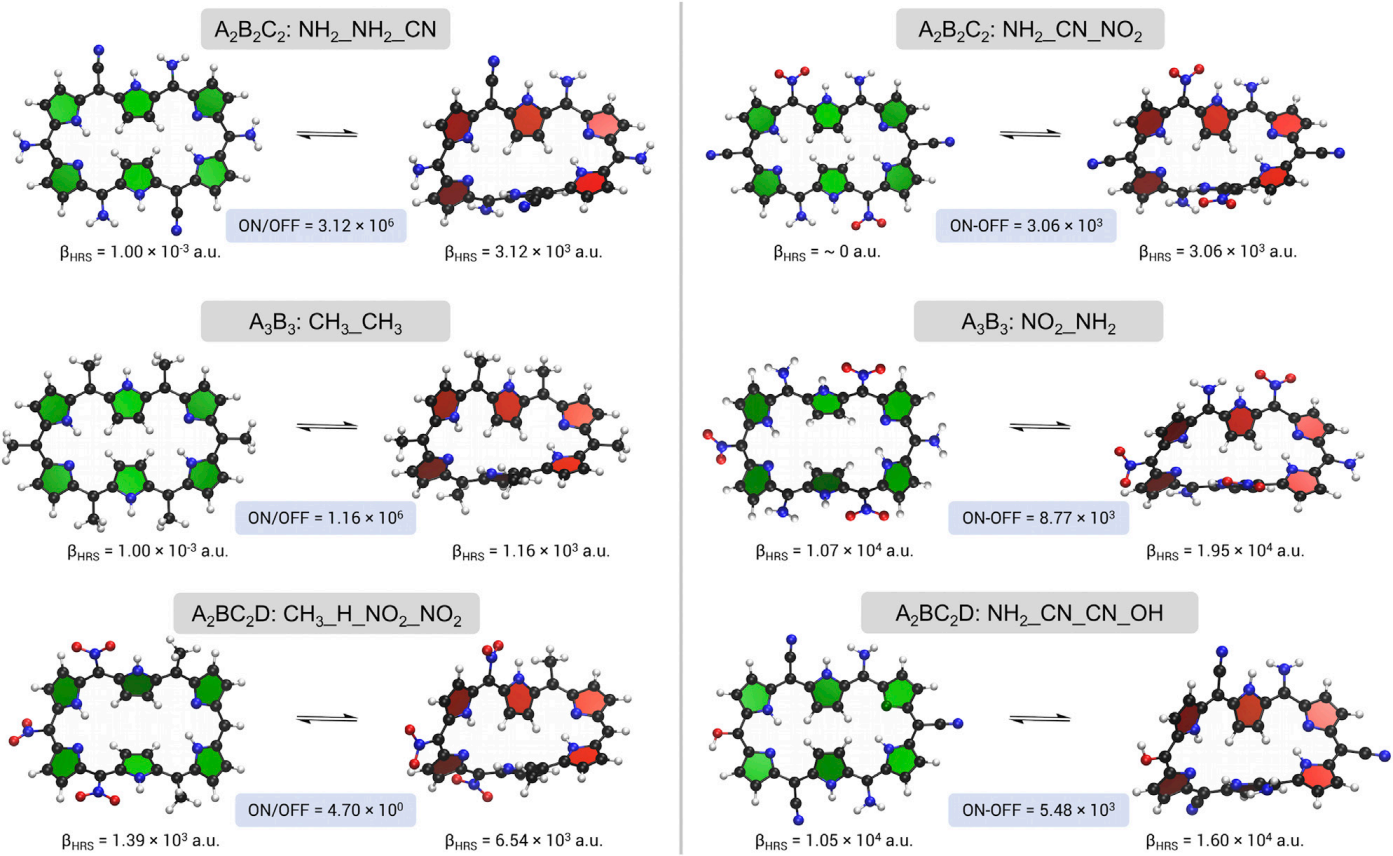
A schematic overview of the topological switches with the highest NLO contrast, *β*_*HRS*_ ratio on the left and *β*_*HRS*_ difference on the right, upon *meso*-substitution for the three substitution patterns.

Figure 7 gives an overview of all optimal topological switches obtained from six BFS runs with the ratio or difference in *β*_*HRS*_ as the target function. Nearly all optima contain combinations of EWGs and EDGs with various electronic characters (inductive versus mesomeric). Again, the centrosymmmetry of the OFF state plays a major role in maximizing the NLO response ratio, but to a lesser extent than for the difference. On the other hand, much higher *β*_*HRS*_ responses are found for the **28M** and **28R** states of the difference optima, compared to the best ratio switches. In general, the NLO contrasts are lower in value than the redox switches. In the following paragraphs, the contrast maximizations are briefly discussed.

The two figure of merit maximizations of the A_2_B_2_C_2_ pattern result in relatively different optima. The topological switch for the ratio maximization contains 4 mesomerically EDGs (NH_2_), on sites R_1_/R_4_ and R_2_/R_5_, and 2 EWGs (CN) positioned on R_3_/R_6_: **28R(** **NH**_**2**_ **_NH**_**2**_ **_CN)** → **28M(** **NH**_**2**_ **_NH**_**2**_ **_CN)**. In contrast, the EWGs outnumber the EDGs in the difference optimum, with amino groups placed on R_1_/R_4_, cyano groups on R_2_/R_5_ and nitro groups on R_3_/R_6_. Note, however, that the ratio optimum is performing slightly better than the difference optimum, also when the difference-based contrast is considered (3.12 × 10^3^ versus 3.06 × 10^3^ a.u.). Once again, the OFF states are centrosymmetric structures with zero HRS responses, whereas the ON states have improved NLO responses, among the largest we have encountered so far for the **28R** → **28M** topological switch. Surprisingly, none of the functional groups emerge from our two-site prestudy as one of the best performing substituents on their respective positions in the ratio and difference optima, which is particularly the case for CN on sites R_2_/R_5_ or R_3_/R_6_. The synergetic effect between the different functionalizations may be attributed to the formation of a push-pull configuration. Overall, this substitution pattern leads to a twofold enhancement with respect to the parent switch and even more compared to the synthesized macrocycles **exp_a** and **exp_b**.

The contrast optimizations for the A_3_B_3_ pattern end up in two distinct structures. On the one hand, we obtain a switch fully functionalized with a relatively weak EDG (CH_3_) and on the other hand, a combination of strong EWGs (NO_2_) and strong EDGs (NH_2_) is discovered. Even though the fully methyl substituted topological switch (**28R(** **CH**_**3**_ **_CH**_**3**_ **)** → **28M(** **CH**_**3**_ **_CH**_**3**_ **)**) shows a fairly high *β*_*HRS*_ ratio, our proof of concept puts forward other fully substituted candidate switches (*e.g.,* OH, F) with a higher contrast than this switch. What is more, no improvement over the parent topological switch is seen. During the BFS run, multiple **28M** structures are generated for which a considerably more enhanced NLO response is registered, examples being **CH**_**3**_ **_CN**, **CH**_**3**_ **_OH** and **NO**_**2**_ **_CH**_**3**_. However, none of these **28M** structures can be associated with a low response of the **28R**. Therefore, the same guiding features are encountered here: the ratio-based contrast optimizes, when possible, to a centrosymmetric OFF state and an enhanced ON state. Consequently, the optimization pathway can get stuck in such a topological switch, if it is the only one present having *C*_*i*_ symmetry. The difference-based contrast maximization is, however, a whole different story. The **28R(** **NO**_**2**_ **_NH**_**2**_ **)** → **28M(** **NO**_**2**_ **_NH**_**2**_ **)** maximum displays very different characteristics from the ratio-based optimum. The OFF state’s *β*_*HRS*_ now has the same order of magnitude as the ON state (1.07 × 10^4^ versus 1.95 × 10^4^ a.u.) but the switch still displays a difference of 8.77 × 10^3^ a.u., the highest difference-based contrast in this study. These augmented *β*_*HRS*_ responses are again due to a push-pull configuration. In contrast to the redox interconversion, where switches fully substituted with the same EDG, regardless of the presence of a centrosymmetric OFF state, displayed a significant difference-based contrast, a similar topological switch performs an order of magnitude worse due to a lower *β*_*HRS*_ response of the Möbius ON state. The **28R** → **28M** topological switch in substitution pattern A_3_B_3_ benefits more from push-pull functionalization than the **28R** → **26R** redox switch. Lastly, we observe that for some of the functionalized structures the **28R** becomes the ON state for both the ratio and difference contrast optimization, generally because of reduced *β*_*HRS*_ values for the **28M** conformation, examples being **CH**_**3**_ **_H**, **CH**_**3**_ **_F**, and **H_CH**_**3**_ with difference-based contrast values up until 10^3^ a.u.

Similarly to the redox interconversion, the topological switches visited during the inverse design procedures using the last substitution pattern A_2_BC_2_D do not have a high NLO ratio contrast, mainly because none of the OFF states belong to the *C*_*i*_ point group. In contrast, some of the switches show a high NLO difference, even though the ON and OFF states are of the same order of magnitude, as we also noted for the A_3_B_3_ substitution pattern. Both switches present a push-pull design with substituents ranging in electronic character, from the mesomerically EWGs, NO_2_ and CN, over the neutral H and rather weak inductive EDG, CH_3_, to mesomerically EDGs, OH and NH_2_. The ratio-optimized switch, **28R(** **CH**_**3**_ **_H_NO**_**2**_ **_NO**_**2**_ **)** → **28M(** **CH**_**3**_ **_H_NO**_**2**_ **_NO**_**2**_ **)**, combines a strongly electron-withdrawing side with a moderately electron-donating side. The presence of EWGs on positions R_4_/R_6_ is vital to get a ratio above 1, as these functional groups give rise to the highest response for the **28M** as well as the lowest response for the **28R**. The same is observed for the nitro group on site R_5_. Strangely, no functionalization is preferred over the hydrogen atom on position R_2_. Placing a strongly EWG (significantly) augments the *β*_*HRS*_ of the ON state; compare, for example, the 1.43 × 10^4^ a.u. of **28M(** **CH**_**3**_ **_** **NO**_**2**_ **_NO**_**2**_ **_NO**_**2**_ **)** with the 6.54 × 10^3^ a.u. of the optimum (**28R(** **CH**_**3**_ **_** **NO**_**2**_ **_NO**_**2**_ **_NO**_**2**_ **)** → **28M(** **CH**_**3**_ **_** **NO**_**2**_ **_NO**_**2**_ **_NO**_**2**_ **)**. Unfortunately, the OFF state’s NLO response increases as well, resulting in a ratio-based contrast that is about half of the contrast of the optimum. Finally, the methyl groups on R_1_/R_3_ seem to provide a good trade-off between a *β*_*HRS*_ that is low enough for **28R** and enhanced enough for **28M**. The optimal **28R(** **NH**_**2**_ **_CN_OH_CN)** → **28M(** **NH**_**2**_ **_CN_OH_CN)** switch for the difference optimization has a analogous type of motif as the A_3_B_3_ pattern optimum, in which 3 mesomerically EDGs are combined with 3 mesomerically EWGs. Despite the larger flexibility in the switch design of A_2_BC_2_D hexaphyrins, i.e., 4 (pairs of) sites are optimized versus 2 site combinations in A_3_B_3_, the A_3_B_3_ optimum performs 60% better. Nonetheless, during the NLO ratio optimization, another switch was found with a higher NLO difference than the best switch of our A_2_BC_2_D difference optimization: **28R(** **CH**_**3**_ **_NO**_**2**_ **_NO**_**2**_ **_NO**_**2**_ **)** → **28M(** **CH**_**3**_ **_NO**_**2**_ **_NO**_**2**_ **_NO**_**2**_ **)** with a difference of 8.35 × 10^3^ a.u. With respect to the fully NO_2_ substituted structure from our proof of concept, this switch has a 7 times larger difference. Hence, changing two positions (here R_1_ and R_3_) within the substitution pattern can drastically change the NLO difference. An even more convincing illustration of the large impact of the peripheral functionalization is the interchange of ON and OFF state when the strongly EWGs are replaced by one of the other possible functionalizations on the R_4_/R_6_ positions in the final BFS iterations. Strikingly, by placing two hydroxyl groups on those sites, the topological switch **28M(** **CH**_**3**_ **_H_OH_NO**_**2**_ **)** → **28R(** **CH**_**3**_ **_H_OH_NO**_**2**_ **)** is, displaying a *β*_*HRS*_ difference of 7.92 × 10^3^ a.u., which is at the same level of our most improved **28R** → **28M** switches. These findings corroborate that the positioning, the type, and the number of functionalizations are all indisputably key players in the design of efficient NLO hexaphyrin switches.

## 5 Conclusion

In this work, we discovered efficient nonlinear optical (NLO) switches with high *β*_*HRS*_ contrasts by applying the Best-First Search (BFS) algorithm. These innovative switches are based on *meso*-substituted hexaphyrins with different oxidation states and *π*-conjugation topologies and contain various electron-donating and electron-withdrawing substituents. Initially, we unveiled the structure-property relationships involving their aromaticity and NLO properties. As a proof of concept, we investigated the substituent effect on the geometry and (nonlinear) optical properties of different *meso*-substituted hexaphyrins previously reported in the literature. With the help of an inverse molecular design algorithm, the NLO contrasts (for which two definitions were taken into account, namely the ratio and the differences of the *β*_*HRS*_ responses of the ON and OFF states) of the most promising redox and topological switches were maximized by chemically modifying the ON and OFF states.

Unsubstituted [26] and [28] hexaphyrins prefer planar conformations driven by the low ring strain and high *π*-conjugation. A close relationship is found between the number of *π*-electrons, the topology and aromaticity as revealed by magnetic, electronic and reactivity aromaticity descriptors. While **26D**, **26R** and **28M** are aromatic macrocycles, **28R** is highly antiaromatic. The aromaticity is also reflected in their molecular orbital diagrams. Symmetry is the dominating factor in tweaking the *β*_*HRS*_ response of the parent structures. Other, contributing factors are the planarity and the ring strain of the macrocycle. The highest NLO contrasts are associated to redox or topological switches consisting of an OFF state (**28R**) and ON state (**26R** and **28M**) with low and high *β*_*HRS*_ values, respectively. Changes in topology, aromaticity and symmetry are responsible for the high NLO contrasts.

The effect of *meso*-substitution on the *β*_*HRS*_ response of hexaphyrins was explored in detail. A proof of concept was included in which fully substituted macrocycles and 4 additional substitution patterns were evaluated, carefully selected from currently synthesizable hexaphyrins and a previous computational study. In general, *meso*-substituents distort the planarity of the macrocycle. As a consequence, the ring strain increases leading to an enhanced *β*_*HRS*_ response. The degree of the distortion depends on the number of introduced functional groups as well as their electronic character. Nonlinear optical properties (*β*_*HRS*_) can be enhanced by the introduction of different types of *meso*-substituents, especially by EDGs and push-pull combinations of substituents. A high difference in symmetry between the ON and OFF state of the switch significantly increases the NLO ratio. The results for the difference-based contrast are more subtle.

Multiple inverse molecular design procedures were performed, starting from three distinct substitution patterns, to design redox (**28R** **→** **26R**) and topological (**28R** **→** **28M**) switches with maximal NLO contrasts. Depending on the number, type and position of *meso*-substituents, ratio-based *β*_*HRS*_ contrasts up to 15 times larger for the redox switch and 2 times larger for the topological switch, compared to the unsubstituted versions, were discovered. For the difference-based contrast definition, the improvement is even more pronounced, reaching values 25 times larger for the redox switch and 6 times for the topological switch. Hence, the efficiency of the **28R** **→** **26R** redox switch can be enhanced upon *meso*-substitution to a much greater extent than for the **28R** **→** **28M** Hückel-Möbius switch. When the NLO response ratio is considered, both the optimal *meso*-substituted redox and topology switches show a preference for EDGs in combination with a centrosymmetric OFF state. Symmetry, more precisely the presence or absence of an inversion center, plays a pivotal role in tweaking the OFF state’s *β*_*HRS*_ as it can vary to a larger extent than the ON state’s response, from zero when in *C*_*i*_ symmetry to the order of 10^3^ a.u. *Meso*-substitution can induce profound changes in the symmetry resulting in a wide spectrum of different NLO responses. When centrosymmetry is far less likely due to the selected substitution pattern, *e.g.* A_2_BC_2_D, push-pull functionalizations take the upper hand. Nonetheless, the absence of low-response OFF states significantly reduces the ratio, despite higher ON state values. The difference-based contrast maximizations tend to almost always converge to push-pull configurations with OFF states showing a minimal NLO response. The main difference between the redox and topology switches is that EDGs generally outnumber EWGs for the former, while the reverse is true for the latter. On a final note, we would like to point out that replacing functional groups by substituents of the opposite nature can in some cases lead to a reversal of ON/OFF switching behaviour of the redox and especially topological switch, displaying more or less equivalent NLO contrasts.

## Data Availability

The original contributions presented in the study are included in the article/Supplementary Material, further inquiries can be directed to the corresponding authors.

## Footnotes

1 Even though we did not carry out a full conformational analysis, multiple conformations were computed, retaining only the energetically most stable ones for the subsequent NLO calculations.
