# Supplementary material for: Fine-Tuning of Nonlinear Optical Contrasts of Hexaphyrin-Based Molecular Switches Using Inverse Design
Source: Front Chem. 2021 Dec 3;9:786036. doi: 10.3389/fchem.2021.786036 (PMC8677951; doi:10.3389/fchem.2021.786036)
Supplement: Supplementary file 1 [file DataSheet1.PDF]

## Supplementary Material

## 1 PARENTS AND *MESO*-SUBSTITUTED HEXAPHYRINS

**Table S1.** Static and dynamic NLO contrasts based on the hyper-Rayleigh scattering first hyperpolarizability for different ON and OFF states.[d]

| | | OFF-STATES | | | |
|---|---|---|---|---|---|
| | | **26D** | **26R** | **28R** | **28M** |
| **ON-STATES** | **26D**[a] | $1.00 \times 10^{0}$ | $7.79 \times 10^{-1}$ | $1.83 \times 10^{6}$ | $1.24 \times 10^{0}$ |
| | $\omega_1^b$ | $1.00 \times 10^{0}$ | $6.47 \times 10^{-1}$ | $4.07 \times 10^{6}$ | $1.52 \times 10^{0}$ |
| | $\omega_2^c$ | $1.00 \times 10^{0}$ | $6.19 \times 10^{-1}$ | $4.07 \times 10^{6}$ | $7.17 \times 10^{-1}$ |
| | **26R**[a] | $1.28 \times 10^{0}$ | $1.00 \times 10^{0}$ | $2.35 \times 10^{6}$ | $1.60 \times 10^{0}$ |
| | $\omega_1^b$ | $1.55 \times 10^{0}$ | $1.00 \times 10^{0}$ | $5.43 \times 10^{6}$ | $1.02 \times 10^{0}$ |
| | $\omega_2^c$ | $1.61 \times 10^{0}$ | $1.00 \times 10^{0}$ | $6.57 \times 10^{6}$ | $1.16 \times 10^{0}$ |
| | **28R**[a] | $7.14 \times 10^{-6}$ | $5.56 \times 10^{-6}$ | $1.00 \times 10^{0}$ | $8.85 \times 10^{-6}$ |
| | $\omega_1^b$ | $6.01 \times 10^{-6}$ | $3.89 \times 10^{-6}$ | $1.00 \times 10^{0}$ | $9.14 \times 10^{-6}$ |
| | $\omega_2^c$ | $2.11 \times 10^{-5}$ | $1.31 \times 10^{-5}$ | $1.00 \times 10^{0}$ | $1.51 \times 10^{-5}$ |
| | **28M**[a] | $8.04 \times 10^{-1}$ | $6.26 \times 10^{-1}$ | $1.47 \times 10^{6}$ | $1.00 \times 10^{0}$ |
| | $\omega_1^b$ | $6.58 \times 10^{-1}$ | $4.26 \times 10^{-1}$ | $2.31 \times 10^{6}$ | $1.00 \times 10^{0}$ |
| | $\omega_2^c$ | $1.40 \times 10^{0}$ | $8.64 \times 10^{-1}$ | $5.68 \times 10^{6}$ | $1.00 \times 10^{0}$ |

[a] NLO contrasts based on the first hyper-Rayleigh scattering in static regime. [b] NLO contrasts based on the first hyper-Rayleigh scattering in dynamic regime at a frequency of 0.653 eV ($\lambda_{Ho:YAG}$, 1907 nm). [c] NLO contrasts based on the first hyper-Rayleigh scattering in dynamic regime at a frequency of 1.165 eV ($\lambda_{Nd:YAG}$, 1064 nm). [d] If the OFF state has a $\beta_{HRS}$ response below 10 a.u., a value of 0.001 a.u. is chosen as OFF-state to compute the contrast

**Table S2.** Static and dynamic NLO differences based on the hyper-Rayleigh scattering first hyperpolarizability for different ON and OFF states.

| | | OFF-STATES | | | |
|---|---|---|---|---|---|
| | | **26D** | **26R** | **28R** | **28M** |
| **ON-STATES** | **26D**[a] | $0.00 \times 10^{0}$ | $-5.20 \times 10^{2}$ | $1.83 \times 10^{3}$ | $3.58 \times 10^{2}$ |
| | $\omega_1^b$ | $0.00 \times 10^{0}$ | $-1.92 \times 10^{3}$ | $3.51 \times 10^{3}$ | $1.20 \times 10^{3}$ |
| | $\omega_2^c$ | $0.00 \times 10^{0}$ | $-2.50 \times 10^{3}$ | $4.07 \times 10^{3}$ | $-1.61 \times 10^{3}$ |
| | **26R**[a] | $5.20 \times 10^{2}$ | $0.00 \times 10^{0}$ | $2.35 \times 10^{3}$ | $8.77 \times 10^{2}$ |
| | $\omega_1^b$ | $1.92 \times 10^{3}$ | $0.00 \times 10^{0}$ | $5.43 \times 10^{3}$ | $3.59 \times 10^{1}$ |
| | $\omega_2^c$ | $2.50 \times 10^{3}$ | $0.00 \times 10^{0}$ | $6.57 \times 10^{3}$ | $8.91 \times 10^{2}$ |
| | **28R**[a] | $-1.83 \times 10^{3}$ | $-2.35 \times 10^{3}$ | $0.00 \times 10^{0}$ | $-1.47 \times 10^{3}$ |
| | $\omega_1^b$ | $-3.51 \times 10^{3}$ | $-5.43 \times 10^{3}$ | $0.00 \times 10^{0}$ | $-2.31 \times 10^{3}$ |
| | $\omega_2^c$ | $-4.07 \times 10^{3}$ | $-6.57 \times 10^{3}$ | $0.00 \times 10^{0}$ | $-5.68 \times 10^{3}$ |
| | **28M**[a] | $-3.58 \times 10^{2}$ | $-8.77 \times 10^{2}$ | $1.47 \times 10^{3}$ | $0.00 \times 10^{0}$ |
| | $\omega_1^b$ | $-1.20 \times 10^{3}$ | $-3.12 \times 10^{3}$ | $2.31 \times 10^{3}$ | $0.00 \times 10^{0}$ |
| | $\omega_2^c$ | $1.61 \times 10^{3}$ | $-8.91 \times 10^{2}$ | $5.68 \times 10^{3}$ | $0.00 \times 10^{0}$ |

[a] NLO contrasts based on the first hyper-Rayleigh scattering in static regime. [b] NLO contrasts based on the first hyper-Rayleigh scattering in dynamic regime at a frequency of 1.165 eV ($\lambda_{Nd:YAG}$, 1064 nm). [c] NLO contrasts based on the first hyper-Rayleigh scattering in dynamic regime at a frequency of 0.653 eV ($\lambda_{Ho:YAG}$, 1907 nm).

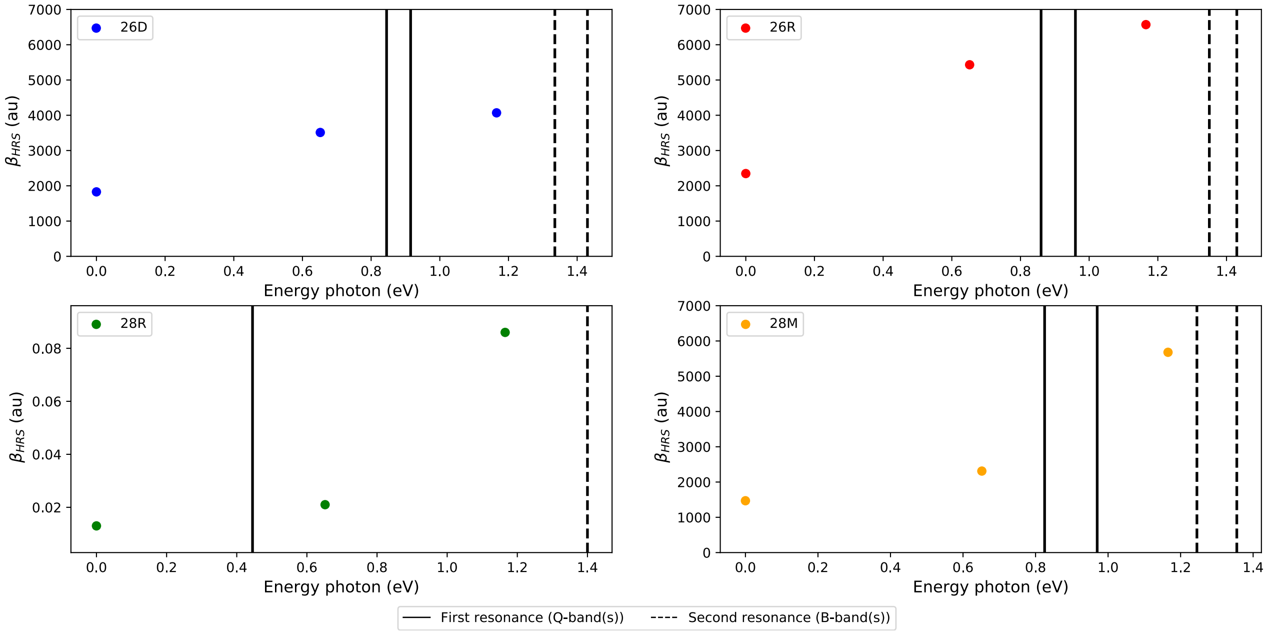


Figure S1: Evolution of $\beta_{HRS}$ in the gas phase with the incident energy of the photon at both frequencies in all unsubstituted hexaphyrins with the first and second resonance.

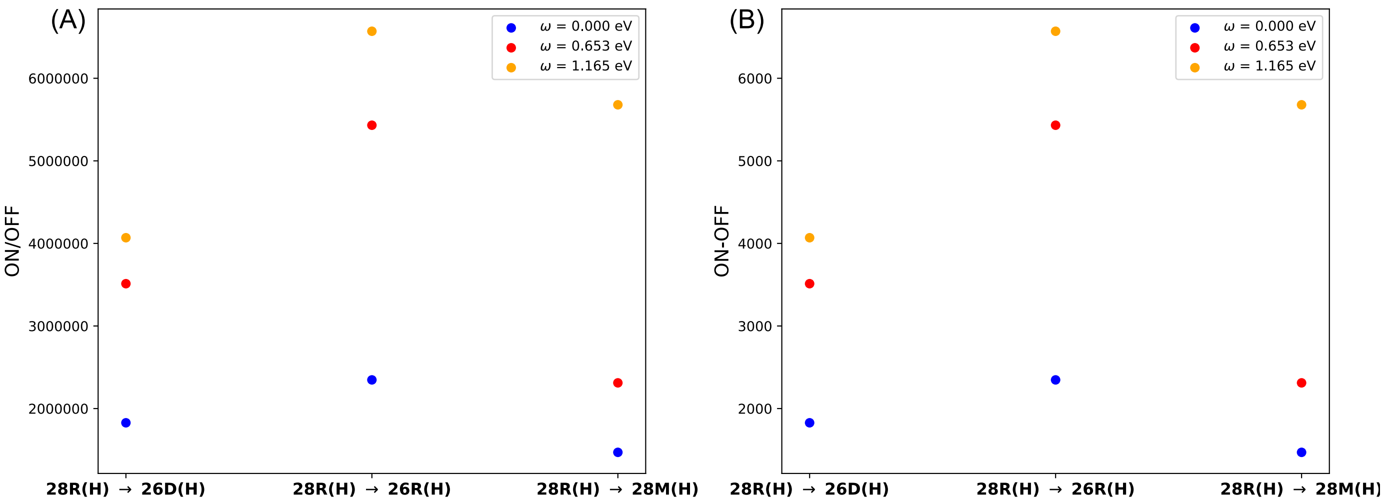


Figure S2: Comparison of the switch performance based on the ratio (A) and the difference (B) of the $\beta_{HRS}$ response of the unsubstituted macrocycles in both the static and dynamic regimes.

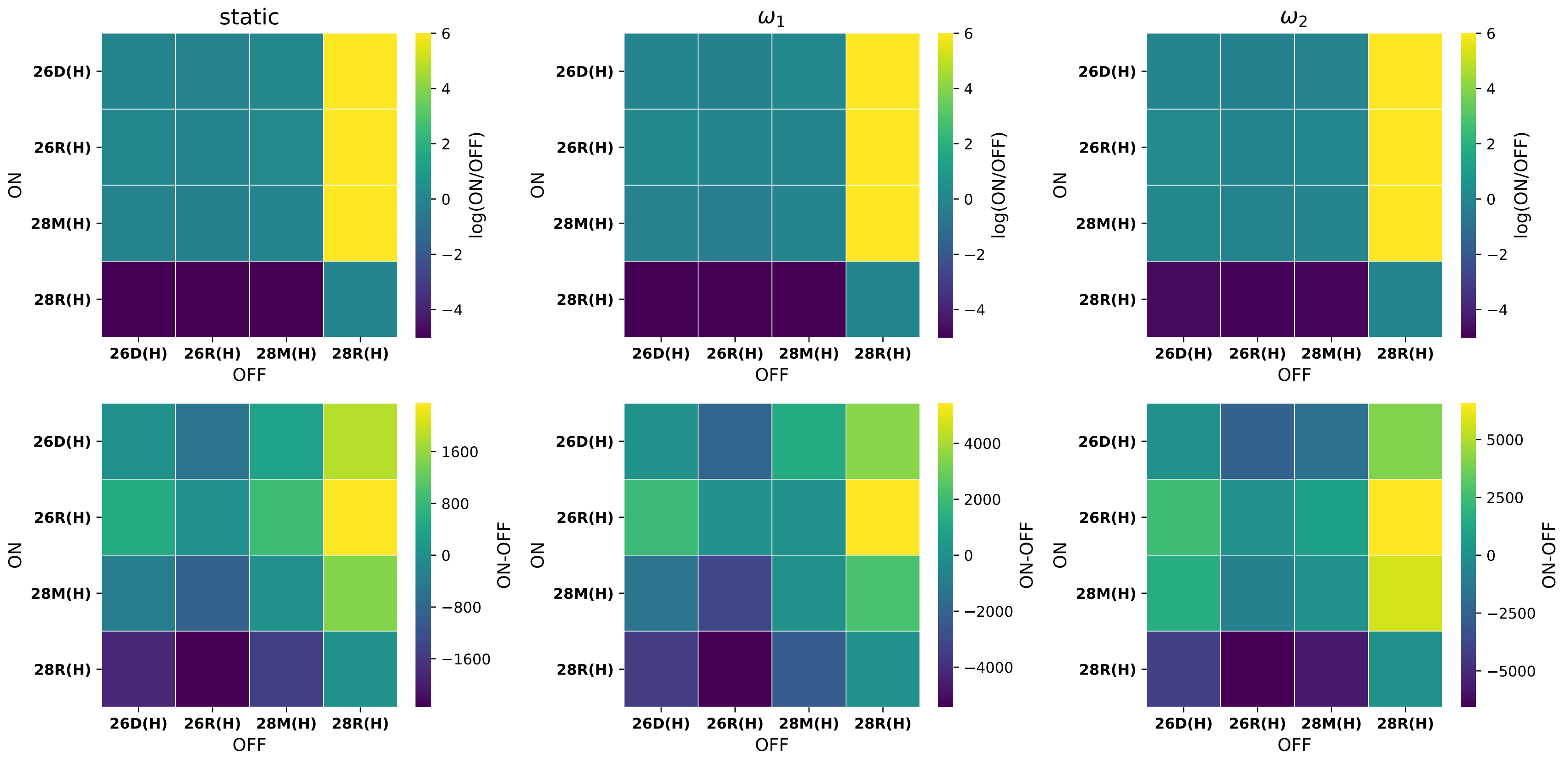


Figure S3: Heatmaps of both $\beta_{HRS}$ contrasts (ratio and difference) defined for the different ON- and OFF-states in the both static and dynamic regime at a frequency of 0.653 eV ($\omega_1$) and 1.165 eV ($\omega_2$).

# 2 INVERSE DESIGN

## 2.1 Inverse Design: 28R ⇋ 26R switch

### 2.1.1 $R_{1,4}$, $R_{2,5}$ and $R_{3,6}$ positions: ratio based contrast definition BFS

**Table S3.** Global iteration structures of the BFS procedure on the maximization of the ratio based contrast of the **26R** ⇋ **28R** switch substituting the $R_{1,4}$, $R_{2,5}$ and $R_{3,6}$ positions. The static hyper-Rayleigh scattering first hyperpolarizability values of the [26]hexaphyrins and [28]hexaphyrins are given in a.u.[a]

| **Global iteration 1** | $R_{1,4}$_$R_{2,5}$_$R_{3,6}$ | $\beta_{HRS}$**(28R)** | $\beta_{HRS}$**(26R)** | **contrast (ratio)** | **contrast (difference)** |
|---|---|---|---|---|---|
| Substitution $R_{1,4}$ | H_OH_$NH_2$ | $1.00 \times 10^{-3}$ | $1.47 \times 10^{4}$ | $1.47 \times 10^{7}$ | $1.47 \times 10^{4}$ |
| | F_OH_$NH_2$ | $1.00 \times 10^{-3}$ | $2.02 \times 10^{4}$ | $2.02 \times 10^{7}$ | $2.02 \times 10^{4}$ |
| | CN_OH_$NH_2$ | $1.00 \times 10^{-3}$ | $1.01 \times 10^{3}$ | $1.01 \times 10^{6}$ | $1.01 \times 10^{3}$ |
| | $CH_3$_OH_$NH_2$ | $1.00 \times 10^{-3}$ | $1.39 \times 10^{4}$ | $1.39 \times 10^{7}$ | $1.39 \times 10^{4}$ |
| | $NH_2$_OH_$NH_2$ | $1.00 \times 10^{-3}$ | $3.52 \times 10^{4}$ | $3.52 \times 10^{7}$ | $3.52 \times 10^{4}$ |
| | OH_OH_$NH_2$ | $1.62 \times 10^{3}$ | $2.75 \times 10^{4}$ | $1.70 \times 10^{1}$ | $2.58 \times 10^{4}$ |
| | $NO_2$_OH_$NH_2$ | $1.74 \times 10^{3}$ | $1.36 \times 10^{4}$ | $7.81 \times 10^{0}$ | $1.19 \times 10^{4}$ |
| Substitution $R_{2,5}$ | $NH_2$_H_$NH_2$ | $1.00 \times 10^{-3}$ | $2.98 \times 10^{4}$ | $2.98 \times 10^{7}$ | $2.98 \times 10^{4}$ |
| | $NH_2$_F_$NH_2$ | $1.00 \times 10^{-3}$ | $3.16 \times 10^{4}$ | $3.16 \times 10^{7}$ | $3.16 \times 10^{4}$ |
| | $NH_2$_CN_$NH_2$ | $1.00 \times 10^{-3}$ | $1.76 \times 10^{4}$ | $1.76 \times 10^{7}$ | $1.76 \times 10^{4}$ |
| | $NH_2$_$CH_3$_$NH_2$ | $2.41 \times 10^{1}$ | $3.57 \times 10^{4}$ | $1.48 \times 10^{3}$ | $3.57 \times 10^{4}$ |
| | $NH_2$_$NH_2$_$NH_2$ | $2.70 \times 10^{3}$ | $3.37 \times 10^{4}$ | $1.25 \times 10^{1}$ | $3.10 \times 10^{4}$ |
| | $NH_2$_OH_$NH_2$ | $1.00 \times 10^{-3}$ | $3.52 \times 10^{4}$ | $3.52 \times 10^{7}$ | $3.52 \times 10^{4}$ |
| | $NH_2$_$NO_2$_$NH_2$ | $1.74 \times 10^{3}$ | $1.36 \times 10^{4}$ | $7.81 \times 10^{0}$ | $1.19 \times 10^{4}$ |
| Substitution $R_{3,6}$ | $NH_2$_OH_H | $1.00 \times 10^{-3}$ | $1.98 \times 10^{4}$ | $1.98 \times 10^{7}$ | $1.98 \times 10^{4}$ |
| | $NH_2$_OH_F | $1.00 \times 10^{-3}$ | $2.72 \times 10^{4}$ | $2.72 \times 10^{7}$ | $2.72 \times 10^{4}$ |
| | $NH_2$_OH_CN | $1.00 \times 10^{-3}$ | $2.88 \times 10^{4}$ | $2.88 \times 10^{7}$ | $2.88 \times 10^{4}$ |
| | $NH_2$_OH_$CH_3$ | $1.00 \times 10^{-3}$ | $2.16 \times 10^{4}$ | $2.16 \times 10^{7}$ | $2.16 \times 10^{4}$ |
| | $NH_2$_OH_$NH_2$ | $1.00 \times 10^{-3}$ | $3.52 \times 10^{4}$ | $3.52 \times 10^{7}$ | $3.52 \times 10^{4}$ |
| | $NH_2$_OH_OH | $1.46 \times 10^{3}$ | $5.97 \times 10^{2}$ | $4.10 \times 10^{-1}$ | $-8.58 \times 10^{2}$ |
| | $NH_2$_OH_$NO_2$ | $1.00 \times 10^{-3}$ | $1.69 \times 10^{4}$ | $1.69 \times 10^{7}$ | $1.69 \times 10^{4}$ |
| **Global iteration 2** | $R_{1,4}$_$R_{2,5}$_$R_{3,6}$ | $\beta_{HRS}$**(28R)** | $\beta_{HRS}$**(26R)** | **contrast (ratio)** | **contrast (difference)** |
| Substitution $R_{3,6}$ | $NH_2$_OH_H | $1.00 \times 10^{-3}$ | $1.98 \times 10^{4}$ | $1.98 \times 10^{7}$ | $1.98 \times 10^{4}$ |
| | $NH_2$_OH_F | $1.00 \times 10^{-3}$ | $2.72 \times 10^{4}$ | $2.72 \times 10^{7}$ | $2.72 \times 10^{4}$ |
| | $NH_2$_OH_CN | $1.00 \times 10^{-3}$ | $2.88 \times 10^{4}$ | $2.88 \times 10^{7}$ | $2.88 \times 10^{4}$ |
| | $NH_2$_OH_$CH_3$ | $1.00 \times 10^{-3}$ | $2.16 \times 10^{4}$ | $2.16 \times 10^{7}$ | $2.16 \times 10^{4}$ |
| | $NH_2$_OH_$NH_2$ | $1.00 \times 10^{-3}$ | $3.52 \times 10^{4}$ | $3.52 \times 10^{7}$ | $3.52 \times 10^{4}$ |
| | $NH_2$_OH_OH | $1.46 \times 10^{3}$ | $5.97 \times 10^{2}$ | $4.10 \times 10^{-1}$ | $-8.58 \times 10^{2}$ |
| | $NH_2$_OH_$NO_2$ | $1.00 \times 10^{-3}$ | $1.69 \times 10^{4}$ | $1.69 \times 10^{7}$ | $1.69 \times 10^{4}$ |
| Substitution $R_{1,4}$ | H_OH_$NH_2$ | $1.00 \times 10^{-3}$ | $1.47 \times 10^{4}$ | $1.47 \times 10^{7}$ | $1.47 \times 10^{4}$ |
| | F_OH_$NH_2$ | $1.00 \times 10^{-3}$ | $2.02 \times 10^{4}$ | $2.02 \times 10^{7}$ | $2.02 \times 10^{4}$ |
| | CN_OH_$NH_2$ | $1.00 \times 10^{-3}$ | $1.01 \times 10^{3}$ | $1.01 \times 10^{6}$ | $1.01 \times 10^{3}$ |
| | $CH_3$_OH_$NH_2$ | $1.00 \times 10^{-3}$ | $1.39 \times 10^{4}$ | $1.39 \times 10^{7}$ | $1.39 \times 10^{4}$ |
| | $NH_2$_OH_$NH_2$ | $1.00 \times 10^{-3}$ | $3.52 \times 10^{4}$ | $3.52 \times 10^{7}$ | $3.52 \times 10^{4}$ |
| | OH_OH_$NH_2$ | $1.62 \times 10^{3}$ | $2.75 \times 10^{4}$ | $1.70 \times 10^{1}$ | $2.58 \times 10^{4}$ |
| | $NO_2$_OH_$NH_2$ | $1.74 \times 10^{3}$ | $1.36 \times 10^{4}$ | $7.81 \times 10^{0}$ | $1.19 \times 10^{4}$ |
| Substitution $R_{2,5}$ | $NH_2$_H_$NH_2$ | $1.00 \times 10^{-3}$ | $2.98 \times 10^{4}$ | $2.98 \times 10^{7}$ | $2.98 \times 10^{4}$ |
| | $NH_2$_F_$NH_2$ | $1.00 \times 10^{-3}$ | $3.16 \times 10^{4}$ | $3.16 \times 10^{7}$ | $3.16 \times 10^{4}$ |
| | $NH_2$_CN_$NH_2$ | $1.00 \times 10^{-3}$ | $1.76 \times 10^{4}$ | $1.76 \times 10^{7}$ | $1.76 \times 10^{4}$ |
| | $NH_2$_$CH_3$_$NH_2$ | $2.41 \times 10^{1}$ | $3.57 \times 10^{4}$ | $1.48 \times 10^{3}$ | $3.57 \times 10^{4}$ |
| | $NH_2$_$NH_2$_$NH_2$ | $2.70 \times 10^{3}$ | $3.37 \times 10^{4}$ | $1.25 \times 10^{1}$ | $3.10 \times 10^{4}$ |
| | $NH_2$_OH_$NH_2$ | $1.00 \times 10^{-3}$ | $3.52 \times 10^{4}$ | $3.52 \times 10^{7}$ | $3.52 \times 10^{4}$ |
| | $NH_2$_$NO_2$_$NH_2$ | $1.74 \times 10^{3}$ | $1.36 \times 10^{4}$ | $7.81 \times 10^{0}$ | $1.19 \times 10^{4}$ |

[a] These substitution patterns show a $\beta_{HRS}$**(28R)** below 10 a.u.. Their respective contrasts are computed considering a value of 0.001 for the $\beta_{HRS}$**(28R)**. For the difference definition the real value is used to compute the difference.

### 2.1.2 $R_{1,4}$, $R_{2,5}$ and $R_{3,6}$ positions: difference based contrast definition BFS

**Table S4.** Global iteration structures of the BFS procedure on the maximization of the difference based contrast of the **26R** $\leftrightharpoons$ **28R** switch substituting the $R_{1,4}$, $R_{2,5}$ and $R_{3,6}$ positions. The static hyper-Rayleigh scattering first hyperpolarizability values of the [26]hexaphyrins and [28]hexaphyrins are given in a.u.

| **Global iteration 1** | **$R_{1,4}$_$R_{2,5}$_$R_{3,6}$** | **$\beta_{HRS}$(28R)** | **$\beta_{HRS}$(26R)** | **contrast (ratio)** | **contrast (difference)** |
|---|---|---|---|---|---|
| | H_OH_CN | $1.79 \times 10^{0}$ | $6.28 \times 10^{3}$ | $6.28 \times 10^{6}$ | $6.27 \times 10^{3}$ |
| | F_OH_CN | $1.19 \times 10^{0}$ | $1.00 \times 10^{4}$ | $1.00 \times 10^{7}$ | $1.00 \times 10^{4}$ |
| | CN_OH_CN | $5.50 \times 10^{2}$ | $5.37 \times 10^{3}$ | $9.77 \times 10^{0}$ | $4.82 \times 10^{3}$ |
| Substitution $R_{1,4}$ | $CH_3$_OH_CN | $9.48 \times 10^{2}$ | $5.98 \times 10^{3}$ | $6.31 \times 10^{0}$ | $5.03 \times 10^{3}$ |
| | $NH_2$_OH_CN | $2.48 \times 10^{0}$ | $2.88 \times 10^{4}$ | $2.88 \times 10^{7}$ | $2.88 \times 10^{4}$ |
| | OH_OH_CN | $1.14 \times 10^{3}$ | $1.89 \times 10^{4}$ | $1.66 \times 10^{1}$ | $1.77 \times 10^{4}$ |
| | $NO_2$_OH_CN | $1.18 \times 10^{3}$ | $5.09 \times 10^{3}$ | $4.32 \times 10^{0}$ | $3.91 \times 10^{3}$ |
| | $NH_2$_OH_H | $2.11 \times 10^{0}$ | $1.98 \times 10^{4}$ | $1.98 \times 10^{7}$ | $1.98 \times 10^{4}$ |
| | $NH_2$_OH_F | $1.91 \times 10^{0}$ | $2.72 \times 10^{4}$ | $2.72 \times 10^{7}$ | $2.72 \times 10^{4}$ |
| | $NH_2$_OH_CN | $2.48 \times 10^{0}$ | $2.88 \times 10^{4}$ | $2.88 \times 10^{7}$ | $2.88 \times 10^{4}$ |
| Substitution $R_{3,6}$ | $NH_2$_OH_$CH_3$ | $2.36 \times 10^{0}$ | $2.16 \times 10^{4}$ | $2.16 \times 10^{7}$ | $2.16 \times 10^{4}$ |
| | $NH_2$_OH_$NH_2$ | $4.16 \times 10^{0}$ | $3.52 \times 10^{4}$ | $3.52 \times 10^{7}$ | $3.52 \times 10^{4}$ |
| | $NH_2$_OH_OH | $1.46 \times 10^{3}$ | $5.97 \times 10^{2}$ | $4.10 \times 10^{-1}$ | $-8.58 \times 10^{2}$ |
| | $NH_2$_OH_$NO_2$ | $1.58 \times 10^{0}$ | $1.69 \times 10^{4}$ | $1.69 \times 10^{7}$ | $1.69 \times 10^{4}$ |
| | $NH_2$_H_$NH_2$ | $3.12 \times 10^{-1}$ | $2.98 \times 10^{4}$ | $2.98 \times 10^{7}$ | $2.98 \times 10^{4}$ |
| | $NH_2$_F_$NH_2$ | $1.96 \times 10^{0}$ | $3.16 \times 10^{4}$ | $3.16 \times 10^{7}$ | $3.16 \times 10^{4}$ |
| | $NH_2$_CN_$NH_2$ | $1.46 \times 10^{-1}$ | $1.76 \times 10^{4}$ | $1.76 \times 10^{7}$ | $1.76 \times 10^{4}$ |
| Substitution $R_{2,5}$ | $NH_2$_$CH_3$_$NH_2$ | $2.41 \times 10^{1}$ | $3.57 \times 10^{4}$ | $1.48 \times 10^{3}$ | $3.57 \times 10^{4}$ |
| | $NH_2$_$NH_2$_$NH_2$ | $2.70 \times 10^{3}$ | $3.37 \times 10^{4}$ | $1.25 \times 10^{1}$ | $3.10 \times 10^{4}$ |
| | $NH_2$_OH_$NH_2$ | $4.16 \times 10^{0}$ | $3.52 \times 10^{4}$ | $3.52 \times 10^{7}$ | $3.52 \times 10^{4}$ |
| | $NH_2$_$NO_2$_$NH_2$ | $7.07 \times 10^{2}$ | $5.94 \times 10^{4}$ | $8.41 \times 10^{1}$ | $5.87 \times 10^{4}$ |
| **Global iteration 2** | **$R_{1,4}$_$R_{2,5}$_$R_{3,6}$** | **$\beta_{HRS}$(28R)** | **$\beta_{HRS}$(26R)** | **contrast (ratio)** | **contrast (difference)** |
| | $NH_2$_H_$NH_2$ | $3.12 \times 10^{-1}$ | $2.98 \times 10^{4}$ | $2.98 \times 10^{7}$ | $2.98 \times 10^{4}$ |
| | $NH_2$_F_$NH_2$ | $1.96 \times 10^{0}$ | $3.16 \times 10^{4}$ | $3.16 \times 10^{7}$ | $3.16 \times 10^{4}$ |
| | $NH_2$_CN_$NH_2$ | $1.46 \times 10^{-1}$ | $1.76 \times 10^{4}$ | $1.76 \times 10^{7}$ | $1.76 \times 10^{4}$ |
| Substitution $R_{2,5}$ | $NH_2$_$CH_3$_$NH_2$ | $2.41 \times 10^{1}$ | $3.57 \times 10^{4}$ | $1.48 \times 10^{3}$ | $3.57 \times 10^{4}$ |
| | $NH_2$_$NH_2$_$NH_2$ | $2.70 \times 10^{3}$ | $3.37 \times 10^{4}$ | $1.25 \times 10^{1}$ | $3.10 \times 10^{4}$ |
| | $NH_2$_OH_$NH_2$ | $4.16 \times 10^{0}$ | $3.52 \times 10^{4}$ | $3.52 \times 10^{7}$ | $3.52 \times 10^{4}$ |
| | $NH_2$_$NO_2$_$NH_2$ | $7.07 \times 10^{2}$ | $5.94 \times 10^{4}$ | $8.41 \times 10^{1}$ | $5.87 \times 10^{4}$ |
| | $NH_2$_$NO_2$_H | $1.63 \times 10^{2}$ | $2.49 \times 10^{4}$ | $1.53 \times 10^{2}$ | $2.48 \times 10^{4}$ |
| | $NH_2$_$NO_2$ _F | $1.84 \times 10^{2}$ | $3.56 \times 10^{4}$ | $1.93 \times 10^{2}$ | $3.54 \times 10^{4}$ |
| | $NH_2$_$NO_2$_CN | $1.34 \times 10^{2}$ | $2.12 \times 10^{4}$ | $1.58 \times 10^{2}$ | $2.11 \times 10^{4}$ |
| Substitution $R_{3,6}$ | $NH_2$_$NO_2$_$CH_3$ | $5.88 \times 10^{2}$ | $2.32 \times 10^{4}$ | $3.96 \times 10^{1}$ | $2.27 \times 10^{4}$ |
| | $NH_2$_$NO_2$_$NH_2$ | $7.07 \times 10^{2}$ | $5.94 \times 10^{4}$ | $8.41 \times 10^{1}$ | $5.87 \times 10^{4}$ |
| | $NH_2$_$NO_2$_OH | $9.62 \times 10^{2}$ | $5.01 \times 10^{4}$ | $5.21 \times 10^{1}$ | $4.92 \times 10^{4}$ |
| | $NH_2$_$NO_2$_$NO_2$ | $1.56 \times 10^{3}$ | $1.92 \times 10^{4}$ | $1.23 \times 10^{1}$ | $1.77 \times 10^{4}$ |
| | H_$NO_2$_$NH_2$ | $5.54 \times 10^{2}$ | $2.20 \times 10^{4}$ | $3.96 \times 10^{1}$ | $2.14 \times 10^{4}$ |
| | F_$NO_2$_$NH_2$ | $4.82 \times 10^{2}$ | $3.04 \times 10^{4}$ | $6.31 \times 10^{1}$ | $2.99 \times 10^{4}$ |
| | CN_$NO_2$_$NH_2$ | $4.59 \times 10^{2}$ | $2.06 \times 10^{4}$ | $4.48 \times 10^{1}$ | $2.01 \times 10^{4}$ |
| Substitution $R_{1,4}$ | $CH_3$_$NO_2$_$NH_2$ | $1.14 \times 10^{3}$ | $1.80 \times 10^{4}$ | $1.58 \times 10^{1}$ | $1.69 \times 10^{4}$ |
| | $NH_2$_$NO_2$_$NH_2$ | $7.07 \times 10^{2}$ | $5.94 \times 10^{4}$ | $8.41 \times 10^{1}$ | $5.87 \times 10^{4}$ |
| | OH_$NO_2$_$NH_2$ | $1.23 \times 10^{3}$ | $4.14 \times 10^{4}$ | $3.38 \times 10^{1}$ | $4.02 \times 10^{4}$ |
| | $NO_2$_$NO_2$_$NH_2$ | $3.57 \times 10^{3}$ | $2.14 \times 10^{4}$ | $5.99 \times 10^{0}$ | $1.78 \times 10^{4}$ |

### 2.1.3 $R_{1,3,5}$, $R_{2,4,6}$: ratio based contrast definition BFS

**Table S5.** Global iteration structures of the BFS procedure on the maximization of the ratio based contrast of the **26R** ⇋ **28R** switch substituting the $R_{1,3,5}$, $R_{2,4,6}$ positions. The static hyper-Rayleigh scattering first hyperpolarizability values of the [26]hexaphyrins and [28]hexaphyrins are given in a.u.[a]

| **Global iteration 1** | **$R_{1,3,5}$_$R_{2,4,6}$** | **$\beta_{HRS}$(28R)** | **$\beta_{HRS}$(26R)** | **contrast (ratio)** | **contrast (difference)** |
|---|---|---|---|---|---|
| Substitution $R_{2,4,6}$ | $NH_2$_H | $2.78 \times 10^3$ | $1.55 \times 10^4$ | $5.57 \times 10^0$ | $1.27 \times 10^4$ |
| | $NH_2$_F | $1.78 \times 10^3$ | $2.19 \times 10^4$ | $1.23 \times 10^1$ | $2.01 \times 10^4$ |
| | $NH_2$_CN | $1.15 \times 10^4$ | $1.95 \times 10^4$ | $1.69 \times 10^0$ | $8.01 \times 10^3$ |
| | $NH_2$_$CH_3$ | $2.98 \times 10^3$ | $1.10 \times 10^4$ | $3.68 \times 10^0$ | $8.72 \times 10^3$ |
| | $NH_2$_$NH_2$ | $2.70 \times 10^3$ | $3.37 \times 10^4$ | $1.25 \times 10^1$ | $3.10 \times 10^4$ |
| | $NH_2$_OH | $1.12 \times 10^3$ | $3.01 \times 10^4$ | $2.68 \times 10^1$ | $2.80 \times 10^4$ |
| | $NH_2$_$NO_2$ | $1.22 \times 10^4$ | $1.56 \times 10^4$ | $1.28 \times 10^0$ | $3.43 \times 10^3$ |
| Substitution $R_{1,3,5}$ | H_OH | $2.82 \times 10^3$ | $1.01 \times 10^4$ | $3.58 \times 10^0$ | $7.25 \times 10^3$ |
| | F_OH | $1.65 \times 10^3$ | $1.54 \times 10^4$ | $9.35 \times 10^0$ | $1.38 \times 10^4$ |
| | CN_OH | $1.04 \times 10^4$ | $1.16 \times 10^4$ | $1.11 \times 10^0$ | $1.19 \times 10^3$ |
| | $CH_3$_OH | $3.59 \times 10^3$ | $1.25 \times 10^4$ | $3.49 \times 10^0$ | $8.94 \times 10^3$ |
| | $NH_2$_OH | $1.12 \times 10^3$ | $3.01 \times 10^4$ | $2.68 \times 10^1$ | $2.80 \times 10^4$ |
| | OH_OH | $1.00 \times 10^{-3}$ | $2.57 \times 10^4$ | $2.57 \times 10^7$ | $2.57 \times 10^4$ |
| | $NO_2$_OH | $9.03 \times 10^3$ | $8.65 \times 10^3$ | $9.57 \times 10^{-1}$ | $-3.86 \times 10^2$ |
| **Global iteration 2** | **$R_{1,3,5}$_$R_{2,4,6}$** | **$\beta_{HRS}$(28R)** | **$\beta_{HRS}$(26R)** | **contrast (ratio)** | **contrast (difference)** |
| Substitution $R_{1,3,5}$ | H_OH | $2.82 \times 10^3$ | $1.01 \times 10^4$ | $3.58 \times 10^0$ | $7.25 \times 10^3$ |
| | F_OH | $1.65 \times 10^3$ | $1.54 \times 10^4$ | $9.35 \times 10^0$ | $1.38 \times 10^4$ |
| | CN_OH | $1.04 \times 10^4$ | $1.16 \times 10^4$ | $1.11 \times 10^0$ | $1.19 \times 10^3$ |
| | $CH_3$_OH | $3.59 \times 10^3$ | $1.25 \times 10^4$ | $3.49 \times 10^0$ | $8.94 \times 10^3$ |
| | $NH_2$_OH | $1.12 \times 10^3$ | $3.01 \times 10^4$ | $2.68 \times 10^1$ | $2.80 \times 10^4$ |
| | OH_OH | $1.00 \times 10^{-3}$ | $2.57 \times 10^4$ | $2.57 \times 10^7$ | $2.57 \times 10^4$ |
| | $NO_2$_OH | $9.03 \times 10^3$ | $8.65 \times 10^3$ | $9.57 \times 10^{-1}$ | $-3.86 \times 10^2$ |
| Substitution $R_{1,3,5}$ | OH_H | $2.82 \times 10^3$ | $1.01 \times 10^4$ | $3.58 \times 10^0$ | $7.25 \times 10^3$ |
| | OH_F | $1.65 \times 10^3$ | $1.54 \times 10^4$ | $9.35 \times 10^0$ | $1.38 \times 10^4$ |
| | OH_CN | $1.04 \times 10^4$ | $1.16 \times 10^4$ | $1.11 \times 10^0$ | $1.19 \times 10^3$ |
| | OH_$CH_3$ | $3.59 \times 10^3$ | $1.25 \times 10^4$ | $3.49 \times 10^0$ | $8.94 \times 10^3$ |
| | OH_$NH_2$ | $1.12 \times 10^3$ | $3.01 \times 10^4$ | $2.68 \times 10^1$ | $2.80 \times 10^4$ |
| | OH_OH | $1.00 \times 10^{-3}$ | $2.57 \times 10^4$ | $2.57 \times 10^7$ | $2.57 \times 10^4$ |
| | OH_$NO_2$ | $9.03 \times 10^3$ | $8.65 \times 10^3$ | $9.57 \times 10^{-1}$ | $-3.86 \times 10^2$ |

[a] These substitution patterns show a $\beta_{HRS}$**(28R)** below 10 a.u.. Their respective contrasts are computed considering a value of 0.001 for the $\beta_{HRS}$**(28R)**. For the difference definition the real value is used to compute the difference.

### 2.1.4 $R_{1,3,5}$, $R_{2,4,6}$: difference based contrast definition BFS

**Table S6.** Global iteration structures of the BFS procedure on the maximization of the difference based contrast of the **26R** ⇋ **28R** switch substituting the $R_{1,3,5}$, $R_{2,4,6}$ positions. The static hyper-Rayleigh scattering first hyperpolarizability values of the [26]hexaphyrins and [28]hexaphyrins are given in a.u..

| **Global iteration 1** | **$R_{1,3,5}$_$R_{2,4,6}$** | **$\beta_{HRS}$(28R)** | **$\beta_{HRS}$(26R)** | **contrast (ratio)** | **contrast (difference)** |
|---|---|---|---|---|---|
| Substitution $R_{2,4,6}$ | $NH_2$_H | $2.78 \times 10^3$ | $1.55 \times 10^4$ | $5.57 \times 10^0$ | $1.27 \times 10^4$ |
| | $NH_2$_F | $1.78 \times 10^3$ | $2.19 \times 10^4$ | $1.23 \times 10^1$ | $2.01 \times 10^4$ |
| | $NH_2$_CN | $1.15 \times 10^4$ | $1.95 \times 10^4$ | $1.69 \times 10^0$ | $8.01 \times 10^3$ |
| | $NH_2$_$CH_3$ | $2.98 \times 10^3$ | $1.10 \times 10^4$ | $4.86 \times 10^0$ | $8.72 \times 10^3$ |
| | $NH_2$_$NH_2$ | $2.70 \times 10^3$ | $3.37 \times 10^4$ | $1.25 \times 10^1$ | $3.10 \times 10^4$ |
| | $NH_2$_OH | $1.12 \times 10^3$ | $3.01 \times 10^4$ | $2.68 \times 10^1$ | $2.80 \times 10^4$ |
| | $NH_2$_$NO_2$ | $1.07 \times 10^4$ | $1.57 \times 10^4$ | $1.46 \times 10^0$ | $4.93 \times 10^3$ |
| Substitution $R_{1,3,5}$ | H_$NH_2$ | $2.78 \times 10^3$ | $1.55 \times 10^4$ | $5.57 \times 10^0$ | $1.27 \times 10^4$ |
| | F_$NH_2$ | $1.78 \times 10^3$ | $2.19 \times 10^4$ | $1.23 \times 10^1$ | $2.01 \times 10^4$ |
| | CN_$NH_2$ | $1.15 \times 10^4$ | $1.95 \times 10^4$ | $1.69 \times 10^0$ | $7.99 \times 10^3$ |
| | $CH_3$_$NH_2$ | $2.98 \times 10^3$ | $1.10 \times 10^4$ | $4.86 \times 10^0$ | $8.72 \times 10^3$ |
| | $NH_2$_$NH_2$ | $2.70 \times 10^3$ | $3.37 \times 10^4$ | $1.25 \times 10^1$ | $3.10 \times 10^4$ |
| | OH_$NH_2$ | $1.12 \times 10^3$ | $3.01 \times 10^4$ | $2.68 \times 10^1$ | $2.80 \times 10^4$ |
| | $NO_2$_$NH_2$ | $1.07 \times 10^4$ | $1.57 \times 10^4$ | $1.46 \times 10^0$ | $4.93 \times 10^3$ |
| **Global iteration 2** | **$R_{1,3,5}$_$R_{2,4,6}$** | **$\beta_{HRS}$(28R)** | **$\beta_{HRS}$(26R)** | **contrast (ratio)** | **contrast (difference)** |
| Substitution $R_{1,3,5}$ | H_$NH_2$ | $2.78 \times 10^3$ | $1.55 \times 10^4$ | $5.57 \times 10^0$ | $1.27 \times 10^4$ |
| | F_$NH_2$ | $1.78 \times 10^3$ | $2.19 \times 10^4$ | $1.23 \times 10^1$ | $2.01 \times 10^4$ |
| | CN_$NH_2$ | $1.15 \times 10^4$ | $1.95 \times 10^4$ | $1.69 \times 10^0$ | $7.99 \times 10^3$ |
| | $CH_3$_$NH_2$ | $2.98 \times 10^3$ | $1.10 \times 10^4$ | $4.86 \times 10^0$ | $8.72 \times 10^3$ |
| | $NH_2$_$NH_2$ | $2.70 \times 10^3$ | $3.37 \times 10^4$ | $1.25 \times 10^1$ | $3.10 \times 10^4$ |
| | OH_$NH_2$ | $1.12 \times 10^3$ | $3.01 \times 10^4$ | $2.68 \times 10^1$ | $2.80 \times 10^4$ |
| | $NO_2$_$NH_2$ | $1.07 \times 10^4$ | $1.57 \times 10^4$ | $1.46 \times 10^0$ | $4.93 \times 10^3$ |
| Substitution $R_{2,4,6}$ | $NH_2$_H | $2.78 \times 10^3$ | $1.55 \times 10^4$ | $5.57 \times 10^0$ | $1.27 \times 10^4$ |
| | $NH_2$_F | $1.78 \times 10^3$ | $2.19 \times 10^4$ | $1.23 \times 10^1$ | $2.01 \times 10^4$ |
| | $NH_2$_CN | $1.15 \times 10^4$ | $1.95 \times 10^4$ | $1.69 \times 10^0$ | $8.01 \times 10^3$ |
| | $NH_2$_$CH_3$ | $2.98 \times 10^3$ | $1.10 \times 10^4$ | $4.86 \times 10^0$ | $8.72 \times 10^3$ |
| | $NH_2$_$NH_2$ | $2.70 \times 10^3$ | $3.37 \times 10^4$ | $1.25 \times 10^1$ | $3.10 \times 10^4$ |
| | $NH_2$_OH | $1.12 \times 10^3$ | $3.01 \times 10^4$ | $2.68 \times 10^1$ | $2.80 \times 10^4$ |
| | $NH_2$_$NO_2$ | $1.07 \times 10^4$ | $1.57 \times 10^4$ | $1.46 \times 10^0$ | $4.93 \times 10^3$ |

### 2.1.5 $R_{1,3}$, $R_{4,6}$, $R_2$, $R_5$: ratio based contrast definition BFS

**Table S7.** Global iteration structures of the BFS procedure on the maximization of the ratio based contrast of the **26R** ⇋ **28R** switch substituting the $R_{1,3}$, $R_{3,6}$, $R_2$ and $R_5$ positions. The static hyper-Rayleigh scattering first hyperpolarizability values of the [26]hexaphyrins and [28]hexaphyrins are given in a.u.[a]

| Global iteration 1 | $R_{1,3}$_$R_2$_$R_{4,6}$_$R_5$ | $\beta_{HRS}$(28R) | $\beta_{HRS}$(26R) | contrast (ratio) | contrast (difference) |
|---|---|---|---|---|---|
| | $NH_2$_H_F_CN | $3.62 \times 10^3$ | $9.96 \times 10^3$ | $2.75 \times 10^0$ | $6.34 \times 10^3$ |
| | $NH_2$_F_F_CN | $5.33 \times 10^3$ | $9.25 \times 10^3$ | $1.73 \times 10^0$ | $3.91 \times 10^3$ |
| | $NH_2$_CN_F_CN | $1.73 \times 10^3$ | $2.42 \times 10^4$ | $1.40 \times 10^1$ | $2.25 \times 10^4$ |
| Substitution $R_2$ | $NH_2$_$CH_3$_F_CN | $4.46 \times 10^3$ | $9.14 \times 10^3$ | $2.05 \times 10^0$ | $4.67 \times 10^3$ |
| | $NH_2$_$NH_2$_F_CN | $9.06 \times 10^3$ | $5.73 \times 10^3$ | $6.32 \times 10^{-1}$ | $-3.34 \times 10^3$ |
| | $NH_2$_OH_F_CN | $7.30 \times 10^3$ | $7.39 \times 10^3$ | $1.01 \times 10^0$ | $8.81 \times 10^1$ |
| | $NH_2$_$NO_2$_F_CN | $4.63 \times 10^2$ | $2.25 \times 10^4$ | $4.86 \times 10^1$ | $2.20 \times 10^4$ |
| | H_$NO_2$_F_CN | $3.84 \times 10^3$ | $8.04 \times 10^3$ | $2.10 \times 10^0$ | $4.21 \times 10^3$ |
| | F_$NO_2$_F_CN | $1.52 \times 10^3$ | $1.27 \times 10^4$ | $8.37 \times 10^0$ | $1.12 \times 10^4$ |
| | CN_$NO_2$_F_CN | $7.94 \times 10^3$ | $3.27 \times 10^3$ | $4.12 \times 10^{-1}$ | $-4.67 \times 10^3$ |
| Substitution $R_{1,3}$ | $CH_3$_$NO_2$_F_CN | $3.61 \times 10^3$ | $1.15 \times 10^4$ | $3.19 \times 10^0$ | $7.91 \times 10^3$ |
| | $NH_2$_$NO_2$_F_CN | $4.63 \times 10^2$ | $2.25 \times 10^4$ | $4.86 \times 10^1$ | $2.20 \times 10^4$ |
| | OH_$NO_2$_F_CN | $7.27 \times 10^2$ | $1.59 \times 10^4$ | $2.19 \times 10^1$ | $1.52 \times 10^4$ |
| | $NO_2$_$NO_2$_F_CN | $4.31 \times 10^3$ | $4.28 \times 10^3$ | $9.92 \times 10^{-1}$ | $-3.32 \times 10^1$ |
| | $NH_2$_$NO_2$_H_CN | $6.75 \times 10^2$ | $1.57 \times 10^4$ | $2.32 \times 10^1$ | $1.50 \times 10^4$ |
| | $NH_2$_$NO_2$_F_CN | $4.63 \times 10^2$ | $2.25 \times 10^4$ | $4.86 \times 10^1$ | $2.20 \times 10^4$ |
| | $NH_2$_$NO_2$_CN_CN | $1.41 \times 10^4$ | $7.79 \times 10^3$ | $5.51 \times 10^{-1}$ | $-6.34 \times 10^3$ |
| Substitution $R_{4,6}$ | $NH_2$_$NO_2$_$CH_3$_CN | $7.04 \times 10^2$ | $1.88 \times 10^4$ | $2.67 \times 10^1$ | $1.81 \times 10^4$ |
| | $NH_2$_$NO_2$_$NH_2$_CN | $1.55 \times 10^3$ | $5.57 \times 10^4$ | $3.60 \times 10^1$ | $5.41 \times 10^4$ |
| | $NH_2$_$NO_2$_OH_CN | $6.45 \times 10^2$ | $4.93 \times 10^4$ | $7.65 \times 10^1$ | $4.87 \times 10^4$ |
| | $NH_2$_$NO_2$_$NO_2$_CN | $1.11 \times 10^4$ | $7.98 \times 10^3$ | $7.17 \times 10^{-1}$ | $-3.15 \times 10^3$ |
| | $NH_2$_$NO_2$_OH_H | $1.93 \times 10^3$ | $2.32 \times 10^4$ | $1.20 \times 10^1$ | $2.13 \times 10^4$ |
| | $NH_2$_$NO_2$_OH_F | $2.20 \times 10^3$ | $2.10 \times 10^4$ | $9.54 \times 10^0$ | $1.88 \times 10^4$ |
| | $NH_2$_$NO_2$_OH_CN | $6.45 \times 10^2$ | $4.93 \times 10^4$ | $7.65 \times 10^1$ | $4.87 \times 10^4$ |
| Substitution $R_5$ | $NH_2$_$NO_2$_OH_$CH_3$ | $2.02 \times 10^3$ | $2.14 \times 10^4$ | $1.06 \times 10^1$ | $1.94 \times 10^4$ |
| | $NH_2$_$NO_2$_OH_$NH_2$ | $2.62 \times 10^3$ | $1.40 \times 10^4$ | $5.33 \times 10^0$ | $1.13 \times 10^4$ |
| | $NH_2$_$NO_2$_OH_OH | $4.25 \times 10^3$ | $1.71 \times 10^4$ | $4.02 \times 10^0$ | $1.28 \times 10^4$ |
| | $NH_2$_$NO_2$_OH_$NO_2$ | $1.44 \times 10^3$ | $4.21 \times 10^4$ | $2.92 \times 10^1$ | $4.06 \times 10^4$ |

[a] These substitution patterns show a $\beta_{HRS}$**(28R)** below 10 a.u.. Their respective contrasts are computed considering a value of 0.001 for the $\beta_{HRS}$**(28R)**. For the difference definition the real value is used to compute the difference.

**Table S7.** Global iteration structures of the BFS procedure on the maximization of the ratio based contrast of the **26R** ⇋ **28R** switch substituting the $R_{1,3}$, $R_{3,6}$, $R_2$ and $R_5$ positions. The static hyper-Rayleigh scattering first hyperpolarizability values of the [26]hexaphyrins and [28]hexaphyrins are given in a.u.[a]

| Global iteration 2 | $R_{1,3}$_$R_2$_$R_{4,6}$_$R_5$ | $\beta_{HRS}$(28R) | $\beta_{HRS}$(26R) | contrast (ratio) | contrast (difference) |
|---|---|---|---|---|---|
| Substitution $R_{1,3}$ | H_$NO_2$_OH_CN | $2.95 \times 10^3$ | $9.13 \times 10^3$ | $3.10 \times 10^0$ | $6.18 \times 10^3$ |
| | F_$NO_2$_OH_CN | $2.58 \times 10^3$ | $1.38 \times 10^4$ | $5.37 \times 10^0$ | $1.13 \times 10^4$ |
| | CN_$NO_2$_OH_CN | $1.42 \times 10^4$ | $3.55 \times 10^3$ | $2.51 \times 10^{-1}$ | $-1.06 \times 10^4$ |
| | $CH_3$_$NO_2$_OH_CN | $1.59 \times 10^3$ | $1.23 \times 10^4$ | $7.74 \times 10^0$ | $1.07 \times 10^4$ |
| | $NH_2$_$NO_2$_OH_CN | $6.45 \times 10^2$ | $4.93 \times 10^4$ | $7.65 \times 10^1$ | $4.87 \times 10^4$ |
| | OH_$NO_2$_OH_CN | $8.08 \times 10^2$ | $3.32 \times 10^4$ | $4.11 \times 10^1$ | $3.24 \times 10^4$ |
| | $NO_2$_$NO_2$_OH_CN | $6.61 \times 10^3$ | $4.86 \times 10^3$ | $7.35 \times 10^{-1}$ | $-1.75 \times 10^3$ |
| Substitution $R_{4,6}$ | $NH_2$_$NO_2$_H_CN | $6.75 \times 10^2$ | $1.57 \times 10^4$ | $2.32 \times 10^1$ | $1.50 \times 10^4$ |
| | $NH_2$_$NO_2$_F_CN | $4.63 \times 10^2$ | $2.25 \times 10^4$ | $4.86 \times 10^1$ | $2.20 \times 10^4$ |
| | $NH_2$_$NO_2$_CN_CN | $1.41 \times 10^4$ | $7.79 \times 10^3$ | $5.51 \times 10^{-1}$ | $-6.34 \times 10^3$ |
| | $NH_2$_$NO_2$_$CH_3$_CN | $7.04 \times 10^2$ | $1.88 \times 10^4$ | $2.67 \times 10^1$ | $1.81 \times 10^4$ |
| | $NH_2$_$NO_2$_$NH_2$_CN | $1.55 \times 10^3$ | $5.57 \times 10^4$ | $3.60 \times 10^1$ | $5.41 \times 10^4$ |
| | $NH_2$_$NO_2$_OH_CN | $6.45 \times 10^2$ | $4.93 \times 10^4$ | $7.65 \times 10^1$ | $4.87 \times 10^4$ |
| | $NH_2$_$NO_2$_$NO_2$_CN | $1.11 \times 10^4$ | $7.98 \times 10^3$ | $7.17 \times 10^{-1}$ | $-3.15 \times 10^3$ |
| Substitution $R_5$ | $NH_2$_$NO_2$_OH_H | $1.93 \times 10^3$ | $2.32 \times 10^4$ | $1.20 \times 10^1$ | $2.13 \times 10^4$ |
| | $NH_2$_$NO_2$_OH_F | $2.20 \times 10^3$ | $2.10 \times 10^4$ | $9.54 \times 10^0$ | $1.88 \times 10^4$ |
| | $NH_2$_$NO_2$_OH_CN | $6.45 \times 10^2$ | $4.93 \times 10^4$ | $7.65 \times 10^1$ | $4.87 \times 10^4$ |
| | $NH_2$_$NO_2$_OH_$CH_3$ | $2.02 \times 10^3$ | $2.14 \times 10^4$ | $1.06 \times 10^1$ | $1.94 \times 10^4$ |
| | $NH_2$_$NO_2$_OH_$NH_2$ | $2.62 \times 10^3$ | $1.40 \times 10^4$ | $5.33 \times 10^0$ | $1.13 \times 10^4$ |
| | $NH_2$_$NO_2$_OH_OH | $4.25 \times 10^3$ | $1.71 \times 10^4$ | $4.02 \times 10^0$ | $1.28 \times 10^4$ |
| | $NH_2$_$NO_2$_OH_$NO_2$ | $1.44 \times 10^3$ | $4.21 \times 10^4$ | $2.92 \times 10^1$ | $4.06 \times 10^4$ |
| Substitution $R_2$ | $NH_2$_H_OH_CN | $1.71 \times 10^3$ | $2.44 \times 10^4$ | $1.42 \times 10^1$ | $2.27 \times 10^4$ |
| | $NH_2$_F_OH_CN | $4.90 \times 10^3$ | $2.29 \times 10^4$ | $4.67 \times 10^0$ | $1.80 \times 10^4$ |
| | $NH_2$_CN_OH_CN | $9.06 \times 10^2$ | $4.95 \times 10^4$ | $5.46 \times 10^1$ | $4.85 \times 10^4$ |
| | $NH_2$_$CH_3$_OH_CN | $2.48 \times 10^3$ | $2.28 \times 10^4$ | $9.22 \times 10^0$ | $2.03 \times 10^4$ |
| | $NH_2$_$NH_2$_OH_CN | $3.76 \times 10^3$ | $1.49 \times 10^4$ | $3.97 \times 10^0$ | $1.11 \times 10^4$ |
| | $NH_2$_OH_OH_CN | $9.37 \times 10^3$ | $1.89 \times 10^4$ | $2.01 \times 10^0$ | $9.49 \times 10^3$ |
| | $NH_2$_$NO_2$_OH_CN | $6.45 \times 10^2$ | $4.93 \times 10^4$ | $7.65 \times 10^1$ | $4.87 \times 10^4$ |

[a] These substitution patterns show a $\beta_{HRS}$(**28R**) below 10 a.u.. Their respective contrasts are computed considering a value of 0.001 for the $\beta_{HRS}$(**28R**). For the difference definition the real value is used to compute the difference.

### 2.1.6 $R_{1,3}$, $R_{4,6}$, $R_2$, $R_5$: difference based contrast definition BFS

**Table S8.** Global iteration structures of the BFS procedure on the maximization of the difference based contrast of the **26R** ⇋ **28R** switch substituting the $R_{1,3}$, $R_{3,6}$, $R_2$ and $R_5$ positions. The static hyper-Rayleigh scattering first hyperpolarizability values of the [26]hexaphyrins and [28]hexaphyrins are given in a.u..

| Global iteration 1 | $R_{1,3}$_$R_2$_$R_{4,6}$_$R_5$ | $\beta_{HRS}$(28R) | $\beta_{HRS}$(26R) | contrast (ratio) | contrast (difference) |
|---|---|---|---|---|---|
| Substitution $R_{4,6}$ | $NH_2$_CN_H_$NO_2$ | $6.75 \times 10^2$ | $1.57 \times 10^4$ | $2.32 \times 10^1$ | $1.50 \times 10^4$ |
| | $NH_2$_CN_F_$NO_2$ | $4.63 \times 10^2$ | $2.25 \times 10^4$ | $4.86 \times 10^1$ | $2.20 \times 10^4$ |
| | $NH_2$_CN_CN_$NO_2$ | $1.41 \times 10^4$ | $7.79 \times 10^3$ | $5.51 \times 10^{-1}$ | $-6.34 \times 10^3$ |
| | $NH_2$_CN_$CH_3$_$NO_2$ | $7.04 \times 10^2$ | $1.88 \times 10^4$ | $2.67 \times 10^1$ | $1.81 \times 10^4$ |
| | $NH_2$_CN_$NH_2$_$NO_2$ | $1.55 \times 10^3$ | $5.57 \times 10^4$ | $3.60 \times 10^1$ | $5.41 \times 10^4$ |
| | $NH_2$_CN_OH_$NO_2$ | $6.45 \times 10^2$ | $4.93 \times 10^4$ | $7.65 \times 10^1$ | $4.87 \times 10^4$ |
| | $NH_2$_CN_$NO_2$_$NO_2$ | $1.11 \times 10^4$ | $7.98 \times 10^3$ | $7.17 \times 10^{-1}$ | $-3.15 \times 10^3$ |
| Substitution $R_2$ | $NH_2$_H_$NH_2$_$NO_2$ | $4.17 \times 10^3$ | $2.74 \times 10^4$ | $6.56 \times 10^0$ | $2.32 \times 10^4$ |
| | $NH_2$_F_$NH_2$_$NO_2$ | $4.46 \times 10^3$ | $2.52 \times 10^4$ | $5.67 \times 10^0$ | $2.08 \times 10^4$ |
| | $NH_2$_CN_$NH_2$_$NO_2$ | $1.55 \times 10^3$ | $5.57 \times 10^4$ | $3.60 \times 10^1$ | $5.41 \times 10^4$ |
| | $NH_2$_$CH_3$_$NH_2$_$NO_2$ | $3.08 \times 10^3$ | $2.46 \times 10^4$ | $7.98 \times 10^0$ | $2.15 \times 10^4$ |
| | $NH_2$_$NH_2$_$NH_2$_$NO_2$ | $8.34 \times 10^3$ | $1.47 \times 10^4$ | $1.77 \times 10^0$ | $6.41 \times 10^3$ |
| | $NH_2$_OH_$NH_2$_$NO_2$ | $6.53 \times 10^3$ | $1.99 \times 10^4$ | $3.04 \times 10^0$ | $1.33 \times 10^4$ |
| | $NH_2$_$NO_2$_$NH_2$_$NO_2$ | $7.07 \times 10^2$ | $5.94 \times 10^4$ | $8.41 \times 10^1$ | $5.87 \times 10^4$ |
| Substitution $R_{1,3}$ | H_$NO_2$_$NH_2$_$NO_2$ | $2.15 \times 10^3$ | $1.17 \times 10^4$ | $5.46 \times 10^0$ | $9.60 \times 10^3$ |
| | F_$NO_2$_$NH_2$_$NO_2$ | $2.05 \times 10^3$ | $1.69 \times 10^4$ | $8.28 \times 10^0$ | $1.49 \times 10^4$ |
| | CN_$NO_2$_$NH_2$_$NO_2$ | $1.37 \times 10^4$ | $5.42 \times 10^3$ | $3.94 \times 10^{-1}$ | $-8.33 \times 10^3$ |
| | $CH_3$_$NO_2$_$NH_2$_$NO_2$ | $1.10 \times 10^3$ | $1.48 \times 10^4$ | $1.34 \times 10^1$ | $1.37 \times 10^4$ |
| | $NH_2$_$NO_2$_$NH_2$_$NO_2$ | $7.07 \times 10^2$ | $5.94 \times 10^4$ | $8.41 \times 10^1$ | $5.87 \times 10^4$ |
| | OH_$NO_2$_$NH_2$_$NO_2$ | $4.41 \times 10^2$ | $4.19 \times 10^4$ | $9.52 \times 10^1$ | $4.15 \times 10^4$ |
| | $NO_2$_$NO_2$_$NH_2$_$NO_2$ | $6.94 \times 10^3$ | $6.75 \times 10^3$ | $9.73 \times 10^{-1}$ | $-1.84 \times 10^2$ |
| Substitution $R_5$ | $NH_2$_$NO_2$_$NH_2$_H | $3.85 \times 10^3$ | $2.65 \times 10^4$ | $6.88 \times 10^0$ | $2.26 \times 10^4$ |
| | $NH_2$_$NO_2$_$NH_2$_F | $4.50 \times 10^3$ | $2.42 \times 10^4$ | $5.37 \times 10^0$ | $1.97 \times 10^4$ |
| | $NH_2$_$NO_2$_$NH_2$_CN | $1.82 \times 10^3$ | $5.63 \times 10^4$ | $3.09 \times 10^1$ | $5.45 \times 10^4$ |
| | $NH_2$_$NO_2$_$NH_2$_$CH_3$ | $3.05 \times 10^3$ | $2.39 \times 10^4$ | $7.85 \times 10^0$ | $2.09 \times 10^4$ |
| | $NH_2$_$NO_2$_$NH_2$_$NH_2$ | $8.31 \times 10^3$ | $1.40 \times 10^4$ | $1.69 \times 10^0$ | $5.74 \times 10^3$ |
| | $NH_2$_$NO_2$_$NH_2$_OH | $6.44 \times 10^3$ | $1.87 \times 10^4$ | $2.90 \times 10^0$ | $1.22 \times 10^4$ |
| | $NH_2$_$NO_2$_$NH_2$_$NO_2$ | $7.07 \times 10^2$ | $5.94 \times 10^4$ | $8.41 \times 10^1$ | $5.87 \times 10^4$ |

**Table S8.** Global iteration structures of the BFS procedure on the maximization of the difference based contrast of the **26R** ⇋ **28R** switch substituting the $R_{1,3}$, $R_{3,6}$, $R_2$ and $R_5$ positions. The static hyper-Rayleigh scattering first hyperpolarizability values of the [26]hexaphyrins and [28]hexaphyrins are given in a.u..

| **Global iteration 2** | **$R_{1,3}$_$R_2$_$R_{4,6}$_$R_5$** | **$\beta_{HRS}$(28R)** | **$\beta_{HRS}$(26R)** | **contrast (ratio)** | **contrast (difference)** |
|---|---|---|---|---|---|
| Substitution $R_5$ | $NH_2$_$NO_2$_$NH_2$_H | $3.85 \times 10^3$ | $2.65 \times 10^4$ | $6.88 \times 10^0$ | $2.26 \times 10^4$ |
| | $NH_2$_$NO_2$_$NH_2$_F | $4.50 \times 10^3$ | $2.42 \times 10^4$ | $5.37 \times 10^0$ | $1.97 \times 10^4$ |
| | $NH_2$_$NO_2$_$NH_2$_CN | $1.82 \times 10^3$ | $5.63 \times 10^4$ | $3.09 \times 10^1$ | $5.45 \times 10^4$ |
| | $NH_2$_$NO_2$_$NH_2$_$CH_3$ | $3.05 \times 10^3$ | $2.39 \times 10^4$ | $7.85 \times 10^0$ | $2.09 \times 10^4$ |
| | $NH_2$_$NO_2$_$NH_2$_$NH_2$ | $8.31 \times 10^3$ | $1.40 \times 10^4$ | $1.69 \times 10^0$ | $5.74 \times 10^3$ |
| | $NH_2$_$NO_2$_$NH_2$_OH | $6.44 \times 10^3$ | $1.87 \times 10^4$ | $2.90 \times 10^0$ | $1.22 \times 10^4$ |
| | $NH_2$_$NO_2$_$NH_2$_$NO_2$ | $7.07 \times 10^2$ | $5.94 \times 10^4$ | $8.41 \times 10^1$ | $5.87 \times 10^4$ |
| Substitution $R_2$ | $NH_2$_H_$NH_2$_$NO_2$ | $4.17 \times 10^3$ | $2.74 \times 10^4$ | $6.56 \times 10^0$ | $2.32 \times 10^4$ |
| | $NH_2$_F_$NH_2$_$NO_2$ | $4.46 \times 10^3$ | $2.52 \times 10^4$ | $5.67 \times 10^0$ | $2.08 \times 10^4$ |
| | $NH_2$_CN_$NH_2$_$NO_2$ | $1.55 \times 10^3$ | $5.57 \times 10^4$ | $3.60 \times 10^1$ | $5.41 \times 10^4$ |
| | $NH_2$_$NH_2$_$CH_3$_$NO_2$ | $3.08 \times 10^3$ | $2.46 \times 10^4$ | $7.98 \times 10^0$ | $2.15 \times 10^4$ |
| | $NH_2$_$NH_2$_$NH_2$_$NO_2$ | $8.34 \times 10^3$ | $1.47 \times 10^4$ | $1.77 \times 10^0$ | $6.41 \times 10^3$ |
| | $NH_2$_OH_$NH_2$_$NO_2$ | $6.53 \times 10^3$ | $1.99 \times 10^4$ | $3.04 \times 10^0$ | $1.33 \times 10^4$ |
| | $NH_2$_$NO_2$_$NH_2$_$NO_2$ | $7.07 \times 10^2$ | $5.94 \times 10^4$ | $8.41 \times 10^1$ | $5.87 \times 10^4$ |
| Substitution $R_{1,3}$ | H_$NO_2$_$NH_2$_$NO_2$ | $2.15 \times 10^3$ | $1.17 \times 10^4$ | $5.46 \times 10^0$ | $9.60 \times 10^3$ |
| | F_$NO_2$_$NH_2$_$NO_2$ | $2.05 \times 10^3$ | $1.69 \times 10^4$ | $8.28 \times 10^0$ | $1.49 \times 10^4$ |
| | CN_$NO_2$_$NH_2$_$NO_2$ | $1.37 \times 10^4$ | $5.42 \times 10^3$ | $3.94 \times 10^{-1}$ | $-8.33 \times 10^3$ |
| | $CH_3$_$NO_2$_$NH_2$_$NO_2$ | $1.10 \times 10^3$ | $1.48 \times 10^4$ | $1.34 \times 10^1$ | $1.37 \times 10^4$ |
| | $NH_2$_$NO_2$_$NH_2$_$NO_2$ | $7.07 \times 10^2$ | $5.94 \times 10^4$ | $8.41 \times 10^1$ | $5.87 \times 10^4$ |
| | OH_$NO_2$_$NH_2$_$NO_2$ | $4.41 \times 10^2$ | $4.19 \times 10^4$ | $9.52 \times 10^1$ | $4.15 \times 10^4$ |
| | $NO_2$_$NO_2$_$NH_2$_$NO_2$ | $6.94 \times 10^3$ | $6.75 \times 10^3$ | $9.73 \times 10^{-1}$ | $-1.84 \times 10^2$ |
| Substitution $R_{4,6}$ | $NH_2$_$NO_2$_H_$NO_2$ | $2.43 \times 10^3$ | $1.22 \times 10^4$ | $5.03 \times 10^0$ | $9.79 \times 10^3$ |
| | $NH_2$_$NO_2$_F_$NO_2$ | $2.29 \times 10^3$ | $1.73 \times 10^4$ | $7.57 \times 10^0$ | $1.50 \times 10^4$ |
| | $NH_2$_$NO_2$_CN_$NO_2$ | $1.53 \times 10^4$ | $5.72 \times 10^3$ | $3.75 \times 10^{-1}$ | $-9.54 \times 10^3$ |
| | $NH_2$_$NO_2$_$CH_3$_$NO_2$ | $1.53 \times 10^3$ | $1.45 \times 10^4$ | $9.52 \times 10^0$ | $1.30 \times 10^4$ |
| | $NH_2$_$NO_2$_$NH_2$_$NO_2$ | $7.07 \times 10^2$ | $5.94 \times 10^4$ | $8.41 \times 10^1$ | $5.87 \times 10^4$ |
| | $NH_2$_$NO_2$_OH_$NO_2$ | $1.44 \times 10^3$ | $4.21 \times 10^4$ | $2.92 \times 10^1$ | $4.06 \times 10^4$ |
| | $NH_2$_$NO_2$_$NO_2$_$NO_2$ | $1.10 \times 10^4$ | $6.37 \times 10^3$ | $5.76 \times 10^{-1}$ | $-4.68 \times 10^3$ |

## 2.2 Inverse Design: 28R $\leftrightharpoons$ 28M switch

### 2.2.1 $R_{1,4}$, $R_{2,5}$ and $R_{3,6}$ positions: ratio based contrast definition BFS

**Table S9.** Global iteration structures of the BFS procedure on the maximization of the difference based contrast of the **28M** $\leftrightharpoons$ **28R** switch substituting the $R_{1,4}$, $R_{2,5}$ and $R_{3,6}$ positions. The static hyper-Rayleigh scattering first hyperpolarizability values of the [28]hexaphyrins are given in a.u.[a]

| **Global iteration 1** | **$R_{1,4}$_$R_{2,5}$_$R_{3,6}$** | **$\beta_{HRS}$(28R)** | **$\beta_{HRS}$(28M)** | **contrast (ratio)** | **contrast (difference)** |
|---|---|---|---|---|---|
| | $NH_2$_H _F | $1.00 \times 10^{-3}$ | $6.96 \times 10^{2}$ | $6.96 \times 10^{5}$ | $6.95 \times 10^{2}$ |
| | $NH_2$_F_F | $1.00 \times 10^{-3}$ | $1.42 \times 10^{3}$ | $1.42 \times 10^{6}$ | $1.42 \times 10^{3}$ |
| | $NH_2$_CN_F | $1.00 \times 10^{-3}$ | $7.89 \times 10^{2}$ | $7.89 \times 10^{5}$ | $7.88 \times 10^{2}$ |
| Substitution $R_{2,5}$ | $NH_2$_$CH_3$_F | $1.00 \times 10^{-3}$ | $1.24 \times 10^{3}$ | $1.24 \times 10^{6}$ | $1.24 \times 10^{3}$ |
| | $NH_2$_$NH_2$_F | $1.00 \times 10^{-3}$ | $2.08 \times 10^{3}$ | $2.08 \times 10^{6}$ | $2.08 \times 10^{3}$ |
| | $NH_2$_OH_F | $1.91 \times 10^{0}$ | $2.59 \times 10^{3}$ | $2.59 \times 10^{6}$ | $2.59 \times 10^{3}$ |
| | $NH_2$_$NO_2$_F | $1.84 \times 10^{2}$ | $1.78 \times 10^{3}$ | $9.68 \times 10^{0}$ | $1.60 \times 10^{3}$ |
| | $NH_2$_OH_H | $1.00 \times 10^{-3}$ | $2.38 \times 10^{3}$ | $2.38 \times 10^{6}$ | $2.38 \times 10^{3}$ |
| | $NH_2$_OH_F | $1.00 \times 10^{-3}$ | $2.59 \times 10^{3}$ | $2.59 \times 10^{6}$ | $2.59 \times 10^{3}$ |
| | $NH_2$_OH_CN | $2.48 \times 10^{0}$ | $2.93 \times 10^{3}$ | $2.93 \times 10^{6}$ | $2.93 \times 10^{3}$ |
| Substitution $R_{3,6}$ | $NH_2$_OH_$CH_3$ | $1.00 \times 10^{-3}$ | $2.37 \times 10^{3}$ | $2.37 \times 10^{6}$ | $2.37 \times 10^{3}$ |
| | $NH_2$_OH_$NH_2$ | $1.00 \times 10^{-3}$ | $2.64 \times 10^{3}$ | $2.64 \times 10^{6}$ | $2.64 \times 10^{3}$ |
| | $NH_2$_OH_OH | $1.00 \times 10^{-3}$ | $2.70 \times 10^{3}$ | $1.85 \times 10^{0}$ | $1.24 \times 10^{3}$ |
| | $NH_2$_OH_$NO_2$ | $1.00 \times 10^{-3}$ | $2.60 \times 10^{3}$ | $2.60 \times 10^{6}$ | $2.59 \times 10^{3}$ |
| | H_OH_CN | $1.00 \times 10^{-3}$ | $1.69 \times 10^{3}$ | $1.69 \times 10^{6}$ | $1.69 \times 10^{3}$ |
| | F_OH_CN | $1.00 \times 10^{-3}$ | $1.76 \times 10^{3}$ | $1.76 \times 10^{6}$ | $1.76 \times 10^{3}$ |
| | CN_OH_CN | $5.50 \times 10^{2}$ | $1.60 \times 10^{3}$ | $2.91 \times 10^{0}$ | $1.05 \times 10^{3}$ |
| Substitution $R_{1,4}$ | $CH_3$_OH_CN | $9.47 \times 10^{2}$ | $1.86 \times 10^{3}$ | $1.96 \times 10^{0}$ | $9.08 \times 10^{2}$ |
| | $NH_2$_OH_CN | $1.00 \times 10^{-3}$ | $2.93 \times 10^{3}$ | $2.93 \times 10^{6}$ | $2.93 \times 10^{3}$ |
| | OH_OH_CN | $1.14 \times 10^{3}$ | $2.32 \times 10^{3}$ | $2.05 \times 10^{0}$ | $1.19 \times 10^{3}$ |
| | $NO_2$_OH_CN | $1.18 \times 10^{3}$ | $2.38 \times 10^{3}$ | $2.02 \times 10^{0}$ | $1.20 \times 10^{3}$ |
| **Global iteration 2** | **$R_{1,4}$_$R_{2,5}$_$R_{3,6}$** | **$\beta_{HRS}$(28R)** | **$\beta_{HRS}$(28M)** | **contrast (ratio)** | **contrast (difference)** |
| | $NH_2$_H_CN | $1.00 \times 10^{-3}$ | $1.69 \times 10^{3}$ | $1.69 \times 10^{6}$ | $1.69 \times 10^{3}$ |
| | $NH_2$_F_CN | $1.00 \times 10^{-3}$ | $2.14 \times 10^{3}$ | $2.14 \times 10^{6}$ | $2.14 \times 10^{3}$ |
| | $NH_2$_CN_CN | $1.00 \times 10^{-3}$ | $1.65 \times 10^{3}$ | $1.65 \times 10^{6}$ | $1.65 \times 10^{3}$ |
| Substitution $R_{2,5}$ | $NH_2$_$CH_3$_CN | $1.00 \times 10^{-3}$ | $1.99 \times 10^{3}$ | $1.99 \times 10^{6}$ | $1.99 \times 10^{3}$ |
| | $NH_2$_$NH_2$_CN | $1.00 \times 10^{-3}$ | $3.12 \times 10^{3}$ | $3.12 \times 10^{6}$ | $3.12 \times 10^{3}$ |
| | $NH_2$_OH_CN | $1.00 \times 10^{-3}$ | $2.93 \times 10^{3}$ | $2.93 \times 10^{6}$ | $2.93 \times 10^{3}$ |
| | $NH_2$_$NO_2$_CN | $1.34 \times 10^{2}$ | $2.16 \times 10^{3}$ | $1.61 \times 10^{1}$ | $2.02 \times 10^{3}$ |
| | $NH_2$_$NH_2$_H | $1.00 \times 10^{-3}$ | $1.64 \times 10^{3}$ | $1.64 \times 10^{6}$ | $1.64 \times 10^{3}$ |
| | $NH_2$_$NH_2$_F | $1.00 \times 10^{-3}$ | $2.08 \times 10^{3}$ | $2.08 \times 10^{6}$ | $2.08 \times 10^{3}$ |
| | $NH_2$_$NH_2$_CN | $1.00 \times 10^{-3}$ | $3.12 \times 10^{3}$ | $3.12 \times 10^{6}$ | $3.12 \times 10^{3}$ |
| Substitution $R_{3,6}$ | $NH_2$_$NH_2$_$CH_3$ | $2.55 \times 10^{1}$ | $2.05 \times 10^{3}$ | $8.03 \times 10^{1}$ | $2.02 \times 10^{3}$ |
| | $NH_2$_$NH_2$_$NH_2$ | $2.70 \times 10^{3}$ | $2.21 \times 10^{3}$ | $8.19 \times 10^{-1}$ | $-4.90 \times 10^{2}$ |
| | $NH_2$_$NH_2$_OH | $1.46 \times 10^{3}$ | $2.05 \times 10^{3}$ | $1.41 \times 10^{0}$ | $5.95 \times 10^{2}$ |
| | $NH_2$_$NH_2$_$NO_2$ | $1.00 \times 10^{-3}$ | $2.54 \times 10^{3}$ | $2.54 \times 10^{6}$ | $2.54 \times 10^{3}$ |
| | H_$NH_2$_CN | $1.00 \times 10^{-3}$ | $1.82 \times 10^{3}$ | $1.82 \times 10^{6}$ | $1.82 \times 10^{3}$ |
| | F_$NH_2$_CN | $1.00 \times 10^{-3}$ | $1.91 \times 10^{3}$ | $1.91 \times 10^{6}$ | $1.91 \times 10^{3}$ |
| | CN_$NH_2$_CN | $9.08 \times 10^{2}$ | $1.87 \times 10^{3}$ | $2.06 \times 10^{0}$ | $9.60 \times 10^{2}$ |
| Substitution $R_{1,4}$ | $CH_3$_$NH_2$_CN | $1.15 \times 10^{3}$ | $1.97 \times 10^{3}$ | $1.72 \times 10^{0}$ | $8.25 \times 10^{2}$ |
| | $NH_2$_$NH_2$_CN | $1.00 \times 10^{-3}$ | $3.12 \times 10^{3}$ | $3.12 \times 10^{6}$ | $3.12 \times 10^{3}$ |
| | OH_$NH_2$_CN | $1.08 \times 10^{3}$ | $2.23 \times 10^{3}$ | $2.06 \times 10^{0}$ | $1.15 \times 10^{3}$ |
| | $NO_2$_$NH_2$_CN | $1.39 \times 10^{3}$ | $2.44 \times 10^{3}$ | $1.76 \times 10^{0}$ | $1.05 \times 10^{3}$ |

[a] These substitution patterns show a $\beta_{HRS}$**(28R)** below 10 a.u.. Their respective contrasts are computed considering a value of 0.001 for the $\beta_{HRS}$**(28R)**. For the difference definition the real value is used to compute the difference.

**Table S9.** Global iteration structures of the BFS procedure on the maximization of the difference based contrast of the **28M** ⇆ **28R** switch substituting the $R_{1,4}$, $R_{2,5}$ and $R_{3,6}$ positions. The static hyper-Rayleigh scattering first hyperpolarizability values of the [28]hexaphyrins are given in a.u. [a]

| Global iteration 3 | $R_{1,4}$_$R_{2,5}$_$R_{3,6}$ | $\beta_{HRS}$(28R) | $\beta_{HRS}$(28M) | contrast (ratio) | contrast (difference) |
|---|---|---|---|---|---|
| Substitution $R_{3,6}$ | $NH_2$_$NH_2$_H | $1.00 \times 10^{-3}$ | $1.64 \times 10^{3}$ | $1.64 \times 10^{6}$ | $1.64 \times 10^{3}$ |
| | $NH_2$_$NH_2$_F | $1.00 \times 10^{-3}$ | $2.08 \times 10^{3}$ | $2.08 \times 10^{6}$ | $2.08 \times 10^{3}$ |
| | $NH_2$_$NH_2$_CN | $1.00 \times 10^{-3}$ | $3.12 \times 10^{3}$ | $3.12 \times 10^{6}$ | $3.12 \times 10^{3}$ |
| | $NH_2$_$NH_2$_$CH_3$ | $2.55 \times 10^{1}$ | $2.05 \times 10^{3}$ | $8.03 \times 10^{1}$ | $2.02 \times 10^{3}$ |
| | $NH_2$_$NH_2$_$NH_2$ | $2.70 \times 10^{3}$ | $2.21 \times 10^{3}$ | $8.19 \times 10^{-1}$ | $-4.90 \times 10^{2}$ |
| | $NH_2$_$NH_2$_OH | $1.46 \times 10^{3}$ | $2.05 \times 10^{3}$ | $1.41 \times 10^{0}$ | $5.95 \times 10^{2}$ |
| | $NH_2$_$NH_2$_$NO_2$ | $1.00 \times 10^{-3}$ | $2.54 \times 10^{3}$ | $2.54 \times 10^{6}$ | $2.54 \times 10^{3}$ |
| Substitution $R_{2,5}$ | $NH_2$_H_CN | $1.00 \times 10^{-3}$ | $1.69 \times 10^{3}$ | $1.69 \times 10^{6}$ | $1.69 \times 10^{3}$ |
| | $NH_2$_F_CN | $1.00 \times 10^{-3}$ | $2.14 \times 10^{3}$ | $2.14 \times 10^{6}$ | $2.14 \times 10^{3}$ |
| | $NH_2$_CN_CN | $1.00 \times 10^{-3}$ | $1.65 \times 10^{3}$ | $1.65 \times 10^{6}$ | $1.65 \times 10^{3}$ |
| | $NH_2$_$CH_3$_CN | $1.00 \times 10^{-3}$ | $1.99 \times 10^{3}$ | $1.99 \times 10^{6}$ | $1.99 \times 10^{3}$ |
| | $NH_2$_$NH_2$_CN | $1.00 \times 10^{-3}$ | $3.12 \times 10^{3}$ | $3.12 \times 10^{6}$ | $3.12 \times 10^{3}$ |
| | $NH_2$_OH_CN | $1.00 \times 10^{-3}$ | $2.93 \times 10^{3}$ | $2.93 \times 10^{6}$ | $2.93 \times 10^{3}$ |
| | $NH_2$_$NO_2$_CN | $1.34 \times 10^{2}$ | $2.16 \times 10^{3}$ | $1.61 \times 10^{1}$ | $2.02 \times 10^{3}$ |
| Substitution $R_{1,4}$ | H_$NH_2$_CN | $1.00 \times 10^{-3}$ | $1.82 \times 10^{3}$ | $1.82 \times 10^{6}$ | $1.82 \times 10^{3}$ |
| | F_$NH_2$_CN | $1.00 \times 10^{-3}$ | $1.91 \times 10^{3}$ | $1.91 \times 10^{6}$ | $1.91 \times 10^{3}$ |
| | CN_$NH_2$_CN | $9.08 \times 10^{2}$ | $1.87 \times 10^{3}$ | $2.06 \times 10^{0}$ | $9.60 \times 10^{2}$ |
| | $CH_3$_$NH_2$_CN | $1.15 \times 10^{3}$ | $1.97 \times 10^{3}$ | $1.72 \times 10^{0}$ | $8.25 \times 10^{2}$ |
| | $NH_2$_$NH_2$_CN | $1.00 \times 10^{-3}$ | $3.12 \times 10^{3}$ | $3.12 \times 10^{6}$ | $3.12 \times 10^{3}$ |
| | OH_$NH_2$_CN | $1.08 \times 10^{3}$ | $2.23 \times 10^{3}$ | $2.06 \times 10^{0}$ | $1.15 \times 10^{3}$ |
| | $NO_2$_$NH_2$_CN | $1.39 \times 10^{3}$ | $2.44 \times 10^{3}$ | $1.76 \times 10^{0}$ | $1.05 \times 10^{3}$ |

[a] These substitution patterns show a $\beta_{HRS}$**(28R)** below 10 a.u.. Their respective contrasts are computed considering a value of 0.001 for the $\beta_{HRS}$**(28R)**. For the difference definition the real value is used to compute the difference.

### 2.2.2 $R_{1,4}$, $R_{2,5}$ and $R_{3,6}$ positions: difference based contrast definition BFS

**Table S10.** Global iteration structures of the additional BFS procedure on the maximization of the difference based contrast of the **28M** ⇋ **28R** switch substituting the $R_{1,4}$, $R_{2,5}$ and $R_{3,6}$ positions. The static hyper-Rayleigh scattering first hyperpolarizability values of the [28]hexaphyrins are given in a.u..

| **Global iteration 1** | $R_{1,4}$_$R_{2,5}$_$R_{3,6}$ | $\beta_{HRS}$(28R) | $\beta_{HRS}$(28M) | contrast (ratio) | contrast (difference) |
|---|---|---|---|---|---|
| Substitution $R_{1,4}$ | H_CN_CN | $6.05 \times 10^{-2}$ | $1.60 \times 10^{3}$ | $1.60 \times 10^{6}$ | $1.60 \times 10^{3}$ |
| | F_CN_CN | $0.00 \times 10^{0}$ | $1.15 \times 10^{3}$ | $1.15 \times 10^{6}$ | $1.15 \times 10^{3}$ |
| | CN_CN_CN | $1.17 \times 10^{3}$ | $1.54 \times 10^{3}$ | $1.31 \times 10^{0}$ | $3.68 \times 10^{2}$ |
| | $CH_3$_CN_CN | $4.98 \times 10^{0}$ | $1.59 \times 10^{3}$ | $1.59 \times 10^{6}$ | $1.58 \times 10^{3}$ |
| | $NH_2$_CN_CN | $2.31 \times 10^{-2}$ | $1.65 \times 10^{3}$ | $1.65 \times 10^{6}$ | $1.65 \times 10^{3}$ |
| | OH_CN_CN | $4.40 \times 10^{2}$ | $1.18 \times 10^{3}$ | $2.68 \times 10^{0}$ | $7.38 \times 10^{2}$ |
| | $NO_2$_CN_CN | $1.61 \times 10^{3}$ | $2.25 \times 10^{3}$ | $1.39 \times 10^{0}$ | $6.33 \times 10^{2}$ |
| Substitution $R_{2,5}$ | $NH_2$_CN_H | $3.42 \times 10^{-2}$ | $8.22 \times 10^{2}$ | $8.22 \times 10^{5}$ | $8.21 \times 10^{2}$ |
| | $NH_2$_CN_F | $1.41 \times 10^{0}$ | $7.89 \times 10^{2}$ | $7.89 \times 10^{5}$ | $7.88 \times 10^{2}$ |
| | $NH_2$_CN_CN | $2.31 \times 10^{-2}$ | $1.65 \times 10^{3}$ | $1.65 \times 10^{6}$ | $1.65 \times 10^{3}$ |
| | $NH_2$_CN_$CH_3$ | $7.40 \times 10^{-1}$ | $9.05 \times 10^{2}$ | $9.05 \times 10^{5}$ | $9.05 \times 10^{2}$ |
| | $NH_2$_CN_$NH_2$ | $1.49 \times 10^{-1}$ | $2.04 \times 10^{3}$ | $2.04 \times 10^{6}$ | $2.04 \times 10^{3}$ |
| | $NH_2$_CN_OH | $5.86 \times 10^{2}$ | $1.10 \times 10^{3}$ | $1.88 \times 10^{0}$ | $5.13 \times 10^{2}$ |
| | $NH_2$_CN_$NO_2$ | $6.43 \times 10^{-1}$ | $3.06 \times 10^{3}$ | $3.06 \times 10^{6}$ | $3.06 \times 10^{3}$ |
| Substitution $R_{3,6}$ | $NH_2$_H_$NO_2$ | $1.43 \times 10^{3}$ | $2.92 \times 10^{3}$ | $2.05 \times 10^{0}$ | $1.50 \times 10^{3}$ |
| | $NH_2$_F_$NO_2$ | $2.16 \times 10^{3}$ | $2.53 \times 10^{3}$ | $1.17 \times 10^{0}$ | $3.70 \times 10^{2}$ |
| | $NH_2$_CN_$NO_2$ | $6.43 \times 10^{-1}$ | $3.06 \times 10^{3}$ | $3.06 \times 10^{6}$ | $3.06 \times 10^{3}$ |
| | $NH_2$_$CH_3$_$NO_2$ | $2.47 \times 10^{3}$ | $2.96 \times 10^{3}$ | $1.20 \times 10^{0}$ | $4.94 \times 10^{2}$ |
| | $NH_2$_$NH_2$_$NO_2$ | $1.20 \times 10^{0}$ | $2.54 \times 10^{3}$ | $2.54 \times 10^{6}$ | $2.54 \times 10^{3}$ |
| | $NH_2$_OH_$NO_2$ | $1.58 \times 10^{0}$ | $2.60 \times 10^{3}$ | $2.60 \times 10^{6}$ | $2.59 \times 10^{3}$ |
| | $NH_2$_$NO_2$_$NO_2$ | $1.56 \times 10^{3}$ | $3.09 \times 10^{3}$ | $1.98 \times 10^{0}$ | $1.53 \times 10^{3}$ |
| **Global iteration 2** | **$R_{1,4}$_$R_{2,5}$_$R_{3,6}$** | **$\beta_{HRS}$(28R)** | **$\beta_{HRS}$(28M)** | **contrast (ratio)** | **contrast (difference)** |
| Substitution $R_{3,6}$ | $NH_2$_H_$NO_2$ | $1.43 \times 10^{3}$ | $2.92 \times 10^{3}$ | $2.05 \times 10^{0}$ | $1.50 \times 10^{3}$ |
| | $NH_2$_F_$NO_2$ | $2.16 \times 10^{3}$ | $2.53 \times 10^{3}$ | $1.17 \times 10^{0}$ | $3.70 \times 10^{2}$ |
| | $NH_2$_CN_$NO_2$ | $6.43 \times 10^{-1}$ | $3.06 \times 10^{3}$ | $3.06 \times 10^{6}$ | $3.06 \times 10^{3}$ |
| | $NH_2$_$CH_3$_$NO_2$ | $2.47 \times 10^{3}$ | $2.96 \times 10^{3}$ | $1.20 \times 10^{0}$ | $4.94 \times 10^{2}$ |
| | $NH_2$_$NH_2$_$NO_2$ | $1.20 \times 10^{0}$ | $2.54 \times 10^{3}$ | $2.54 \times 10^{6}$ | $2.54 \times 10^{3}$ |
| | $NH_2$_OH_$NO_2$ | $1.58 \times 10^{0}$ | $2.60 \times 10^{3}$ | $2.60 \times 10^{6}$ | $2.59 \times 10^{3}$ |
| | $NH_2$_$NO_2$_$NO_2$ | $1.56 \times 10^{3}$ | $3.09 \times 10^{3}$ | $1.98 \times 10^{0}$ | $1.53 \times 10^{3}$ |
| Substitution $R_{1,4}$ | H_CN_$NO_2$ | $1.05 \times 10^{0}$ | $2.08 \times 10^{3}$ | $2.08 \times 10^{6}$ | $2.08 \times 10^{3}$ |
| | F_CN_$NO_2$ | $6.12 \times 10^{2}$ | $1.91 \times 10^{3}$ | $3.12 \times 10^{0}$ | $1.30 \times 10^{3}$ |
| | CN_CN_$NO_2$ | $7.27 \times 10^{1}$ | $1.43 \times 10^{3}$ | $1.96 \times 10^{1}$ | $1.36 \times 10^{3}$ |
| | $CH_3$_CN_$NO_2$ | $2.50 \times 10^{0}$ | $2.20 \times 10^{3}$ | $2.20 \times 10^{6}$ | $2.20 \times 10^{3}$ |
| | $NH_2$_CN_$NO_2$ | $6.43 \times 10^{-1}$ | $3.06 \times 10^{3}$ | $3.06 \times 10^{6}$ | $3.06 \times 10^{3}$ |
| | OH_CN_$NO_2$ | $2.11 \times 10^{3}$ | $2.38 \times 10^{3}$ | $1.13 \times 10^{0}$ | $2.69 \times 10^{2}$ |
| | $NO_2$_CN_$NO_2$ | $1.07 \times 10^{3}$ | $1.70 \times 10^{3}$ | $1.59 \times 10^{0}$ | $6.33 \times 10^{2}$ |
| Substitution $R_{3,6}$ | $NH_2$_CN_H | $3.42 \times 10^{-2}$ | $8.22 \times 10^{2}$ | $8.22 \times 10^{5}$ | $8.21 \times 10^{2}$ |
| | $NH_2$_CN_F | $1.41 \times 10^{0}$ | $7.89 \times 10^{2}$ | $7.89 \times 10^{5}$ | $7.88 \times 10^{2}$ |
| | $NH_2$_CN_CN | $2.31 \times 10^{-2}$ | $1.65 \times 10^{3}$ | $1.65 \times 10^{6}$ | $1.65 \times 10^{3}$ |
| | $NH_2$_CN_$CH_3$ | $7.40 \times 10^{-1}$ | $9.05 \times 10^{2}$ | $9.05 \times 10^{5}$ | $9.05 \times 10^{2}$ |
| | $NH_2$_CN_$NH_2$ | $1.49 \times 10^{-1}$ | $2.04 \times 10^{3}$ | $2.04 \times 10^{6}$ | $2.04 \times 10^{3}$ |
| | $NH_2$_CN_OH | $5.86 \times 10^{2}$ | $1.10 \times 10^{3}$ | $1.88 \times 10^{0}$ | $5.13 \times 10^{2}$ |
| | $NH_2$_CN_$NO_2$ | $6.43 \times 10^{-1}$ | $3.06 \times 10^{3}$ | $3.06 \times 10^{6}$ | $3.06 \times 10^{3}$ |

### 2.2.3 $R_{1,3,5}$, $R_{2,4,6}$: ratio based contrast definition BFS

**Table S11.** Global iteration structures of the BFS procedure on the maximization of the ratio based contrast of the **28M** ⇋ **28R** switch substituting the $R_{1,3,5}$, $R_{2,4,6}$ positions. The static hyper-Rayleigh scattering first hyperpolarizability values of the [28]hexaphyrins are given in a.u.[a]

| **Global iteration 1** | **$R_{1,3,5}$_$R_{2,4,6}$** | **$\beta_{HRS}$(28R)** | **$\beta_{HRS}$(28M)** | **contrast (ratio)** | **contrast (difference)** |
|---|---|---|---|---|---|
| | H_$CH_3$ | $1.29 \times 10^3$ | $3.52 \times 10^2$ | $2.73 \times 10^{-1}$ | $-9.40 \times 10^2$ |
| | F_$CH_3$ | $1.78 \times 10^3$ | $1.85 \times 10^3$ | $1.04 \times 10^0$ | $7.33 \times 10^1$ |
| | CN_$CH_3$ | $4.43 \times 10^3$ | $4.98 \times 10^3$ | $1.13 \times 10^0$ | $5.55 \times 10^2$ |
| Substitution $R_{1,3,5}$ | $CH_3$_$CH_3$ | $1.00 \times 10^{-3}$ | $1.16 \times 10^3$ | $1.16 \times 10^6$ | $1.16 \times 10^3$ |
| | $NH_2$_$CH_3$ | $2.98 \times 10^3$ | $3.63 \times 10^3$ | $1.22 \times 10^0$ | $1.37 \times 10^3$ |
| | OH_$CH_3$ | $3.59 \times 10^3$ | $4.60 \times 10^3$ | $1.28 \times 10^0$ | $1.02 \times 10^3$ |
| | $NO_2$_$CH_3$ | $3.68 \times 10^3$ | $5.92 \times 10^3$ | $1.61 \times 10^0$ | $2.24 \times 10^3$ |
| | $CH_3$_H | $1.29 \times 10^3$ | $1.15 \times 10^3$ | $8.90 \times 10^{-1}$ | $-1.42 \times 10^2$ |
| | $CH_3$_F | $1.78 \times 10^3$ | $6.78 \times 10^2$ | $3.81 \times 10^{-1}$ | $-1.10 \times 10^3$ |
| | $CH_3$_CN | $4.43 \times 10^3$ | $6.17 \times 10^3$ | $1.39 \times 10^0$ | $1.75 \times 10^3$ |
| Substitution $R_{2,4,6}$ | $CH_3$_$CH_3$ | $1.00 \times 10^{-3}$ | $1.16 \times 10^3$ | $1.16 \times 10^6$ | $1.16 \times 10^3$ |
| | $CH_3$_$NH_2$ | $2.98 \times 10^3$ | $3.89 \times 10^3$ | $1.30 \times 10^0$ | $9.06 \times 10^2$ |
| | $CH_3$_OH | $3.59 \times 10^3$ | $5.10 \times 10^3$ | $1.42 \times 10^0$ | $1.51 \times 10^3$ |
| | $CH_3$_$NO_2$ | $3.68 \times 10^3$ | $4.94 \times 10^3$ | $1.34 \times 10^0$ | $1.26 \times 10^3$ |
| **Global iteration 2** | **$R_{1,3,5}$_$R_{2,4,6}$** | **$\beta_{HRS}$(28R)** | **$\beta_{HRS}$(28M)** | **contrast (ratio)** | **contrast (difference)** |
| | H_$CH_3$ | $1.29 \times 10^3$ | $3.52 \times 10^2$ | $2.73 \times 10^{-1}$ | $-9.40 \times 10^2$ |
| | F_$CH_3$ | $1.78 \times 10^3$ | $1.85 \times 10^3$ | $1.04 \times 10^0$ | $7.33 \times 10^1$ |
| | CN_$CH_3$ | $4.43 \times 10^3$ | $4.98 \times 10^3$ | $1.13 \times 10^0$ | $5.55 \times 10^2$ |
| Substitution $R_{1,3,5}$ | $CH_3$_$CH_3$ | $1.00 \times 10^{-3}$ | $1.16 \times 10^3$ | $1.16 \times 10^6$ | $1.16 \times 10^3$ |
| | $NH_2$_$CH_3$ | $2.98 \times 10^3$ | $3.63 \times 10^3$ | $1.22 \times 10^0$ | $1.37 \times 10^3$ |
| | OH_$CH_3$ | $3.59 \times 10^3$ | $4.60 \times 10^3$ | $1.28 \times 10^0$ | $1.02 \times 10^3$ |
| | $NO_2$_$CH_3$ | $3.68 \times 10^3$ | $5.92 \times 10^3$ | $1.61 \times 10^0$ | $2.24 \times 10^3$ |
| | $CH_3$_H | $1.29 \times 10^3$ | $1.15 \times 10^3$ | $8.90 \times 10^{-1}$ | $-1.42 \times 10^2$ |
| | $CH_3$_F | $1.78 \times 10^3$ | $6.78 \times 10^2$ | $3.81 \times 10^{-1}$ | $-1.10 \times 10^3$ |
| | $CH_3$_CN | $4.43 \times 10^3$ | $6.17 \times 10^3$ | $1.39 \times 10^0$ | $1.75 \times 10^3$ |
| Substitution $R_{2,4,6}$ | $CH_3$_$CH_3$ | $1.00 \times 10^{-3}$ | $1.16 \times 10^3$ | $1.16 \times 10^6$ | $1.16 \times 10^3$ |
| | $CH_3$_$NH_2$ | $2.98 \times 10^3$ | $3.89 \times 10^3$ | $1.30 \times 10^0$ | $9.06 \times 10^2$ |
| | $CH_3$_OH | $3.59 \times 10^3$ | $5.10 \times 10^3$ | $1.42 \times 10^0$ | $1.51 \times 10^3$ |
| | $CH_3$_$NO_2$ | $3.68 \times 10^3$ | $4.94 \times 10^3$ | $1.34 \times 10^0$ | $1.26 \times 10^3$ |

[a] These substitution patterns show a $\beta_{HRS}$**(28R)** below 10 a.u.. Their respective contrasts are computed considering a value of 0.001 for the $\beta_{HRS}$**(28R)**. For the difference definition the real value is used to compute the difference.

### 2.2.4 $R_{1,3,5}$, $R_{2,4,6}$: difference based contrast definition BFS

**Table S12.** Global iteration structures of the BFS procedure on the maximization of the difference based contrast of the **28M** ⇋ **28R** switch substituting the $R_{1,3,5}$, $R_{2,4,6}$ positions. The static hyper-Rayleigh scattering first hyperpolarizability values of the [28]hexaphyrins are given in a.u..

| **Global iteration 1** | **$R_{1,3,5}$_$R_{2,4,6}$** | **$\beta_{HRS}$(28R)** | **$\beta_{HRS}$(28M)** | **contrast (ratio)** | **contrast (difference)** |
|---|---|---|---|---|---|
| Substitution $R_{1,3,5}$ | H_$CH_3$ | $1.29 \times 10^3$ | $3.52 \times 10^2$ | $2.73 \times 10^{-1}$ | $-9.40 \times 10^2$ |
| | F_$CH_3$ | $1.78 \times 10^3$ | $1.85 \times 10^3$ | $1.04 \times 10^0$ | $7.33 \times 10^1$ |
| | CN_$CH_3$ | $4.43 \times 10^3$ | $4.98 \times 10^3$ | $1.13 \times 10^0$ | $5.55 \times 10^2$ |
| | $CH_3$_$CH_3$ | $1.34 \times 10^0$ | $1.16 \times 10^3$ | $1.16 \times 10^6$ | $1.16 \times 10^3$ |
| | $NH_2$_$CH_3$ | $2.98 \times 10^3$ | $3.63 \times 10^3$ | $1.22 \times 10^0$ | $6.43 \times 10^2$ |
| | OH_$CH_3$ | $3.59 \times 10^3$ | $4.60 \times 10^3$ | $1.28 \times 10^0$ | $1.02 \times 10^3$ |
| | $NO_2$_$CH_3$ | $3.68 \times 10^3$ | $5.92 \times 10^3$ | $1.61 \times 10^0$ | $2.24 \times 10^3$ |
| Substitution $R_{2,4,6}$ | $NO_2$_H | $3.16 \times 10^3$ | $4.89 \times 10^3$ | $1.54 \times 10^0$ | $1.72 \times 10^3$ |
| | $NO_2$_F | $5.16 \times 10^3$ | $7.41 \times 10^3$ | $1.44 \times 10^0$ | $2.25 \times 10^3$ |
| | $NO_2$_CN | $1.27 \times 10^3$ | $2.27 \times 10^3$ | $1.79 \times 10^0$ | $1.00 \times 10^3$ |
| | $NO_2$_$CH_3$ | $3.68 \times 10^3$ | $5.92 \times 10^3$ | $1.61 \times 10^0$ | $2.24 \times 10^3$ |
| | $NO_2$_$NH_2$ | $1.07 \times 10^4$ | $1.95 \times 10^4$ | $1.82 \times 10^0$ | $8.77 \times 10^3$ |
| | $NO_2$_OH | $9.23 \times 10^3$ | $9.90 \times 10^3$ | $1.07 \times 10^0$ | $6.76 \times 10^2$ |
| | $NO_2$_$NO_2$ | $1.00 \times 10^3$ | $2.38 \times 10^3$ | $2.37 \times 10^0$ | $1.38 \times 10^3$ |
| **Global iteration 2** | **$R_{1,3,5}$_$R_{2,4,6}$** | **$\beta_{HRS}$(28R)** | **$\beta_{HRS}$(28M)** | **contrast (ratio)** | **contrast (difference)** |
| Substitution $R_{2,4,6}$ | $NO_2$_H | $3.16 \times 10^3$ | $4.89 \times 10^3$ | $1.54 \times 10^0$ | $1.72 \times 10^3$ |
| | $NO_2$_F | $5.16 \times 10^3$ | $7.41 \times 10^3$ | $1.44 \times 10^0$ | $2.25 \times 10^3$ |
| | $NO_2$_CN | $1.27 \times 10^3$ | $2.27 \times 10^3$ | $1.79 \times 10^0$ | $1.00 \times 10^3$ |
| | $NO_2$_$CH_3$ | $3.68 \times 10^3$ | $5.92 \times 10^3$ | $1.61 \times 10^0$ | $2.24 \times 10^3$ |
| | $NO_2$_$NH_2$ | $1.07 \times 10^4$ | $1.95 \times 10^4$ | $1.82 \times 10^0$ | $8.77 \times 10^3$ |
| | $NO_2$_OH | $9.23 \times 10^3$ | $9.90 \times 10^3$ | $1.07 \times 10^0$ | $6.76 \times 10^2$ |
| | $NO_2$_$NO_2$ | $1.00 \times 10^3$ | $2.38 \times 10^3$ | $2.37 \times 10^0$ | $1.38 \times 10^3$ |
| Substitution $R_{1,3,5}$ | H_$NH_2$ | $2.78 \times 10^3$ | $2.51 \times 10^3$ | $9.06 \times 10^{-1}$ | $-2.62 \times 10^2$ |
| | F_$NH_2$ | $1.78 \times 10^3$ | $2.09 \times 10^3$ | $1.17 \times 10^0$ | $3.06 \times 10^2$ |
| | CN_$NH_2$ | $1.15 \times 10^4$ | $1.63 \times 10^4$ | $1.41 \times 10^0$ | $4.74 \times 10^3$ |
| | $CH_3$_$NH_2$ | $2.98 \times 10^3$ | $3.89 \times 10^3$ | $1.30 \times 10^0$ | $9.06 \times 10^2$ |
| | $NH_2$_$NH_2$ | $2.70 \times 10^3$ | $2.21 \times 10^3$ | $8.19 \times 10^{-1}$ | $-4.90 \times 10^2$ |
| | OH_$NH_2$ | $1.46 \times 10^3$ | $2.43 \times 10^3$ | $1.67 \times 10^0$ | $9.71 \times 10^2$ |
| | $NO_2$_$NH_2$ | $1.07 \times 10^4$ | $1.95 \times 10^4$ | $1.82 \times 10^0$ | $8.77 \times 10^3$ |

## 2.2.5 $R_{1,3}$, $R_{4,6}$, $R_2$, $R_5$: ratio based contrast definition BFS

**Table S13.** Global iteration structures of the BFS procedure on the maximization of the ratio based contrast of the **28M** ⇋ **28R** switch substituting the $R_{1,3,5}$, $R_{2,4,6}$ positions. The static hyper-Rayleigh scattering first hyperpolarizability values of the [28]hexaphyrins are given in a.u.[a]

| Global iteration 1 | $R_{1,3}$_$R_2$_$R_{4,6}$_$R_5$ | $\beta_{HRS}$(28R) | $\beta_{HRS}$(28M) | contrast (ratio) | contrast (difference) |
|---|---|---|---|---|---|
| Substitution $R_{1,3}$ | H_$NO_2$_CN_$NH_2$ | $6.77 \times 10^3$ | $6.47 \times 10^3$ | $9.56 \times 10^{-1}$ | $-2.97 \times 10^2$ |
| | F_$NO_2$_CN_$NH_2$ | $9.20 \times 10^3$ | $5.98 \times 10^3$ | $6.51 \times 10^{-1}$ | $-3.21 \times 10^3$ |
| | CN_$NO_2$_CN_$NH_2$ | $3.39 \times 10^3$ | $3.99 \times 10^3$ | $1.18 \times 10^0$ | $6.04 \times 10^2$ |
| | $CH_3$_$NO_2$_CN_$NH_2$ | $6.25 \times 10^3$ | $8.03 \times 10^3$ | $1.28 \times 10^0$ | $1.78 \times 10^3$ |
| | $NH_2$_$NO_2$_CN_$NH_2$ | $1.12 \times 10^4$ | $1.32 \times 10^4$ | $1.17 \times 10^0$ | $1.95 \times 10^3$ |
| | OH_$NO_2$_CN_$NH_2$ | $8.38 \times 10^3$ | $9.81 \times 10^3$ | $1.17 \times 10^0$ | $1.43 \times 10^3$ |
| | $NO_2$_$NO_2$_CN_$NH_2$ | $2.82 \times 10^3$ | $3.22 \times 10^3$ | $1.14 \times 10^0$ | $4.01 \times 10^2$ |
| Substitution $R_5$ | $CH_3$_$NO_2$_CN_H | $3.77 \times 10^3$ | $4.57 \times 10^3$ | $1.21 \times 10^0$ | $8.01 \times 10^2$ |
| | $CH_3$_$NO_2$_CN_F | $3.80 \times 10^3$ | $4.54 \times 10^3$ | $1.19 \times 10^0$ | $7.41 \times 10^2$ |
| | $CH_3$_$NO_2$_CN_CN | $2.35 \times 10^3$ | $4.62 \times 10^3$ | $1.96 \times 10^0$ | $2.26 \times 10^3$ |
| | $CH_3$_$NO_2$_CN_$CH_3$ | $4.08 \times 10^3$ | $4.47 \times 10^3$ | $1.09 \times 10^0$ | $3.87 \times 10^2$ |
| | $CH_3$_$NO_2$_CN_$NH_2$ | $6.25 \times 10^3$ | $8.03 \times 10^3$ | $1.28 \times 10^0$ | $1.78 \times 10^3$ |
| | $CH_3$_$NO_2$_CN_OH | $4.61 \times 10^3$ | $4.30 \times 10^3$ | $9.34 \times 10^{-1}$ | $-3.04 \times 10^2$ |
| | $CH_3$_$NO_2$_CN_$NO_2$ | $2.39 \times 10^3$ | $5.53 \times 10^3$ | $2.31 \times 10^0$ | $3.14 \times 10^3$ |
| Substitution $R_2$ | $CH_3$_H_CN_$NO_2$ | $1.98 \times 10^3$ | $6.40 \times 10^3$ | $3.24 \times 10^0$ | $4.42 \times 10^3$ |
| | $CH_3$_F_CN_$NO_2$ | $1.92 \times 10^3$ | $5.60 \times 10^3$ | $2.91 \times 10^0$ | $3.68 \times 10^3$ |
| | $CH_3$_CN_CN_$NO_2$ | $2.58 \times 10^3$ | $7.12 \times 10^3$ | $2.76 \times 10^0$ | $4.54 \times 10^3$ |
| | $CH_3$_$CH_3$_CN_$NO_2$ | $2.27 \times 10^3$ | $7.00 \times 10^3$ | $3.09 \times 10^0$ | $4.73 \times 10^3$ |
| | $CH_3$_$NH_2$_CN_$NO_2$ | $2.85 \times 10^3$ | $4.66 \times 10^3$ | $1.64 \times 10^0$ | $1.81 \times 10^3$ |
| | $CH_3$_OH_CN_$NO_2$ | $2.39 \times 10^3$ | $3.61 \times 10^3$ | $1.51 \times 10^0$ | $1.23 \times 10^3$ |
| | $CH_3$_$NO_2$_CN_$NO_2$ | $2.39 \times 10^3$ | $5.53 \times 10^3$ | $2.31 \times 10^0$ | $3.14 \times 10^3$ |
| Substitution $R_{4,6}$ | $CH_3$_H_H_$NO_2$ | $3.61 \times 10^3$ | $4.07 \times 10^3$ | $8.87 \times 10^{-1}$ | $4.62 \times 10^2$ |
| | $CH_3$_H_F_$NO_2$ | $7.01 \times 10^3$ | $2.87 \times 10^3$ | $4.10 \times 10^{-1}$ | $-4.14 \times 10^3$ |
| | $CH_3$_H_CN_$NO_2$ | $1.98 \times 10^3$ | $6.40 \times 10^3$ | $3.24 \times 10^0$ | $4.42 \times 10^3$ |
| | $CH_3$_H_$CH_3$_$NO_2$ | $4.44 \times 10^3$ | $2.91 \times 10^3$ | $6.56 \times 10^{-1}$ | $-1.52 \times 10^3$ |
| | $CH_3$_H_$NH_2$_$NO_2$ | $5.76 \times 10^3$ | $1.44 \times 10^3$ | $2.51 \times 10^{-1}$ | $-4.31 \times 10^3$ |
| | $CH_3$_H_OH_$NO_2$ | $9.73 \times 10^3$ | $1.82 \times 10^3$ | $1.87 \times 10^{-1}$ | $-7.92 \times 10^3$ |
| | $CH_3$_H_$NO_2$_$NO_2$ | $1.39 \times 10^3$ | $6.54 \times 10^3$ | $4.70 \times 10^0$ | $5.15 \times 10^3$ |

[a] These substitution patterns show a $\beta_{HRS}$(**28R**) below 10 a.u.. Their respective contrasts are computed considering a value of 0.001 for the $\beta_{HRS}$(**28R**). For the difference definition the real value is used to compute the difference.

**Table S13.** Global iteration structures of the BFS procedure on the maximization of the ratio based contrast of the **28M** ⇋ **28R** switch substituting the $R_{1,3,5}$, $R_{2,4,6}$ positions. The static hyper-Rayleigh scattering first hyperpolarizability values of the [28]hexaphyrins are given in a.u..

| Global iteration 2 | $R_{1,3}$_$R_2$_$R_{4,6}$_$R_5$ | $\beta_{HRS}$(28R) | $\beta_{HRS}$(28M) | contrast (ratio) | contrast (difference) |
|---|---|---|---|---|---|
| Substitution $R_{4,6}$ | $CH_3$_H_H_$NO_2$ | $3.61 \times 10^3$ | $4.07 \times 10^3$ | $8.87 \times 10^{-1}$ | $4.62 \times 10^2$ |
| | $CH_3$_H_F_$NO_2$ | $7.01 \times 10^3$ | $2.87 \times 10^3$ | $4.10 \times 10^{-1}$ | $-4.14 \times 10^3$ |
| | $CH_3$_H_CN_$NO_2$ | $1.98 \times 10^3$ | $6.40 \times 10^3$ | $3.24 \times 10^0$ | $4.42 \times 10^3$ |
| | $CH_3$_H_$CH_3$_$NO_2$ | $4.44 \times 10^3$ | $2.91 \times 10^3$ | $6.56 \times 10^{-1}$ | $-1.52 \times 10^3$ |
| | $CH_3$_H_$NH_2$_$NO_2$ | $5.76 \times 10^3$ | $1.44 \times 10^3$ | $2.51 \times 10^{-1}$ | $-4.31 \times 10^3$ |
| | $CH_3$_H_OH_$NO_2$ | $9.73 \times 10^3$ | $1.82 \times 10^3$ | $1.87 \times 10^{-1}$ | $-7.92 \times 10^3$ |
| | $CH_3$_H_$NO_2$_$NO_2$ | $1.39 \times 10^3$ | $6.54 \times 10^3$ | $4.70 \times 10^0$ | $5.15 \times 10^3$ |
| Substitution $R_5$ | $CH_3$_H_$NO_2$_H | $1.45 \times 10^3$ | $3.47 \times 10^3$ | $2.39 \times 10^0$ | $2.02 \times 10^3$ |
| | $CH_3$_H_$NO_2$_F | $1.57 \times 10^3$ | $2.99 \times 10^3$ | $1.91 \times 10^0$ | $1.42 \times 10^3$ |
| | $CH_3$_H_$NO_2$_CN | $1.51 \times 10^3$ | $2.96 \times 10^3$ | $1.96 \times 10^0$ | $1.45 \times 10^3$ |
| | $CH_3$_H_$NO_2$_$CH_3$ | $1.55 \times 10^3$ | $3.32 \times 10^3$ | $2.14 \times 10^0$ | $1.77 \times 10^3$ |
| | $CH_3$_H_$NO_2$_$NH_2$ | $3.94 \times 10^3$ | $3.05 \times 10^3$ | $7.75 \times 10^{-1}$ | $-8.88 \times 10^2$ |
| | $CH_3$_H_$NO_2$_OH | $1.96 \times 10^3$ | $2.88 \times 10^3$ | $1.47 \times 10^0$ | $9.20 \times 10^2$ |
| | $CH_3$_H_$NO_2$_$NO_2$ | $1.39 \times 10^3$ | $6.54 \times 10^3$ | $4.70 \times 10^0$ | $5.15 \times 10^3$ |
| Substitution $R_2$ | $CH_3$_H_$NO_2$_$NO_2$ | $1.39 \times 10^3$ | $6.54 \times 10^3$ | $4.70 \times 10^0$ | $5.15 \times 10^3$ |
| | $CH_3$_F_$NO_2$_$NO_2$ | $1.37 \times 10^3$ | $2.72 \times 10^3$ | $1.99 \times 10^0$ | $1.36 \times 10^3$ |
| | $CH_3$_CN_$NO_2$_$NO_2$ | $2.48 \times 10^3$ | $7.67 \times 10^3$ | $3.09 \times 10^0$ | $5.19 \times 10^3$ |
| | $CH_3$_$CH_3$_$NO_2$_$NO_2$ | $1.44 \times 10^3$ | $2.76 \times 10^3$ | $1.92 \times 10^0$ | $1.32 \times 10^3$ |
| | $CH_3$_$NH_2$_$NO_2$_$NO_2$ | $2.19 \times 10^3$ | $3.58 \times 10^3$ | $1.64 \times 10^0$ | $1.39 \times 10^3$ |
| | $CH_3$_OH_$NO_2$_$NO_2$ | $1.63 \times 10^3$ | $2.97 \times 10^3$ | $1.82 \times 10^0$ | $1.34 \times 10^3$ |
| | $CH_3$_$NO_2$_$NO_2$_$NO_2$ | $5.90 \times 10^3$ | $1.43 \times 10^4$ | $2.42 \times 10^0$ | $8.35 \times 10^3$ |
| Substitution $R_{1,3}$ | H_H_$NO_2$_$NO_2$ | $1.40 \times 10^3$ | $5.41 \times 10^3$ | $3.87 \times 10^0$ | $4.01 \times 10^3$ |
| | F_H_$NO_2$_$NO_2$ | $2.80 \times 10^3$ | $8.47 \times 10^3$ | $3.02 \times 10^0$ | $5.66 \times 10^3$ |
| | CN_H_$NO_2$_$NO_2$ | $2.32 \times 10^3$ | $2.36 \times 10^3$ | $1.02 \times 10^0$ | $3.57 \times 10^1$ |
| | $CH_3$_H_$NO_2$_$NO_2$ | $1.39 \times 10^3$ | $6.54 \times 10^3$ | $4.70 \times 10^0$ | $5.15 \times 10^3$ |
| | $NH_2$_H_$NO_2$_$NO_2$ | $5.06 \times 10^3$ | $5.90 \times 10^3$ | $1.17 \times 10^0$ | $8.43 \times 10^2$ |
| | OH_H_$NO_2$_$NO_2$ | $3.39 \times 10^3$ | $6.72 \times 10^3$ | $1.98 \times 10^0$ | $3.33 \times 10^3$ |
| | $NO_2$_H_$NO_2$_$NO_2$ | $1.51 \times 10^3$ | $1.80 \times 10^3$ | $1.19 \times 10^0$ | $2.92 \times 10^2$ |

[a] These substitution patterns show a $\beta_{HRS}$**(28R)** below 10 a.u.. Their respective contrasts are computed considering a value of 0.001 for the $\beta_{HRS}$**(28R)**. For the difference definition the real value is used to compute the difference.

### 2.2.6 $R_{1,3}$, $R_{4,6}$, $R_2$, $R_5$: difference based contrast definition BFS

**Table S14.** Global iteration structures of the BFS procedure on the maximization of the difference based contrast of the **28M** ⇋ **28R** switch substituting the $R_{1,3,5}$, $R_{2,4,6}$ positions. The static hyper-Rayleigh scattering first hyperpolarizability values of the [28]hexaphyrins are given in a.u..

| Global iteration 1 | $R_{1,3}$_$R_2$_$R_{4,6}$_$R_5$ | $\beta_{HRS}$(28R) | $\beta_{HRS}$(28M) | contrast (ratio) | contrast (difference) |
|---|---|---|---|---|---|
| Substitution $R_{1,3}$ | H_$NO_2$_CN_$NH_2$ | $6.77 \times 10^3$ | $6.47 \times 10^3$ | $9.56 \times 10^{-1}$ | $-2.97 \times 10^2$ |
| | F_$NO_2$_CN_$NH_2$ | $9.20 \times 10^3$ | $5.98 \times 10^3$ | $6.51 \times 10^{-1}$ | $-3.21 \times 10^3$ |
| | CN_$NO_2$_CN_$NH_2$ | $3.39 \times 10^3$ | $3.99 \times 10^3$ | $1.18 \times 10^0$ | $6.04 \times 10^2$ |
| | $CH_3$_$NO_2$_CN_$NH_2$ | $6.25 \times 10^3$ | $8.03 \times 10^3$ | $1.28 \times 10^0$ | $1.78 \times 10^3$ |
| | $NH_2$_$NO_2$_CN_$NH_2$ | $1.12 \times 10^4$ | $1.32 \times 10^4$ | $1.17 \times 10^0$ | $1.95 \times 10^3$ |
| | OH_$NO_2$_CN_$NH_2$ | $8.38 \times 10^3$ | $9.81 \times 10^3$ | $1.17 \times 10^0$ | $1.43 \times 10^3$ |
| | $NO_2$_$NO_2$_CN_$NH_2$ | $2.82 \times 10^3$ | $3.22 \times 10^3$ | $1.14 \times 10^0$ | $4.01 \times 10^2$ |
| Substitution $R_5$ | $NH_2$_$NO_2$_CN_H | $1.32 \times 10^4$ | $1.25 \times 10^4$ | $9.48 \times 10^{-1}$ | $-6.84 \times 10^2$ |
| | $NH_2$_$NO_2$_CN_F | $1.03 \times 10^4$ | $1.36 \times 10^4$ | $1.32 \times 10^0$ | $3.27 \times 10^3$ |
| | $NH_2$_$NO_2$_CN_CN | $1.41 \times 10^4$ | $1.23 \times 10^4$ | $8.71 \times 10^{-1}$ | $-1.83 \times 10^3$ |
| | $NH_2$_$NO_2$_CN_$CH_3$ | $1.31 \times 10^4$ | $1.27 \times 10^4$ | $9.69 \times 10^{-1}$ | $-4.05 \times 10^2$ |
| | $NH_2$_$NO_2$_CN_$NH_2$ | $1.12 \times 10^4$ | $1.32 \times 10^4$ | $1.17 \times 10^0$ | $1.95 \times 10^3$ |
| | $NH_2$_$NO_2$_CN_OH | $1.01 \times 10^4$ | $1.36 \times 10^4$ | $1.35 \times 10^0$ | $3.53 \times 10^3$ |
| | $NH_2$_$NO_2$_CN_$NO_2$ | $1.53 \times 10^4$ | $1.20 \times 10^4$ | $7.88 \times 10^{-1}$ | $-3.23 \times 10^3$ |
| Substitution $R_2$ | $NH_2$_H_CN_OH | $4.96 \times 10^3$ | $7.10 \times 10^3$ | $1.43 \times 10^0$ | $2.14 \times 10^3$ |
| | $NH_2$_F_CN_OH | $4.70 \times 10^3$ | $6.15 \times 10^3$ | $1.31 \times 10^0$ | $1.45 \times 10^3$ |
| | $NH_2$_CN_CN_OH | $1.05 \times 10^4$ | $1.60 \times 10^4$ | $1.52 \times 10^0$ | $5.48 \times 10^3$ |
| | $NH_2$_$CH_3$_CN_OH | $5.03 \times 10^3$ | $7.03 \times 10^3$ | $1.40 \times 10^0$ | $2.00 \times 10^3$ |
| | $NH_2$_$NH_2$_CN_OH | $4.04 \times 10^3$ | $4.53 \times 10^3$ | $1.12 \times 10^0$ | $4.91 \times 10^2$ |
| | $NH_2$_OH_CN_OH | $4.66 \times 10^3$ | $5.27 \times 10^3$ | $1.13 \times 10^0$ | $6.15 \times 10^2$ |
| | $NH_2$_$NO_2$_CN_OH | $1.01 \times 10^4$ | $1.36 \times 10^4$ | $1.35 \times 10^0$ | $3.53 \times 10^3$ |
| Substitution $R_{4,6}$ | $NH_2$_CN_H_OH | $6.11 \times 10^3$ | $5.89 \times 10^3$ | $9.65 \times 10^{-1}$ | $-2.16 \times 10^2$ |
| | $NH_2$_CN_F_OH | $4.82 \times 10^3$ | $3.96 \times 10^3$ | $8.21 \times 10^{-1}$ | $-8.62 \times 10^2$ |
| | $NH_2$_CN_CN_OH | $1.05 \times 10^4$ | $1.60 \times 10^4$ | $1.52 \times 10^0$ | $5.48 \times 10^3$ |
| | $NH_2$_CN_$CH_3$_OH | $6.63 \times 10^3$ | $4.35 \times 10^3$ | $6.56 \times 10^{-1}$ | $-2.28 \times 10^3$ |
| | $NH_2$_CN_$NH_2$_OH | $6.64 \times 10^3$ | $2.47 \times 10^3$ | $3.72 \times 10^{-1}$ | $-4.17 \times 10^3$ |
| | $NH_2$_CN_OH_OH | $4.13 \times 10^3$ | $2.94 \times 10^3$ | $7.11 \times 10^{-1}$ | $-1.19 \times 10^3$ |
| | $NH_2$_CN_$NO_2$_OH | $1.16 \times 10^4$ | $1.40 \times 10^4$ | $1.20 \times 10^0$ | $2.37 \times 10^3$ |

**Table S14.** Global iteration structures of the BFS procedure on the maximization of the difference based contrast of the **28M** ⇋ **28R** switch substituting the $R_{1,3,5}$, $R_{2,4,6}$ positions. The static hyper-Rayleigh scattering first hyperpolarizability values of the [28]hexaphyrins are given in a.u..

| Global iteration 2 | $R_{1,3}$_$R_2$_$R_{4,6}$_$R_5$ | $\beta_{HRS}$(28R) | $\beta_{HRS}$(28M) | contrast (ratio) | contrast (difference) |
|---|---|---|---|---|---|
| Substitution $R_{4,6}$ | $NH_2$_CN_H_OH | $6.11 \times 10^3$ | $5.89 \times 10^3$ | $9.65 \times 10^{-1}$ | $-2.16 \times 10^2$ |
| | $NH_2$_CN_F_OH | $4.82 \times 10^3$ | $3.96 \times 10^3$ | $8.21 \times 10^{-1}$ | $-8.62 \times 10^2$ |
| | $NH_2$_CN_CN_OH | $1.05 \times 10^4$ | $1.60 \times 10^4$ | $1.52 \times 10^0$ | $5.48 \times 10^3$ |
| | $NH_2$_CN_$CH_3$_OH | $6.63 \times 10^3$ | $4.35 \times 10^3$ | $6.56 \times 10^{-1}$ | $-2.28 \times 10^3$ |
| | $NH_2$_CN_$NH_2$_OH | $6.64 \times 10^3$ | $2.47 \times 10^3$ | $3.72 \times 10^{-1}$ | $-4.17 \times 10^3$ |
| | $NH_2$_CN_OH_OH | $4.13 \times 10^3$ | $2.94 \times 10^3$ | $7.11 \times 10^{-1}$ | $-1.19 \times 10^3$ |
| | $NH_2$_CN_$NO_2$_OH | $1.16 \times 10^4$ | $1.40 \times 10^4$ | $1.20 \times 10^0$ | $2.37 \times 10^3$ |
| Substitution $R_5$ | $NH_2$_CN_CN_H | $1.32 \times 10^4$ | $1.54 \times 10^4$ | $1.17 \times 10^0$ | $2.20 \times 10^3$ |
| | $NH_2$_CN_CN_F | $1.08 \times 10^4$ | $1.51 \times 10^4$ | $1.41 \times 10^0$ | $4.37 \times 10^3$ |
| | $NH_2$_CN_CN_CN | $1.32 \times 10^4$ | $1.50 \times 10^4$ | $1.14 \times 10^0$ | $1.79 \times 10^3$ |
| | $NH_2$_CN_CN_$CH_3$ | $1.26 \times 10^4$ | $1.52 \times 10^4$ | $1.21 \times 10^0$ | $2.60 \times 10^3$ |
| | $NH_2$_CN_CN_$NH_2$ | $1.15 \times 10^4$ | $1.59 \times 10^4$ | $1.38 \times 10^0$ | $4.33 \times 10^3$ |
| | $NH_2$_CN_CN_OH | $1.05 \times 10^4$ | $1.60 \times 10^4$ | $1.52 \times 10^0$ | $5.48 \times 10^3$ |
| | $NH_2$_CN_CN_$NO_2$ | $1.38 \times 10^4$ | $1.47 \times 10^4$ | $1.07 \times 10^0$ | $9.07 \times 10^2$ |
| Substitution $R_2$ | $NH_2$_H_CN_OH | $4.96 \times 10^3$ | $7.10 \times 10^3$ | $1.43 \times 10^0$ | $2.14 \times 10^3$ |
| | $NH_2$_F_CN_OH | $4.70 \times 10^3$ | $6.15 \times 10^3$ | $1.31 \times 10^0$ | $1.45 \times 10^3$ |
| | $NH_2$_CN_CN_OH | $1.05 \times 10^4$ | $1.60 \times 10^4$ | $1.52 \times 10^0$ | $5.48 \times 10^3$ |
| | $NH_2$_$CH_3$_CN_OH | $5.03 \times 10^3$ | $7.03 \times 10^3$ | $1.40 \times 10^0$ | $2.00 \times 10^3$ |
| | $NH_2$_$NH_2$_CN_OH | $4.04 \times 10^3$ | $4.53 \times 10^3$ | $1.12 \times 10^0$ | $4.91 \times 10^2$ |
| | $NH_2$_OH_CN_OH | $4.66 \times 10^3$ | $5.27 \times 10^3$ | $1.13 \times 10^0$ | $6.15 \times 10^2$ |
| | $NH_2$_$NO_2$_CN_OH | $1.01 \times 10^4$ | $1.36 \times 10^4$ | $1.35 \times 10^0$ | $3.53 \times 10^3$ |
| Substitution $R_{1,3}$ | H_CN_CN_OH | $6.09 \times 10^3$ | $5.09 \times 10^3$ | $8.37 \times 10^{-1}$ | $-9.94 \times 10^2$ |
| | F_CN_CN_OH | $8.21 \times 10^3$ | $7.75 \times 10^3$ | $9.45 \times 10^{-1}$ | $-4.55 \times 10^2$ |
| | CN_CN_CN_OH | $2.72 \times 10^3$ | $3.01 \times 10^3$ | $1.11 \times 10^0$ | $2.91 \times 10^2$ |
| | $CH_3$_CN_CN_OH | $4.98 \times 10^3$ | $6.53 \times 10^3$ | $1.31 \times 10^0$ | $1.55 \times 10^3$ |
| | $NH_2$_CN_CN_OH | $1.05 \times 10^4$ | $1.60 \times 10^4$ | $1.52 \times 10^0$ | $5.48 \times 10^3$ |
| | OH_CN_CN_OH | $7.93 \times 10^3$ | $1.13 \times 10^4$ | $1.42 \times 10^0$ | $3.35 \times 10^3$ |
| | $NO_2$_CN_CN_OH | $2.20 \times 10^3$ | $2.59 \times 10^3$ | $1.18 \times 10^0$ | $3.90 \times 10^2$ |
